# A combination of improved differential and global RNA-seq reveals pervasive transcription initiation and events in all stages of the life-cycle of functional RNAs in *Propionibacterium acnes*, a major contributor to wide-spread human disease

**DOI:** 10.1186/1471-2164-14-620

**Published:** 2013-09-14

**Authors:** Yu-fei Lin, David Romero A, Shuang Guan, Lira Mamanova, Kenneth J McDowall

**Affiliations:** 1 Astbury Centre for Structural Molecular Biology, School of Molecular and Cellular Biology, Faculty of Biological Sciences, University of Leeds, Leeds LS2 9JT, UK; 2 The Wellcome Trust Sanger Institute, Wellcome Trust Genome Campus, Hinxton, Cambridge CB10 1SA, UK

**Keywords:** Pervasive transcription, Leaderless mRNA, Small non-protein-coding RNAs, RNA degradation and processing, Promoters and regulatory elements, Transcriptional landscape, Differential and global RNA-seq

## Abstract

**Background:**

Sequencing of the genome of *Propionibacterium acnes* produced a catalogue of genes many of which enable this organism to colonise skin and survive exposure to the elements. Despite this platform, there was little understanding of the gene regulation that gives rise to an organism that has a major impact on human health and wellbeing and causes infections beyond the skin. To address this situation, we have undertaken a genome–wide study of gene regulation using a combination of improved differential and global RNA-sequencing and an analytical approach that takes into account the inherent noise within the data.

**Results:**

We have produced nucleotide-resolution transcriptome maps that identify and differentiate sites of transcription initiation from sites of stable RNA processing and mRNA cleavage. Moreover, analysis of these maps provides strong evidence for ‘pervasive’ transcription and shows that contrary to initial indications it is not biased towards the production of antisense RNAs. In addition, the maps reveal an extensive array of riboswitches, leaderless mRNAs and small non-protein-coding RNAs alongside vegetative promoters and post-transcriptional events, which includes unusual tRNA processing. The identification of such features will inform models of complex gene regulation, as illustrated here for ribonucleotide reductases and a potential quorum-sensing, two-component system.

**Conclusions:**

The approach described here, which is transferable to any bacterial species, has produced a step increase in whole-cell knowledge of gene regulation in *P. acnes*. Continued expansion of our maps to include transcription associated with different growth conditions and genetic backgrounds will provide a new platform from which to computationally model the gene expression that determines the physiology of *P. acnes* and its role in human disease.

## Background

*Propionibacterium acnes* is a member of the Actinobacteria, a phylum that includes *Streptomyces*, *Mycobacterium* and *Corynebacterium*. It is renown through its associated with acne vulgaris [1], the most common of all human skin diseases, as well as life-threatening diseases [2], such as meningitis [3] and endocarditis [4]. Most recently, evidence has emerged that *P. acnes* infection causes chronic back pain following physical injury [5]. More usually, it is a harmless commensal of the skin, residing ubiquitously in sebaceous follicles [6]. To adapt, colonise and survive, *P. acnes* needs to sense and respond to changes in its natural environment [7], yet almost nothing is known about how the physiology and growth of *P. acnes* is shaped by regulated gene expression. The 2.6 Mbp genome of *P. acnes* strain KPA171202, the first to be sequenced, contains 2,333 annotated genes [8]. However, the organisation and regulation of the transcription units within this clinically important organism remained to be determined experimentally. Indeed, prior to the work reported here, no transcription start sites (TSSs) had been mapped. Other key aspects of gene regulation for which no information was available were mRNA turnover [9], which ensures translation follows programs of transcription, the generation of RNA components of the translational machinery [10,11], and the prevalence of small regulatory RNAs [12]. Recently, the importance of understanding *P. acnes* gene regulation was exemplified by a study that found the ability of different strains of *P. acnes* to cause disease stems from divergence in the expression as well as the content of their genomes [13].

In the first part of this report, we describe improved global and differential RNA-sequencing (RNA-seq) approaches (Additional file 1) and their use in the construction of the first nucleotide-resolution transcriptome maps of *P. acnes* KPA171202, maps that show sites of RNA processing and degradation in addition to sites of transcription initiation. In the second part, we show how analysis of these maps can shed light on many of the key steps in bacterial gene expression and regulation. We describe ‘pervasive’ transcription, riboswitches, leaderless mRNAs, small non-protein-coding RNAs, post-transcriptional control and unusual tRNA processing. Expansion of these maps to incorporate transcriptional landscapes under different conditions and genetic backgrounds will ultimately reveal how regulated gene expression shapes the physiology and growth of *P. acnes*. As per the original differential (dRNA-seq) approach [14], we distinguished 5′ ends corresponding to transcription initiation from those generated through RNA processing and degradation on the basis of their 5′-phosphorylation status. This was done here, however, using tobacco acid pyrophosphatase (TAP), an enzyme that converts a 5′ triphosphate to monophosphate [15], prior to constructing and sequencing cDNA libraries of native 5′-end segments (Additional file 1). The vast majority of primary transcripts are synthesised with a 5′-triphosphate group, while the 5′ ends of most ‘secondary’ transcripts, *i.e.* those generated through RNA processing and degradation, have a monophosphate group [9,16,17]. Thus, an increased number of sequencing reads from a 5′ end following TAP treatment is an identifier of a transcription start site (TSS). The original dRNA-seq approach differentiated 5′ ends using Terminator™ exonuclease (TEX), which removes 5′-monophosphorylated transcripts [14]. To determine 3′ as well as 5′ boundaries and the abundance of transcripts, aliquots of some RNA samples were also analysed using a global RNA-sequencing (gRNA-seq) approach (Additional file 1) that avoids the use of PCR [18]. As indicated above, dRNA-seq identifies native 5′-ends only.

## Results

### Overview of approach

We analysed the transcriptomes of duplicate cultures of *P. acnes* strain KPA171202 grown exponentially in Holland’s Synthetic Medium (Additional file 2). This defined medium was chosen as it supports highly reproducible growth, which reduces experimental variation and in turn allows underlying features to be identified with increased certainty. It can also be modified easily to investigate the contribution of individual components to growth and cellular physiology. To assess the sensitivity of our dRNA-seq approach to changes in gene expression, samples were taken following subculture with and without potassium downshift (*i.e.* removal of potassium sources from the medium). We choose this change because of its long-established relevance to skin, *e.g.* potassium levels fluctuate as a result of perspiration [19] and potassium deficiency is associated with dermatoses [20]. Moreover, *P. acnes* genes inducible by potassium downshift were predictable from prior studies of other bacteria [21]. Thus, the differential approach described here used eight cDNA libraries; 2 replicates × 2 conditions × 2 treatments (minus or plus TAP treatment). 3 to 6 million reads were obtained for each library and mapped onto the *P. acnes* genome. Next we counted, for each library, the number of times each genome position was the first nucleotide in sequence reads, *i.e.* associated with a 5′ end *in vivo*. Then the reads corresponding to minus and plus TAP treatment were compared using M-A scatterplots for each replicate and condition. Samples from one of the cultures were also analysed by 5′ RACE, high-density microarrays and gRNA-seq to allow comparison with more widely used approaches.

### Transcription start sites

**Figure 1:**
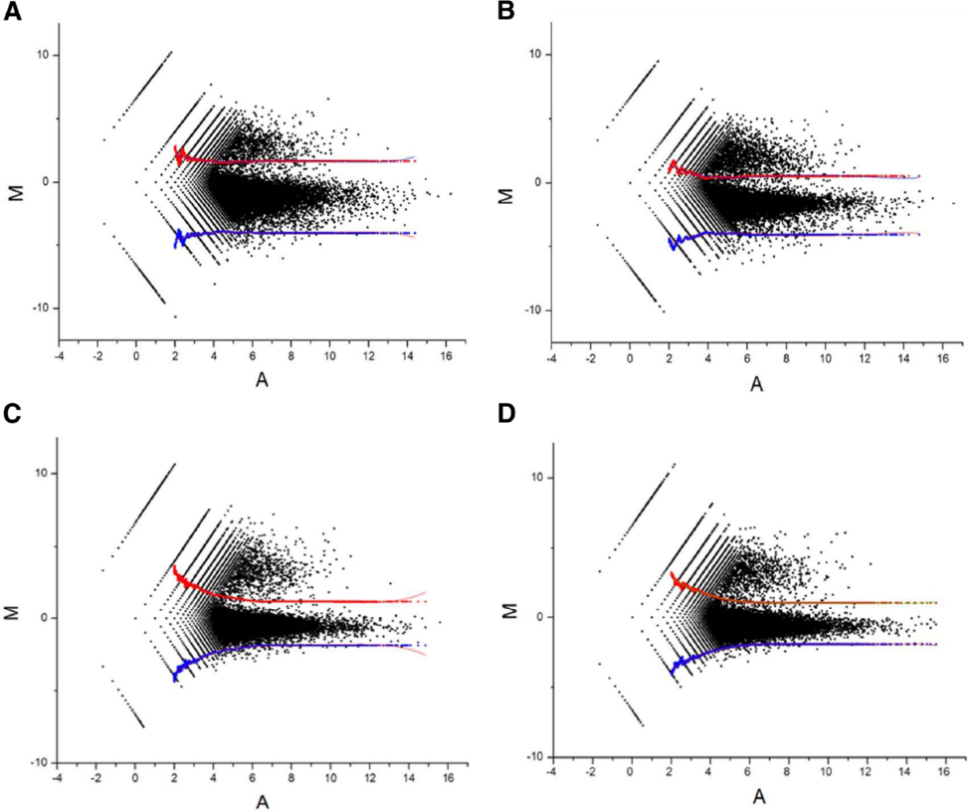
**M-A scatterplots of values from the differential RNA-seq analysis. (A)** and **(B)** show data for cells sub-cultured without and with a potassium downshift, respectively. **(C)** and **(D)**, as **(A)** and **(B)**, except data is for a duplicate starter culture. The M values correspond to Log_2_ (plus/minus) and A values to (Log_2_ plus + Log_2_ minus)/2, where minus and plus refer to the number of reads before and after treatment with TAP. For further details, see *Methods*. The red and blue points represent the upper and lower boundaries, respectively, of an envelope that contains sites of processing and degradation. The upper and lower boundaries were defined by the equation μ ± 3σ, where μ and σ are the average and standard deviation, respectively, of M in a moving window of 5,000 data-points sorted in ascending order of A [22,23].

For each replicate and condition, M-A scatterplots [where M = Log_2_ (reads plus/minus TAP treatment), and A = (log_2_ plus + log_2_ minus)/2] revealed a population of values that centred close to an M value of 0, corresponding to sites of processing and degradation, and another with higher M values, corresponding to transcription start sites (Figure 1). The envelope of values corresponding to sites of processing and degradation was defined by using the standard deviation of M values in windows of ascending A values [22,23]. By this simple approach we were able to take into account the inherent noise within the dRNA-seq data (Figure 1). 5′ ends with M values above the envelope were then identified. This corresponded to around 6 thousand of the 600 thousand positions in each comparison. To increase the power of our TSS analysis, we did not distinguish between samples from subcultures with and without the potassium downshift. Positions with M values above the envelope in each of the four experiments (2 duplicates × 2 conditions) were designated positions of transcription initiation, and positions within 8 nt of each other were classified as belonging to the same TSS. With regard to the latter, it is well established that many promoters can initiate transcription at more than one nucleotide position [24]. For the few genes that showed a change in gene expression following potassium downshift, the stringency of the analysis was reduced to the condition under which transcription could be detected most readily. By this approach we identified 4,058 TSSs (Additional file 3). The probability of a position being outside the envelope in each of four comparisons by chance is less than 1 in 100 million (6/600 to the power 4). The latter number vastly exceeding the total of 5′ ends identified. Moreover, the identified TSSs were associated with low p-values (on average 6.5E-07, see Additional file 3) as determined using Rank Products Analysis [25,26], which is a statistical method specifically developed to analyse data that is based on replicated experiments and compares two outputs, in our case RNA-seq reads obtained with and without TAP treatment.

**Figure 2:**
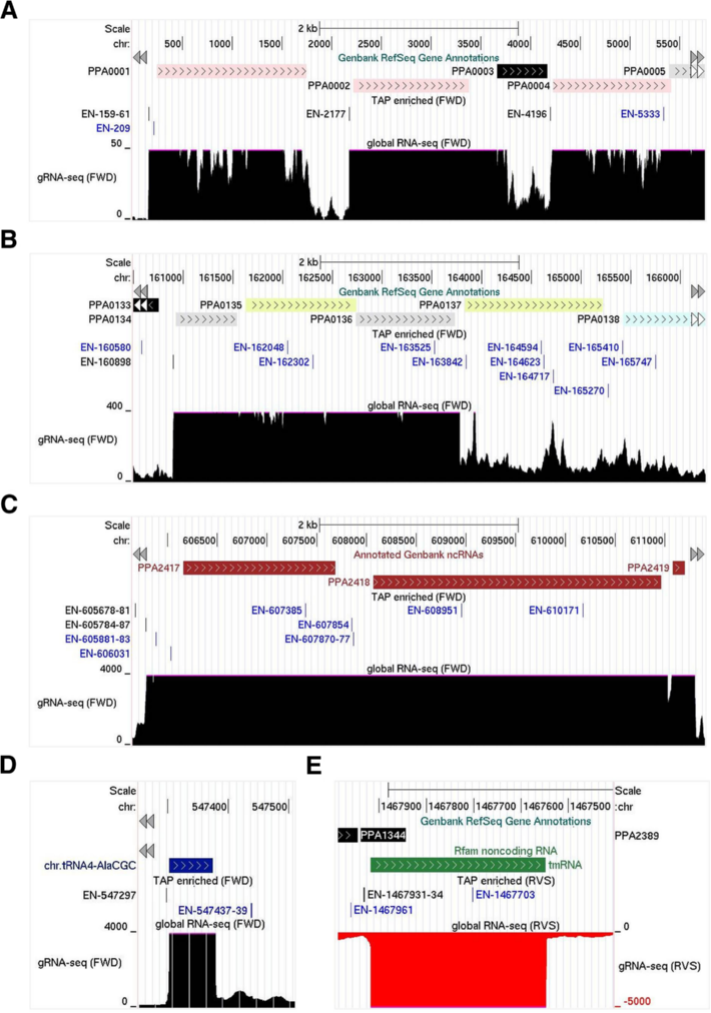
**Examples of TSSs associated with different classes of RNA.** All the corresponding genes are shown left to right regardless of whether they are encoded on the forward **(A)**, **(B)**, **(C)** and **(D)** or the reverse **(E)** strand. The global RNA-seq data for genes on the forward and reverse strand is coloured black and red, respectively. Furthermore, reads corresponding to the reverse strand have been given negative values. **(A)**, **(B)**, **(C)**, **(D)** and **(E)** show data corresponding to monocistronic mRNAs (PPA001, PPA002 and PPA004), polycistronic mRNA (PPA0134-6), ribosomal RNA (PPA2417-2419), transfer RNA (AlaCGC) and sRNA (tmRNA), respectively. The panels are screenshots from the UCSC Microbial Genome Browser [29]. In each panel the tracks depict from top to bottom, the position of annotated genes (protein or RNA coding, as appropriate), the positions of TSSs identified by the analysis of M-A scatterplots (Additional file 3), and the number of times each position on the corresponding strand was sequenced following fragmentation of the transcriptome (gRNA-seq). The numbers at the ends of the RNA-seq tracks indicate the scale of the sequencing reads, while the numbers at the top of each panel indicate the genome position. TSSs in black text were judged by viewing of the gRNA-seq data to be clearly associated with leading edges of transcription, while those in blue text were not. EN indicates that a TSS was *en*riched following TAP treatment.

Next, using the UCSC Genome Browser [27], we viewed the positions of the TSSs alongside genes annotated in GenBank [28] and transcription units revealed by gRNA-seq. With regard to the latter, it should be noted that for every position in the genome, we determined the number of times it was read irrespective of its position in fragmented RNA. Moreover, we limited the upper range of the gRNA-seq reads, which gives transcription units a block appearance, to make it easier to decipher their 5′ and 3′ boundaries. The main gene(s) of interest to us in any view are always depicted left to right. For genes on the reverse strand, the RNA-seq data are given negative values and are shown in red instead of black, and the genome position numbering is reversed. For all classes of functional RNA, mono- and poly-cistronic mRNAs, ribosomal RNAs, transfer RNAs and ubiquitous small RNAs, we were able to identify TSSs that aligned with the 5′ boundaries of transcription units revealed by gRNA-seq (for examples, see Figure 2). Such TSSs are represented by black vertical lines. Interestingly, the majority of TSSs we identified did not correspond to the 5′ boundaries of obvious transcription units, or at least those of annotated genes (for several examples, see panel B). These TSSs are represented by blue vertical lines. The function of the corresponding transcripts is cryptic with a large proportion being found within coding regions on either the sense or antisense strand. Evidence for ‘pervasive’ transcription, a widespread phenomenon in eukaryotes [30,31], is emerging in bacteria [14,32-46]. Of the TSSs we identified, 1106 were associated with the 5′ boundaries of transcription units corresponding to annotated genes, as illustrated in Figure 2, or produced discrete RNAs of high abundance relative to flanking regions (see section below). The TSSs associated with step increases in transcription are identified within Additional file 3.

### Processing and degradation sites (PDSs)

**Figure 3:**
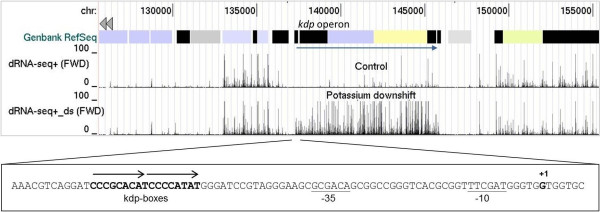
**Differential RNA-seq analysis of the *kdp *operon.** The tracks depict, from top to bottom, the positions of annotated genes in a condensed (packed) format, and the number of times each nucleotide position was the first in dRNA-seq reads (following TAP treatment) for samples taken following sub-culture without and with potassium downshift. The remainder of the labelling is as Figure 2. The inset shows the sequence in which the TSS was located. Black arrows indicate the position of direct repeats predicted to be the binding site of KdpE, the response regulator of the two component system, and underlining indicates the probable positions of the −35 and −10 boxes of the corresponding promoter (see text for details). The position of the TSS (+1) is marked.

The vast majority of the 5′ ends detected by dRNA-seq (~640,000) corresponded to processing and degradation sites (PDSs), which were not enriched following TAP treatment and were internal to transcription units identified by gRNA-seq. *P. acnes* encodes three endoribonucleases, RNase E [47], RNase Y [48] and RNase III [49], and a dual endonuclease/5′ to 3′ exonuclease, RNase J [50,51], (Additional file 4) that could account for the large number of PDSs. The coverage provided by PDSs is sufficient to allow the detection of transcription units and changes in gene expression. This is illustrated here using the *kdp* operon (Figure 3), which encodes an inducible high-affinity potassium transporter (Additional file 2) as well as an associated two-component sensory and regulatory system [21]. The vertical bars correspond to positions at which a 5′ end was detected by dRNA-seq following TAP treatment, irrespective of whether or not it was enriched. The bar height indicates the number of times a 5′ end was detected by sequencing. Shown are the results of analysing RNA extracted from cells with and without a potassium downshift. A horizontal arrow (in main panel) indicates the position of genes corresponding to the *kdp* operon. Following the downshift, it is clear from the dRNA-seq data that the expression across this entire operon increases substantial. The average number of reads increased by ~100 fold, which is similar to that detected using microarrays and gRNA-seq [52]. Genes with altered gene expression as a result of the downshift have been catalogued based on Rank product analysis of the microarray data (Additional file 5).

### Transcription and maturation of stable RNAs

In stark contrast to what has been found for *B. subtilis*[53], which along with *E. coli* is one of the main model systems in which tRNA processing has been studied in detail [11], none of the *P. acnes* tRNA genes (Additional file 6) are part of the three rRNA operons. Another striking difference is that most *P. acnes* tRNA genes are transcribed individually: we only found one example of a tricistronic tRNA operon (Val, GAC; Cys, GCA; Gly, GCC), and two examples of dicistronic operons (Met, CAT; Thr, GGT; and Asp, GTC; Phe, GAA). Thus, co-transcription does not appear to be a major means of regulating stable RNA production in *P. acnes*, unlike the situation in *B. subtilis*[53].

**Figure 4:**
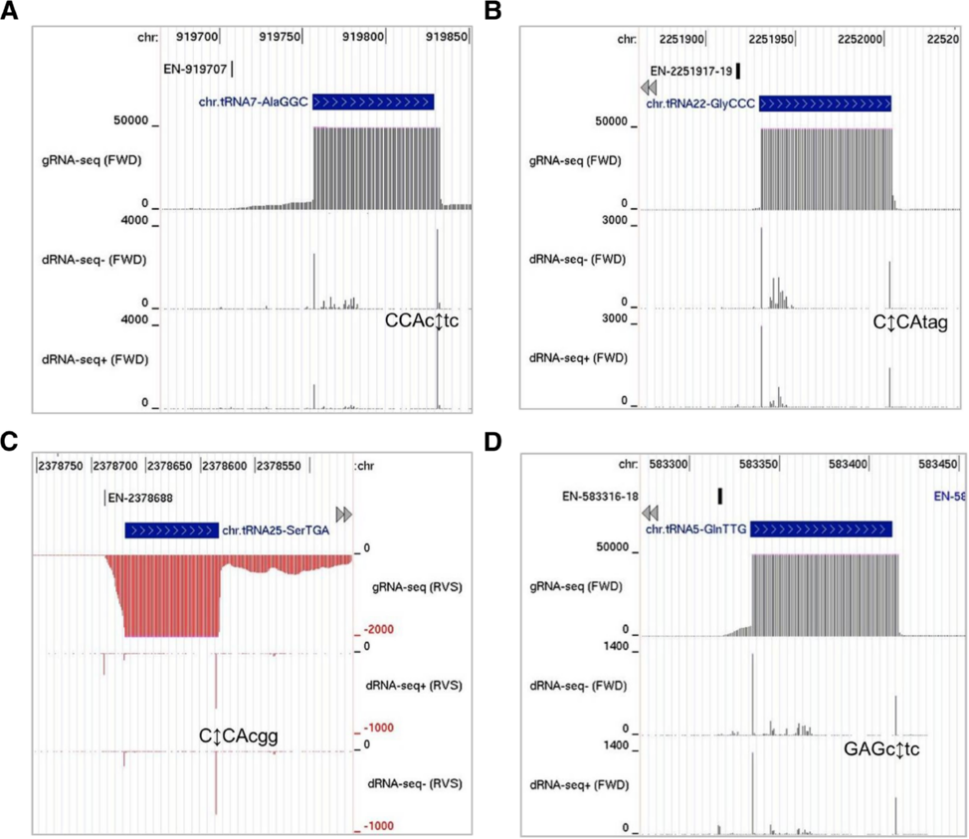
**Location of 3′ tRNA processing sites. (A)**, **(B)**, **(C)** and **(D)** correspond to tRNAs Ala (GGC, PPA2421), Gly (CCC, PPA2455), Ser (TGA, PPA2461) and Gln (TTG, PPA2415), respectively. As described for Figure 2, all the corresponding genes are shown left to right with the RNA-seq data for genes on the forward and reverse strands depicted by black, positive bars and red, negative bars, respectively. For each panel, the tracks depict, from top to bottom, the location of the TSS, the position of the tRNA gene, the gRNA-seq data, and the average of the dRNA-seq values before and after TAP treatment (combining values for the control and potassium-downshift sample). Vertical (double-headed) arrows indicate the peaks that correspond to processing on the 3′ side of the tRNAs. The sequences flanking these sites are also shown. The remainder of the labelling is as Figure 2. In **(A)** and **(B)**, the dRNA-seq reads corresponding to the TSSs are too low to be detected at the scales used to identify the processing sites.

Analysis of our dRNA-seq data revealed processing sites (*i.e.* 5′-end sequences of the downstream products of endonucleolytic cleavage) close to the 3′ ends of most *P. acnes* tRNAs (Figure 4), about half of which are encoded by genes with a 3′ CCA triplet (Additional file 6). As in the previous figure, all genes are drawn left to right irrespective of whether they are on the forward or reverse strand, and the RNA-seq data for genes on the forward and reverse strand are shown in black using positive values and in red using negative values, respectively. The dRNA-seq data incorporates an additional track showing the results obtained before TAP treatment. No increase in bar height following TAP treatment is an identifier of a site of processing or degradation. *P. acnes* has a homologue of tRNA nucleotidyltransferase (Additional file 4), which is known to attach CCA to tRNAs that are not transcribed with this motif [11]. A 3′ CCA on mature tRNA is essential for several functions related to translation [54]. Closer inspection of tRNAs encoded by genes with a 3′ CCA triplet revealed endonucleolytic cleavage a few nucleotides downstream of the tRNA (for an example, see panel A). This fits with current models in which the precursors of these tRNAs are trimmed back by 3′ exonucleases to produce a 3′ CCA end to which amino acids can be attached [11]. *P. acnes* contains homologues of RNase PH and RNase D, two 3′-5′ exonucleases known to be involved in trimming tRNA (Additional file 4). Interestingly, cleavage sites were also identified *within* 3′ CCA triplets (for examples, see panel B and C). In these examples, the cleavage was always between the Cs. To our knowledge such processing has not been described previously in bacteria. However, it is well established that at least some members of the tRNA nucleotidyltransferase family can recognise partial CCA ends and add only the residues that are missing [54]. Should *P. acnes* tRNA nucleotidyltransferase mediate this function *in vivo*, cleavages within 3′ CCA triplets would not result in terminal inactivation of the tRNA. The identity of the *P. acnes* endoribonuclease that cuts within 3′ CCA triplets is open to speculation. However, it is unlikely that it will be the homologue of RNase E as the *E. coli* enzyme has been found to have a preference for cutting bonds within sites rich in A and/or U nucleotides [55]. Whatever the actual identity of the endonuclease, it might also cut 3′ to tRNAs encoded by genes that lack a 3′ CCA triplet (Figure 4, panel D). *P. acnes* lacks an obvious homologue of tRNase Z, the endonuclease that generates the 3′ end for the post-transcriptional attachment of the CCA in *E. coli*, *B. subtilis* and other bacteria [11]. Differential RNA-seq also identified mature tRNA 5′ ends (Figure 4), which are generated ubiquitously by RNase P [11].

**Figure 5:**
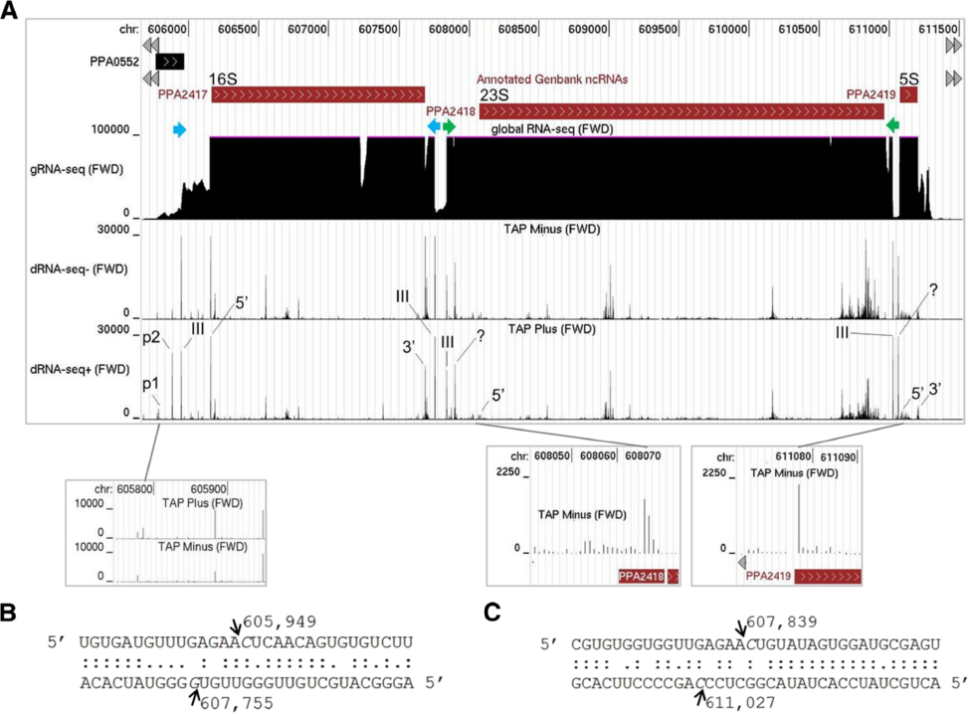
**Location of ribosomal RNA processing sites. (A)** shows an annotated view of the *rrnA* cluster. Labelling indicates the position of TSSs (p1 and p2) and major processing sites referred to in the text. The position of p1 is just downstream of a processing site associated with upstream transcription, which is detectable using a narrower range of reads. Short horizontal, filled arrows (blue and green) indicate the positions of complementary regions that facilitate extensive base-pairing. The insets show better the position of the promoters at the 5′ end of 16S RNA and cleavages at the very 5′ end of 23S and 5S using a narrower range of reads (following TAP treatment, dRNA-seq+). The remainder of the labelling is as Figure 2. **(B)** and **(C)** show the base-pairing of the complementary regions that flank 16S and 23S rRNA, respectively. For each, the positions of staggered cleavages are shown.

For each of the three rRNA operons in *P. acnes,* two TSSs were identified upstream of the 16S rRNA gene, an arrangement reported previously for *E. coli*[56-58] and *B. subtilis*[59]. For each operon, we also identified staggered cleavages in each of two pairs of complementary regions that flank 16S and 23S rRNA (Figure 5). These cleavages are likely mediated by the *P. acnes* homologue of RNase III (Additional file 4), which is a well-characterised, double-strand-specific endoribonuclease [60] with a widespread role in the maturation of ribosomal RNA [10]. In addition to sites of RNase III cleavage, we identified sites corresponding to the mature 5′ ends of all three ribosomal RNAs and the mature 3′ end of 16S rRNA. We also identified sites within one or two nucleotides downstream of the mature 3′ end of 23S and 5S rRNA. This indicates that, as found for other bacteria, the maturation of rRNA in *P. acnes* requires the action of multiple endoribonucleases. All of the sites described above were associated with a step change in transcript level as revealed by gRNA-seq (data not shown). Following endonucleolytic cutting, the mature 3′ ends of *P. acnes* 23S and 5S rRNA are likely produced by trimming of short 3′ tails. Regarding the generation of the mature 5′ end of 16S rRNA*, P. acnes* has homologues of both RNase J and RNase E (Additional file 4), ribonucleases that mediate this function in *B. subtilis* and *E. coli*, respectively [10]. Interestingly, we also identified multiple cleavage sites within 16S, 23S and 5S rRNA. These may represent steps in controlling the quality of rRNA (and ribosomes) [61], preventing rRNA accumulating in excess over ribosomal proteins [62,63] or simply mediating the normal turnover of the RNA. The nature of cleavages within processed rRNA is being investigated.

### Processing within mRNA

**Figure 6:**
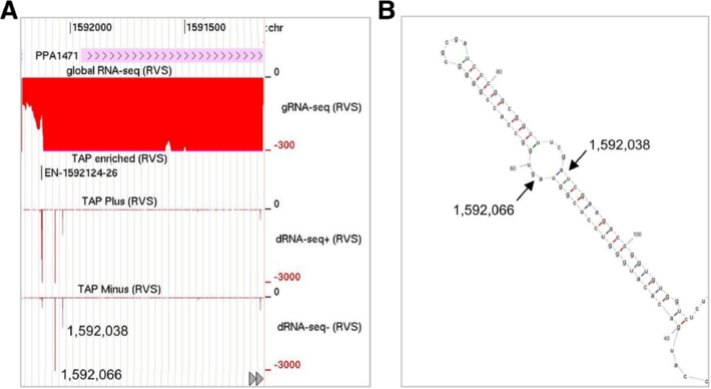
**Location of RNA processing sites in the 5′ UTR of *pnp *mRNA. (A)** shows the gRNA-seq and dRNA-seq data, while **(B)** shows the location of the processing sites relative to the secondary structure of the 5′ UTR as predicted using MFold [64]. The labelling of **(A)** is as Figure 2. The RNA-seq data for *pnp* is depicted by red negative bars as this gene is encoded on the reverse strand. In **(B)** the nucleotide positions are numbered relative to the TSS, while the sites of processing are numbered relative to the genome.

We were also able to identify specific cleavage sites in other classes of RNA. For example, we detected staggered cuts within a base-paired region of the 5′ leader of *pnp* mRNA (Figure 6), which encodes a 3′-5′ exonuclease (Additional file 4). Cleavage at the equivalent sites in *E. coli* by RNase III has been shown to produce 3′ ends that are accessible by PNPase. This facilitates an autoregulatory mechanism that ensures any overproduction of PNPase is only transitory as it leads to increased degradation of *pnp* mRNA [65-67]. This autoregulatory mechanism would appear to be evolutionarily conserved: experimental evidence for its existence in *Streptomyces* spp. has been obtained [68].

### Vegetative promoters

**Figure 7:**
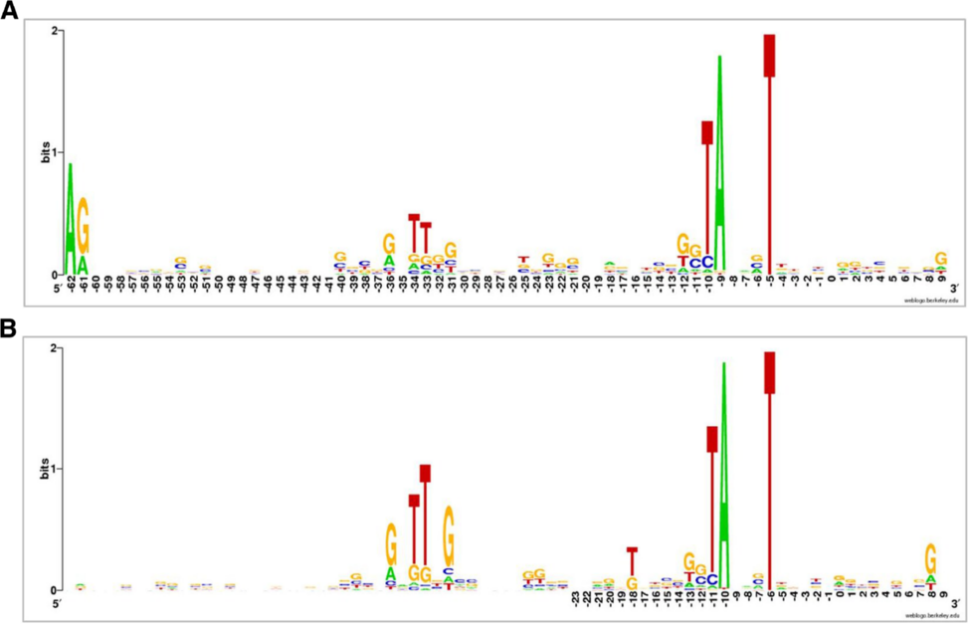
**Conserved sequences in promoters associated with genes encoding the translational machinery. (A)** and **(B)** are Weblog representations [70] without and with changing the length of the spacer of individual promoters to maximise alignment of the −35 box (Additional file 7). The combined height of nucleotide symbols shows the level of sequence conservation at a particular position, while the height of individual symbols within a stack of nucleotides indicates the relative frequency at that position. The nucleotide positions are numbered relative to the average position of TSSs. In **(B)** numbering only extends to the point at which gaps were introduced to maximise the alignment.

To gain knowledge of vegetative promoters in *P. acnes*, we aligned with the aid of MEME [69] the sequences upstream of 92 TSSs associated with genes of the translational machinery (Additional file 7). This revealed hexanucleotide sequences GnTTnG and TAnnnT centred on average −36 and −9 nt, respectively, from the TSSs (Figure 7). These sequences and their relative locations are similar to the consensus reported previously for ‘vegetative’ promoters of *E. coli*[71,72]. Following convention established for *E. coli*, we will refer to the above *P. acnes* sequences as ‘-35’ and ‘-10’ boxes, respectively. The consensus sequences of the equivalent boxes in *E. coli* promoters, TTGACA and TATAAT, are centred on average −33 and −10 nt, respectively, from the centre of the TSSs [71,72]. The positioning of the −35 box of *E. coli* closer to the TSS, means that the shared TnG (located in the 5′ half of the *E. coli* box and in the 3′ half of the *P. acnes* box) is on average in the same position relative to the TSS in both organisms. Thus, it appears that the sequence specificity of the housekeeping RNA polymerases in *P. acnes* and *E. coli* retain several elements in common despite these organisms diverging hundreds of millions of years ago.

We next analysed the sequences upstream of the 1106 TSSs associated with step increases in transcription, *i.e.* the 5′ boundaries of obvious transcription units. This revealed that the vast majority had appropriately positioned sequences matching the −10 box consensus. For example, using MEME, we identified 872 (79%) that matched the single most common sequence variant (TAnnnT). As an aside, this finding reinforces the fact that the combined RNA-seq approach described here identifies *bona fide* TSSs. Computational predictions of TSSs in *Propionibacterium* and related genera that utilise the promoters identified here as a learning set will be presented elsewhere. Consistent with the analysis of the promoters of rRNA, r-protein and tRNA genes (Additional file 7 and Figure 7), the overall level of sequence conservation at the −35 position was considerably lower. Nevertheless, promoter sequences were identified that also matched the single most common sequence variant of the −35 box consensus (GnTTnG). In addition to the promoters of rRNA, r-proteins and tRNA genes, this included the promoters of the genes of translation factors EF-Tu (PPA1873), IF-2 (PPA1493) and IF-3 (PPA1414) and central metabolism enzymes, *e.g.* alanine dehydrogenase (PPA2274), dihydrolipoamide acyltransferase (PPA0693), uridylate kinase (PPA1519), 3-oxoacyl-(acyl-carrier-protein) reductase (PPA1533), cytochrome d ubiquinol oxidase subunit I (PPA0176), fructose-1,6-bisphosphate aldolase (PPA2024), isopentenyl diphosphate delta isomerase (PPA2115), nitric-oxide reductase subunit B (PPA1975), and polynucleotide phosphorylase (PPA1471). Moreover, these promoters were associated with some of the highest transcript levels (data not shown), consistent with the well-established finding that promoters with matches to a consensus tend to be ‘strong’ [73].

### Uncovering multiple layers of regulation

**Figure 8:**
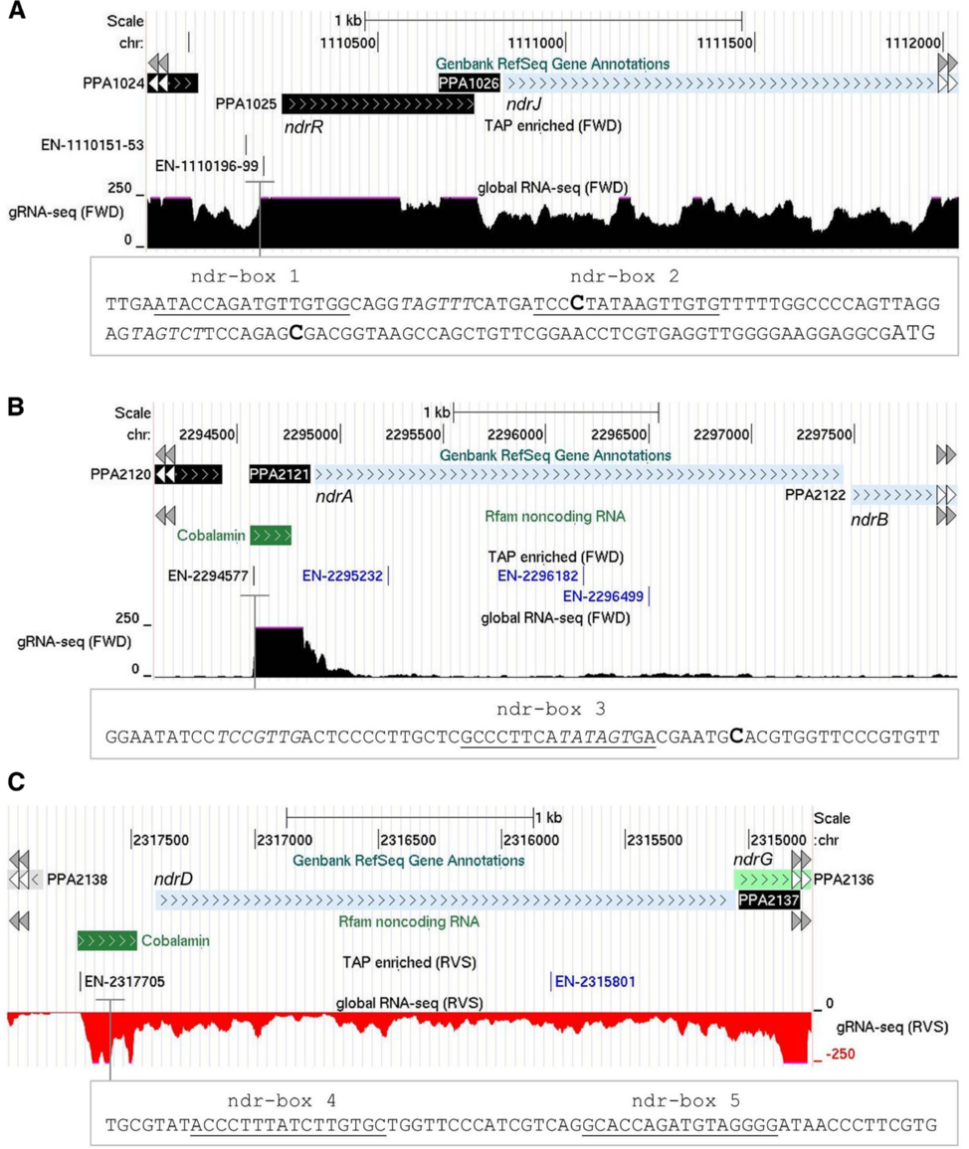
**Transcription, promoters and *cis*-regulatory motifs within the *nrd *operons. (A)**, **(B)**, and **(C)** correspond to the *nrdRJ* (PPA1025-1026), *nrdAB* (PPA2121-2122), and *nrdDG* (PPA2137-2136) operons, respectively. For each panel, the tracks show, from top to bottom, the positions of annotated genes, the position of TSSs (Additional file 3) and the gRNA-seq data. As the *nrdDG* operon is encoded on the reverse strand, the corresponding RNA-seq data is depicted by red negative bars. The inset in each panel shows the positions of predicted nrd-boxes and their sequences (underlined text) in relation to the TSS (bold, larger font text), where appropriate. The remainder of the labelling is as Figure 2.

The identification of TSSs and promoter sequences within the context of high-resolution transcriptome maps provides a much improved platform for assessing the complexity of gene regulation. This is illustrated here using the *P. acnes* homologue of NrdR, a transcription factor that controls the expression of ribonucleotide reductases (RNRs) [74,75], key enzymes that catalyse the formation of deoxyribonucleotides from ribonucleotides [76]. By using MEME to compare sequences positioned −60 to +15 relative to TSSs mapped for *nrdR* and genes encoding components of RNRs, we were able to identify probable binding sites for NrdR (herein referred to as nrd-boxes). These binding sites overlapped some, but not all of the identified promoters: a pair of nrd-boxes overlapping the distal of the two promoters for the *nrdRJ* operon and a single nrd-box overlapping the *nrdAB* promoter (Figure 8). Moreover, after constructing a position-weight matrix and scanning the entire genome of *P. acnes* using PREDetector [77], we identified another pair of nrd-boxes far downstream of the *nrdDG* promoter active under the growth conditions used in this study. Thus, it appears that the transcription of *nrdR* and several of its targets are under the control of multiple promoters, only some of which are regulated by NrdR.

We also found evidence of post-transcriptional control: much of the transcription of the *nrdAB* operon (encoding subunits of a RNR) appears to terminate before the first structural gene (Figure 8). Consistent with this interpretation, the 5′ UTR region of *nrdAB* is annotated as containing a cobalamin riboswitch [78], a *cis*-regulatory element that is widely distributed in the 5′ UTRs of cobalamin (vitamin B12)-related genes in bacteria [79-82]. Interestingly, the 5′ UTR of *nrdDG* (encoding an anaerobic RNR and activating protein), but not the *nrdRJ* operon is also annotated as containing a cobalamin riboswitch. Furthermore, the activity of the RNR encoded by *nrdJ*, which is co-transcribed with *nrdR*, is cobalamin dependent [83], and we detected expression of cobalamin biosynthetic genes (data not shown). The above suggests that cobalamin is present at sufficient levels to activate the riboswitches, which in down regulates the expression of *nrdAB* and *nrdDG*. The result is that much of the RNR production is via *nrdJ*.

Our results also lead us to propose that the NrdR repressor is active under the conditions used for this study. The bulk of the transcription of *nrdRJ* appears to initiate at the distal promoter, not the proximal promoter overlapped by a pair of nrd-boxes, and we did not detect transcription initiation in the immediate vicinity of the pair of nrd-boxes located upstream of *nrdDG*. We did detect relatively high levels of transcription from an *nrdAB* promoter, but this is overlapped by only a single nrd-box. We speculate that should a promoter exist in the vicinity of the nrd-boxes located upstream of *nrdDG* inactivation of NrdR will produce a transcript lacking a functional riboswitch, thereby removing the cobalamin regulation. The RNR encoded by the *nrdDG* operon is thought to function under anaerobic conditions.

### Comparison with standard 5′ RACE

**Figure 9:**
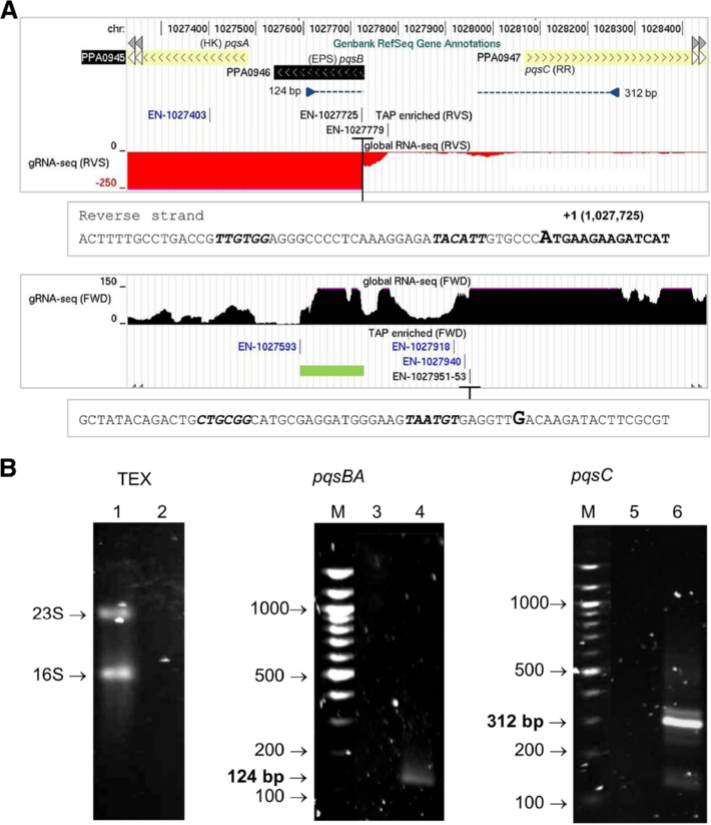
**RNA-seq analysis of the *pqs *locus.** In **(A)** the tracks are as detailed in Figure 8. For the insets below the gRNA-seq tracks, large bold font indicates TSSs identified by standard 5′ RACE **(B)**, and dRNA-seq (Additional file 3). The putative −10 and −35 boxes of the corresponding promoters are in italic font. The genes of the histidine kinase, response regulator extracellular and signalling peptide are PPA0945, PPA0947 and PPA0946, respectively. RNA-seq data for both strands within the *pqs* locus is shown: black positive values correspond to the forward strand, while red negative values correspond to the reverse strand. The green box marks the boundaries for a potential antisense RNA regulator of the PPA0945-0946 transcription unit. Blue arrowheads indicate the binding sites of the primers that were extended to produce cDNA for *pqsBA* and *pqsC* mRNA. The sequences of the corresponding primers were 5′ CCTTGGGTTGCGTATTCACAG and 5′ TGTGAGACCGTCCATTTCAG, respectively. The dashed lines indicate the sizes of the cDNAs that were produced, as determined by agarose gel electrophoresis **(B)** and sequencing of the product (data not shown). **(B)**, lanes 1 and 2 show total RNA before and after treatment with TEX, respectively; lanes 3 and 4 show the reaction products of 5′ RACE analysis of *pqsBA* mRNA without and with template, respectively; lanes 5 and 6, as 3 and 4, except *pqsC* mRNA was analysed. The marker (M) was a 100-bp ladder (BioLabs). For details of primers and conditions, see *Methods*.

Prior to undertaking RNA-seq analysis, we had initiated a study of a two-component system that is likely involved in *P. acnes* quorum sensing [84]. Unusually, the genes of the histidine kinase (*pqsA*, PPA0945) and response regulator (*pqsC*, PPA0947) are divergently transcribed, and *pqsA* is preceded by a gene (*pqsB* PPA9046) that is predicted to encode an extracellular signalling peptide (Figure 9, panel A). Analysis of the transcription of this locus using standard 5′ RACE revealed single promoters upstream of both *pqsB* and *pqsC* (Figure 9, panel B). RNA-seq revealed much more: it not only identified both of the TSSs identified by 5′ RACE (to the precise nucleotides), it identified a second TSS that was associated with a clear step increase in transcription upstream of *pqsB* and identified a potential antisense RNA (with an associated TSS, EN-1027593) overlapping the 5′ end of *pqsB* (Figure 9). Both of these new elements have now been incorporated into a continuing dissection of the *pqs* locus. Additional TSSs were also detected by RNA-seq upstream of *pqsC*; however, their contribution to the expression of this gene is more difficult to assess due to background transcription. The TSS of *pqsB* overlaps the start codon to produce a leaderless mRNA, many more examples of which are described below.

### Identification of potential sRNAs

**Figure 10:**
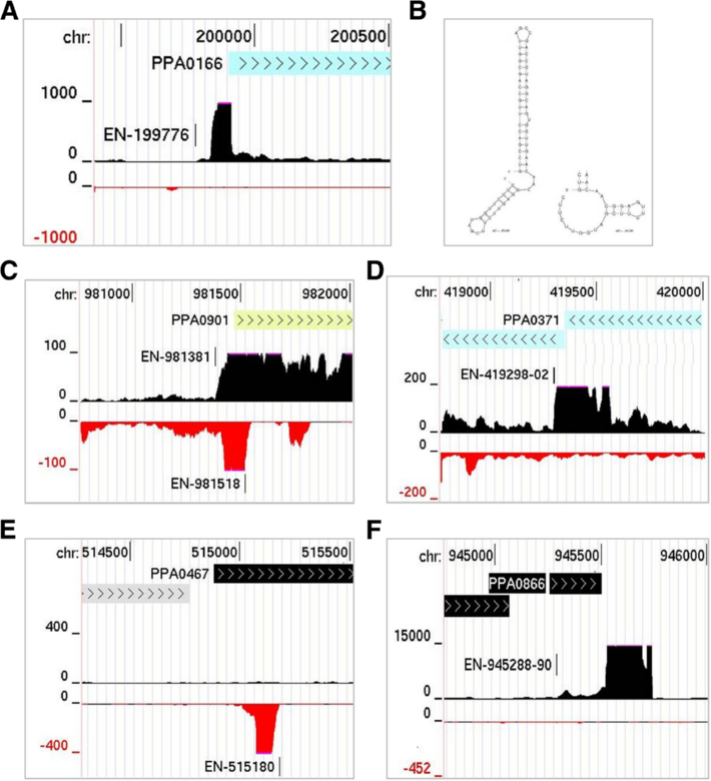
**Examples of *P. acnes *small RNAs. (A)** and **(B)** show the transcription and structure, respectively, of the 5′ UTR of PPA0166, which encodes a putative L-lactate permease. Alternative folding is shown bottom right of **(B)**. **(C)**, **(D)**, and **(E)** correspond to examples of potential antisense RNAs that bind 5′ RBSs, internal RBSs, and sites internal to the coding region, respectively. **(F)** shows an example of highly abundant sRNA that does not appear to overlap mRNA. For each of these panels, the tracks show the positions of annotated genes, the position of TSSs (Additional file 3) and the gRNA-seq data for both strands (black positive values correspond to the forward strand, while red negative values correspond to the reverse strand). The remainder of the labelling is as Figure 2.

As indicated above, we identified a number of TSSs that were associated with relatively short transcripts with high abundance relative to transcription in the surrounding regions (Additional file 8). These include all the ubiquitous bacterial sRNAs (6S RNA, tmRNA, the RNA component of RNase P, and Signal Recognition Particle RNA) [12] and 5′ UTRs with and without structural similarity to known riboswitches (*e.g.* TPP, FMN and Cobalamin) [85]. To be classified as a 5′ UTR, the peak of RNA abundance had to fall sharply at the boundary with the coding region of the downstream gene. Such a profile could be the result of riboswitching [85], attenuation-like mechanisms [86] or simply the higher stability (*i.e.* reduced susceptibility to the mRNA turnover machinery) that is often associated with structured regions [87]. An example of a 5′ UTR that has not, as far as we are aware, been linked with known riboswitches is shown in Figure 10 (panel A). Like all the potential 5′ UTRs that we have identified here, this is predicted to be structured (panel B). We also identified a few potential *cis*-acting antisense regulatory RNAs that are complementary to the ribosome-binding regions of overlapping mRNA and may thereby regulate the initiation of translation [12]. A complication is that many RNAs that are antisense to ribosome-binding regions could equally be the 5′ UTR of a divergent protein-coding gene. An example of a potential antisense RNA complementary to a 5′ ribosome-binding site (RBS) is shown (panel C), along with one that might interact with a RBS internal to a polycistronic mRNA (panel D). We also identified others that are complementary to sites distal to a RBS (for example, see panel E). It has recently been shown that antisense RNA binding to internal sites can initiate mRNA degradation via the stimulation of RNase E cleavage *in vitro*[88]. As mentioned previously, *P. acnes* has a homologue of this key endonuclease (Additional file 4). Small RNAs were also identified, some of which are highly abundant, that do not appear to overlap with protein-coding genes (for an example, see panel F). Our results indicate that, as is being found increasingly in other bacteria [12], sRNAs are likely to a significant role in regulating gene expression in *P. acnes*.

### Leaderless mRNAs

**Figure 11:**
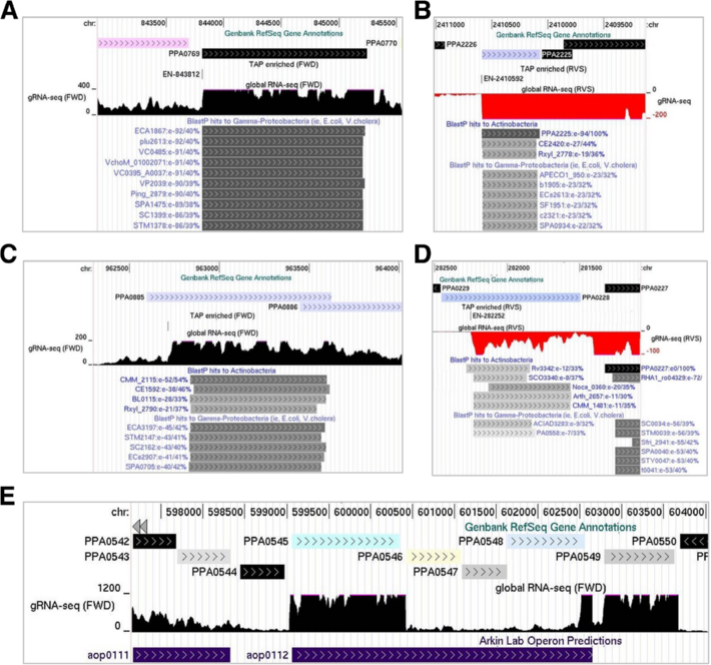
**Leaderless mRNAs and transcripts of genes requiring reannotation. (A)** and **(B)** correspond to examples of leaderless mRNA. PPA0769 encodes pyruvate kinase, and PPA2225 ferritin. **(C)** and **(D)** correspond to examples of genes requiring reannotation. PPA0885 encodes hydroxyethylthiazole kinase, and PPA0228 a putative methyltransferase. For each of these panels, the tracks show, from top to bottom, the positions of annotated genes, the position of TSSs (Additional file 3), gRNA-seq data, and BlastP hits or operon predictions. The remainder of labelling is as Figure 2. **(E)** shows the transcription data for *eno* (PPA0545), which encodes phosphopyruvate hydratase. Purple boxes indicate computational predictions of the operon structure in this region [94]. As in previous figures, all the genes are drawn left to right with their corresponding RNA-seq data (black positive values for forward strand, red negative values for the reverse strand).

While mapping TSSs, we noticed several examples that coincided with start codons for translation. This prompted us to gauge the prevalence of leaderless mRNAs in *P. acnes.* The start codons of protein-coding genes, as annotated in the NCBI database [89], were collated and their positions mapped against TSSs associated with a step increase in transcription (Additional file 3). This revealed 50 instances of annotated start codons overlapping TSSs and another 88 where start codons followed TSSs within 10 nt (Additional file 9). The latter TSSs produce a 5′ leader that is generally considered too short to recruit ribosomes via the canonical Shine-Dalgarno interaction, which requires base pairing between the leader and a complementary sequence in the 3′ end of 16S rRNA [90,91]. We also identified 15 instances of mRNAs with relatively short 5′ leaders (&20 nt) within which we were unable to detect a Shine-Dalgarno sequence using RBSfinder [92] (Additional file 9). From the above, we concluded that in sharp contrast to what has been found for nascent mRNAs in *E. coli*[93], translation initiation in the absence of a Shine-Dalgarno interaction appears to be prevalent in *P. acnes*. Analysis of the ontology of genes associated with ‘leaderless’ mRNA failed to identify enrichment of a particular function(s). This is in contrast to the situation in *E. coli* where repressors of mobile genetic elements are clearly enriched (Romero and McDowall, unpubl. results). Pyruvate kinase, a key glycolytic enzyme, and ferritin, the major store of iron, are two examples of products translated from leaderless mRNA in *P. acnes* (Figure 11, panels A & B). The proportions of AUG, GUG, UUG start codons present in *P. acnes* leaderless mRNA are 68%, 29% and 3%, respectively. This is similar to the proportion reported for another actinomycete, *Streptomyces coelicolor*[95]. To our knowledge, the mechanism by which leaderless mRNA is translated in actinomycetes has not been determined. Very recently it has been shown that leaderless mRNAs in *E. coli* can be generated post-transcriptionally by a stress-induced mRNase that is the toxic component of a toxin-antitoxin system [96]. Should such processing also exist in *P. acnes*, the detection of the corresponding sites would require us to add a phosphorylation step to facilitate the cloning and sequencing of the 5′-hydoxylated downstream products of cleavage by toxin mRNases.

### Re-annotation of protein-coding genes and operon structures

We also noticed that a significant proportion of TSSs associated with significant step increases in transcription were internal to the 5′ half of annotated genes (for examples, see Figure 11, panels C & D) suggesting that the actual gene might be shorter. Consistent with this notion, we have been able to find RBS along with appropriately spaced start codons downstream of many of these TSSs (Additional file 10) and homologous genes that lack sequences matching the 5′ end of the original gene annotation (panels C and D). Our combined RNA-seq approach also revealed many examples of operon structures that differ significantly from bioinformatics predictions (for example, see panel E). This was not particular surprising; it is known that even the best bioinformatic approaches are not completely accurate [97]. Nevertheless, achieving accurate information on gene and operon structures is essential for gene expression and regulation to be modelled [24] at the level of the whole cell [98]. Our transcriptome approach and data should hasten the achievement of this goal for *P. acnes*.

## Discussion

Here we describe a number of mechanistic insights gained from an improved dRNA-seq approach that distinguished sites of transcription initiation without erasing the secondary transcriptome using TEX, an enzyme that in our hands can degrade a substantial proportion of 5′-triphosphorylated RNA under the conditions recommended by the vendor (Additional file 11). With the advent of sequencing techniques that can provide in excess of 100 million reads (*e.g.* Illumina Solexa) there is now no need to erase the secondary transcriptome in order to detect transcription start sites. We simply used TAP [15] to distinguish tri- from mono-phosphorylated 5′ ends. This enzyme was used in earlier dRNA-seq approaches, but to facilitate the cloning of 5′-fragments remaining after TEX treatment [14,95]. Other improvements were to fragment the RNA after the addition of the 5′ adaptor to improve the efficient cloning of 5′ ends from large transcripts, and to combine with a gRNA-seq approach that does not require an amplification step [18]. The latter – used here for the first time to study bacterial transcription - allowed us to identify the 3′, as well as 5′, boundaries of transcripts. It should be particularly beneficial for the study of organisms with a high GC content in their genomes, as the amplification of segments of such genomes (or transcriptomes) is prone to PCR bias and artefacts [99]. Moreover, as we included biological replicates in our dRNA-seq approach and incorporated a statistical method that is ideally suited to the analysis of dRNA-seq data, we are confident that our analyses are not dominated by false positives. This is particularly relevant with regard to our finding that the majority of TSSs in *P. acnes* are not associated with step increases in transcription that continued into annotated genes or produced discrete RNAs of high abundance relative to flanking regions (Additional file 3).

Evidence for pervasive transcription on a genome scale has previously been obtained for several bacteria [14,32-46,100]. However, this has largely been in the form of the identification of transcripts antisense to annotated genes [35]. By including biological replicates and mapping TSSs in addition to transcripts, our study shows that pervasive transcription in *P. acnes* stems as much from transcription of the coding strand as the non-coding strand of annotated genes. Of the thousands of TSSs identified within annotated genes (and not associated with obvious transcription of a flanking gene), approximately half produced transcripts sense to the coding strand. The only strong bias we have identified so far is for the leading strand of replication (data not shown). Our interpretation is that clashes with oncoming DNA replication hinder pervasive transcription initiation on the lagging strand.

The initiation of pervasive transcription is not random. MEME analysis of sequences immediately upstream of TSSs associated with pervasive transcription revealed that a high proportion (61%) matched the single most common sequence variant (TAnnnT) of the −10 box of *P. acnes* vegetative promoters (data not shown). It is our view that much of the pervasive transcription observed in bacteria is a consequence of the ability of RNA polymerases to recognise a range of sequences, which means that these enzymes can initiate transcription from sub-optimal sites albeit at reduced frequency. Just because a region is transcribed (*i.e.* active) does not mean that it is has a function [101]. Nevertheless, pervasive transcription may be of evolutionary significance by facilitating the transcription of genes acquired horizontally and in turn the production of products that could confer a selective advantage. Pervasive transcription might also have been the source of *bona fide* sRNA regulation. Through spontaneous mutation of promoter regions upstream of TSSs or the acquisition of mobile elements such as REP sequences, which are known to stabilise transcripts [87], small RNAs could increase in abundance and their likelihood of being subject to selection.

Very recent work suggests that the folding architecture of the chromosome influences the location of pervasive transcription [102]. This is consistent with a model in which pervasive transcription is largely driven by the ability of RNA polymerases, given access, to initiate transcription from sub-optimal sites albeit at reduced frequency. Nevertheless, the growing evidence for pervasive transcription in bacteria challenges the dogma that transcription starts and ends just before and after a gene. Moreover, it seems likely that mechanisms exist to minimise the accumulation of pervasive transcripts. One means of controlling pervasive transcripts is likely to be RNA degradation. It is well established that RNA turnover is particularly rapid in the absence of protection by RNA-binding proteins or translating ribosomes and appropriately located structures [9,17,103,104]. In other words, the transcriptional landscape may be defined by the stability of bacterial transcripts more than is currently appreciated.

In addition to providing additional evidence for pervasive transcription, we have identified over a thousand TSSs and associated transcripts encompassing all classes of functional RNA, mono- and poly-cistronic mRNA, ribosomal RNA, transfer RNA, and ubiquitous small RNAs (Figure 2), This alone is an important milestone along the route to understanding the cellular workings of *P. acnes*. In addition, we have identified changes in gene expression resulting from potassium downshift (Figure 3), post-transcriptional steps (Figures 5 and 6) including unusual 3′ processing of tRNA (Figure 4), features of vegetative promoters (Figure 7), potential transcription factor-binding sites (Figures 8 and 9), functioning riboswitches as well as a number of sRNAs (Figure 10), and an abundance of leaderless mRNAs (Figure 11). We have also shown how knowledge of the above can be used to build models of gene regulation that inform experimental investigation (Figures 8 and 9). Simply knowing the position of a TSS narrows the search area for transcription factor-binding sites. For example, inspection of the promoter of the *kdp* operon identified a direct repeat overlapping the −35 region (Figure 3, inset), which is characteristic of the binding sites of the response regulators of two-component systems [105].

The prevalence of leaderless mRNAs in *P. acnes* is in stark contrast to the situation in *E. coli*, the main bacterial system in which the translation of leaderless mRNA has been studied [106,107]. Indeed, only two examples of *E. coli* leaderless mRNA have been widely reported, the *cI* repressor of bacteriophage lambda [108] and the *tetR* repressor of transposon Tn1721 [109]. The association between leaderless mRNA and repressors within mobile genetic elements in *E. coli* has been extended to the repressors of the Rac, e14 and Qin prophages by our own deep RNA-seq analysis of the *E. coli* transcriptome (unpubl. result). We speculate that some aspect of the translation of these leaderless mRNAs may be important in linking the mobilisation of the corresponding genetic elements and the physiological of their host. Recently, it has been shown that stress induces the production of specialised ribosomes that selectively translate a group of mRNAs made leaderless by MazF [96], an endoribonuclease of a toxin-antitoxin (TA) module. Like their mRNA targets, the specialised ribosomes are produced by MazF cleavage, which removes 43 nt from the 3′ end of *E. coli* 16S rRNA [96]. Intriguingly, we have mapped a processing site 53 nt from the 3′ end of *P. acnes* 16S rRNA (data not shown). This raises the possibility that specialised ribosomes, similar to those generated by MazF in *E. coli*, could mediate much of the translation in *P. acnes*. However, unlike the situation described for *E. coli*[110-112], the translation of leaderless mRNA in *P. acnes* does not appear to require that the start codon is AUG. As described above, a significant proportion of the leaderless mRNA in *P. acnes* have GUG (29%) or UUG (3%) start codons in place of AUG (68%) (Additional file 9).

Regardless of the actual mechanism by which leaderless mRNAs are translated, our study adds to a growing body of evidence that leaderless mRNAs are prevalent outside *E. coli* and its closest relatives and the notion that the mechanism of their translation may represent an ancient milestone in the evolution of gene expression [106,107]. A gene ontology analysis of leaderless mRNA in *P. acnes* revealed a wide distribution of cellular roles (data not shown). Thus, since the emergence of translation mediated by a Shine-Dalgarno interaction, there does not seem to have been divergence in terms of cellular functions that are dependent on leaderless translation in *P. acnes*. It will be interesting to establish for *P. acnes* whether there is a correlation between leaderless translation and the level of gene expression, as measured by protein levels, or the response of genes under conditions of stress or both.

We estimate that around 250 reads (gRNA-seq) correspond to 1 transcript per cell, assuming that the levels of RNase P and tmRNA are similar to those in *E. coli*[113-116]. This appears to be reasonable; our estimate yields 60,000 ribosomes per cell, which is within the range reported for *E. coli*[117] and other bacteria, when applied to the 15 million reads for *P. acnes* 5S rRNA. Many of the mRNAs we identified were associated with less than 250 reads. This does mean necessarily that not every cell expresses the corresponding gene, only that it was expressed for a proportion of the *P. acnes* cell cycle. Moreover, many mRNAs will not need to be continuously present to ensure translation produces sufficient protein for the daughter cell at division, given that the doubling time is relatively long (~6 hours). We estimate that transcription associated with pervasive initiation corresponds on average to 0.06 transcripts per cell (15 reads). This relatively low level is perhaps consistent with a background of sporadic transcription.

## Conclusions

In summary, by combining the differential and global RNA-sequencing approaches used here to analyse *P. acnes*, it is clear that major step increases in both knowledge and understanding of gene organisation and regulation can be obtained for any bacterial species*,* which in turn would inform experimental investigation, as illustrated using the *nrdR* operon and *pqs* locus, as well as further computational approaches. With regard to the latter, our data can be mined further, for example, to identify *cis*-regulatory signals that control transcription termination, initiation from the promoters of genes with shared cellular function, RNA processing and degradation, and to predict the potential structures and targets of small RNAs. Our RNA-seq approach can also be used to identify and compare genes expressed under particular conditions. There is excellent agreement between the microarray and gRNA-seq data [52] and we are currently comparing the expression profiles obtained here for cells growth in liquid culture with those of cells grown as a biofilm (Lin and McDowall, unpublished data) as well as cells grown by others in a rich complex medium [13]. With continuing annotation, it should be possible eventually to computationally model the *P. acnes* cell and its interaction with the environment and animal hosts [98].

## Methods

### *P. acnes* and its cultivation in defined media

*Propionibacterium acnes* strain KPA171202 was obtained from Ulm University, Göttingen, Germany [8] and cultivated in an anaerobic workstation (MACS-MG-1000, Don Whitely Scientific) at 34°C under 80% [v/v] N_2_, 10% [v/v] CO_2_, and 10% [v/v] H_2_. All analyses were done using cells cultivated without shaking in 100 mL of modified Holland Synthetic Medium (HSM) [84,118] in a 250-mL Erlenmeyer flask. Inocula were prepared in two stages. First a single colony isolated from the surface of reinforced clostridial agar [119] was used to inoculate 10 mL of tryptone-yeast extract-glucose (TYG) broth [120] in a 30 mL plastic universal bottle. After growing to stationary phase, an aliquot was used to inoculate 100 mL of TYG broth to an OD_600_ of 0.2. The culture was then incubated to an OD_600_ of 1.0, after which cells were harvested by centrifugation (3,000 × *g* for 20 min) and washed by first resuspending in 10 mL of HSM (pre-warmed to 34°C) and then repeating the harvesting step. Finally, the cells were resuspended in 10 mL of pre-warmed HSM and an appropriate aliquot was used to inoculate 100 mL of pre-warmed HSM to an OD_600_ of 0.2. To study the effects of a potassium downshift (depletion from growth media), a 100 mL culture of *P. acnes* was prepared as above and grown to an OD_600_ of 1.0, after which the culture was separated into two equal halves and cells harvested as described above. One half was washed using standard HSM, then used to inoculate 100 mL of fresh HSM and reincubated. The other half was processed in the same way, except the HSM lacked potassium (KH_2_PO_4_ and K_2_HPO_4_). After 1 h of reincubation, 12.5 mL of stop solution (95% [v/v] ethanol; 5% [v/v] phenol) was added to inhibit cell metabolism [121], and the cells were harvested by centrifugation. When necessary, cell pellets were stored frozen at −80°C.

### Isolation of bacterial RNA

Cell pellets of *P. acnes* were resuspended in Kirby mix [122], 1 OD_600_ unit per 100 μL, and transferred to Lysing Matrix B tubes containing fine silica beads (MP Biomedical). Tubes were then placed in a high-speed benchtop homogenizer (Fastprep-24, MP Biomedical; set at 6.5 M/s). Cells were lysed by three cycles of homogenisation for 1 min with cooling between each cycle in an ice-water bath for 1 min. Lysates were extracted using an equal volume of acidic phenol: chloroform: isoamyl alcohol (50: 50: 1) and then chloroform: isoamyl alcohol (49: 1). Nucleic acid in the aqueous phase was precipitated by adding NaCl to 150 mM and 2.5 × volumes of 100% [v/v] ethanol, chilling at −20°C for 1 h, and then harvested by centrifugation (13,000 × *g* for 30 min at 4°C). Nucleic acid pellets were washed twice with 700 μL of 70% [v/v] ethanol, air dried for 5 min and resuspended in RNase-free water. To remove contaminating DNA, samples were treated with DNase I using conditions described by the vendor (Ambion) and extracted with phenol: chloroform as described above. The concentration and integrity of RNA samples were determined using a NanoDrop™ 1000 spectrophotometer (Thermo Fisher Scientific) and agarose gel electrophoresis [121], respectively.

### Transcriptome analyses

The differential RNA-seq data was generated by vertis Biotechnologie AG (Germany) as a service that included the construction of cDNA libraries before and after treatment with TAP (Additional file 1), sequencing of libraries using an Illumina HiSeq platform (single end, 50-bp read length), and the alignment of sequences to the genome (NCBI, accession number AE017283). It should be noted that the 5′-sequencing adaptor was ligated to transcripts prior to fragmentation, thereby allowing the 5′ ends of both long and short transcripts to be detected. RNA was fragmented using a Bioruptor® Next Gen UCD-300™ sonication system (Diagenode), then tailed at the 3′ end using poly(A) polymerase (New England BioLabs), copied into cDNA using M-MLV reverse transcriptase (RNase H minus, AffinityScript, Agilent) and an oligo-dT primer, amplified by PCR and fractioned using gel electrophoresis. Fragments of 250–500 bp were selected for Illumina sequencing. Reads were trimmed of 5′ adapter and poly(A) sequences and mapped using the CLC Genomics Workbench and standard settings. Prior to differential RNA-seq, samples were enriched for mRNA using *MICROBE*xpress™-Bacteria beads, as described by the manufacturer (Ambion).

Global transcriptome sequencing was performed by Dr Lira Mamanova (Welcome Trust, Sanger Centre, Cambridge, UK) using a published methodology [18] on samples that were enriched for mRNA. RNA sequences from the global analysis were processed in-house using Galaxy [123]. Adapter sequences were removed and reads trimmed for quality before being mapped to the genome using Bowtie 1.0 [124] with custom parameter: -l 28 for read1, -l 20 for read2, and -y -a --best --strata. Pairs of datasets were compared using M-A (ratio-intensity) scatterplots, where M is Log_2_ (reads plus/minus TAP treatment), and A is (log_2_ plus + log_2_ minus)/2. For each value of A, we calculated the average (μ) and standard deviation (σ) of M in a moving window of 5,000 pairs that were sorted in ascending order of A. Upper and lower envelopes were defined by the equation: μ ± Xσ, and positions outside the envelope recorded, as described previously [22,23]. The value of X was user defined (see text below for details). Microarray data was collected by Roche NimbleGen (Iceland) as a service using 4 × 72 k format arrays. RNA samples were analysed from duplicate cultures of *P. acnes* following subculture with and without potassium downshift.

### 5′ RACE and RT-PCR analysis

The mapping of the 5′ ends of specific transcripts was done using a 2nd generation 5′/3′ RACE kit as described by the vendor (Roche Applied Science), except an aliquot of each RNA sample was treated with Terminator™ 5′-Phosphate-Dependent Exonuclease (TEX), as described by the vendor (Epicentre® Biotechnologies), prior to starting the mapping protocol. The sequences of specific primers are indicated in the legends to relevant figures. Primers were designed with the assistance of Primer 3 software [125] and purchased from Eurogentec. For the Reverse-transcription polymerase chain reaction (RT-PCR), cDNA was synthesised using SuperScript® RT III (Invitrogen) with random hexamers (100 nM) and 200 ng of RNA template, the rest of the protocol was carried out as stated by manufacturer with no modifications. The PCR reaction was carried out using GoTaq® DNA polymerase (Promega) according to the vendor’s instruction using cDNA diluted with RNase-free water as the template.

### Availability of supporting data

Both raw and processed RNA-seq data has been submitted to NCBI Gene Expression Omnibus (http://www.ncbi.nlm.nih.gov/geo/). Accession: GSE46883 and GSE46810.

## Competing interests

The authors declare that they have no competing interests of a financial or non-financial nature.

## Authors’ contributions

KJM, Y-fL and DRA designed the transcriptome study. KJM and Y-fL performed the bulk of the RNA-seq analysis, DRA provided valuable input. SG undertook the 5′ RACE analysis. LM performed the global RNA-sequencing. KJM and Y-fL wrote the paper. All authors read and approved the final manuscript.

## Supplementary Material

Additional file 1 **Schematic illustration of combined differential and global RNA-seq approach.** RNA samples were enriched for mRNA by depleting 16S and 23S rRNA. To differentiate 5′-triphosphorylated ends (three yellow, filled circles) generated by transcription from 5′-monophosphorylated ends (single yellow, filled circles) produced by RNA processing or degradation, an aliquot was incubated with tobacco acid pyrophosphatase (TAP; branch on left), which leaves a monophosphate on 5′ ends that were originally triphosphorylated. As a control, another aliquot was incubated under the same conditions, but without TAP (branch in centre). Both aliquots of this pair were then incubated separately with an adapter (red bar) that is only able to ligate to 5′-monophosphorylated ends. After terminating the 5′-end ligation reaction, the RNA was fragmented (not shown) to generate 3′ ends close to the native 5′ ends tagged with adaptor. The 3′ ends of the resulting fragments were then tailed using poly(A) polymerase (broken black bar). Fragmentation following the attachment of the 5′ adaptor allowed the efficient cloning of 5′ ends associated with long as well as short transcripts. To identify transcription units onto which 5′ ends could be mapped, a separate aliquot of the enriched mRNA was analysed using FRT-seq, an amplification-free form of strand-specific global RNA-seq (branch on right). To allow the mapping of all segments of transcripts, the RNA was fragmented and dephosphorylated prior to the ligation of adapter to 3′ ends (solid black bar). The RNA was then 5′ monophosphorylated to allow the ligation of a second adapter (red bar). The individual fragments were then reverse transcribed on the flow-cell without amplification, thus avoiding PCR biases and duplicates. The fragments were sequenced using an Illumina Genome Analyzer. Click here for file

Additional file 2 **Batch culture of *P. acnes *in Holland Synthetic Medium.** (A), the growth profiles were constructed from the results of replicate cultures (*n* = 13). The black and red plots correspond to growth following subculture without and with a potassium downshift, respectively. The error bars indicate the standard deviation of the OD_600_ readings. Times given are from the point of subculture. RNA was isolated from cultures 1 h after subculturing. There was no distinct exponential phase following the potassium downshift: cells grew without a discernible lag phase, but their growth rate appeared to decrease steadily with time. This may reflect the utilisation of phosphate reserves that were accumulated during growth on TYG. The doubling-time and specific growth rate during exponential growth in the absence of potassium downshift were 6.2 h and 0.111 h^-1^, respectively. (B), RT-PCR analysis of RNA isolated with and without potassium downshift: cDNA was synthesised from equal amount of *P. acnes* RNA, and used as template for PCR amplification of segments of the target genes PPA0010 (*gyrA*) and PPA0116 (*kdpB*) and analysed by electrophoresis using a 2.0% [w/v] agarose gel. Lane M shows a 100-bp DNA ladder (Fermentas), lanes 1 and 2 show PCR product using genomic DNA and no template, respectively. Lanes 3 and 4 show the PCR products of using cDNA synthesised from RNA isolated from cells subcultured without and with downshift, respectively. Lanes 5 and 6 are as lanes 3 and 4, but for a biological replicate. The sequences of the PCR primers were PPA0010F, 5′-CCCGTACTGGTCAGCGTTTA; PPA0010R, 5′-GCCGTCTGCTTGTACAGGTT; PPA0116F, 5′-CGGCAAGCAACTACTCATCA; and PPA0116R, 5′-TAAAGATGATCGCCGAGAGC. The *gyrA* transcript served as an internal control. Following potassium downshift, the *kdpB* gene is clearly induced. Click here for file

Additional file 3 **Transcriptional start sites identified for *P. acnes.*** All of the positions listed in this table were either enriched (EN) following treatment with TAP in all 4 of 4 experiments (see Figure 1) or associated with an obvious leading edge of transcription (LE) as judged by manual inspection of the global RNA-seq data or both. Nucleotide positions within 8 nt of each other were classified as belonging to the same TSS. The p-values are the probability that the number of reads corresponding to a 5′ end increase following treatment with TAP, *i.e.* are associated with a TSS, according to Rank product analysis [25,26]. Click here for file

Additional file 4 ***P. acnes *homologues of major factors involved in RNA processing and degradation.** Further details of factors involved in eubacterial RNA degradation and processing can be found in several recent reviews [9,10,16,104]. Click here for file

Additional file 5 **Genes with altered expression as a consequence of potassium downshift.** The microarray data obtained for each of the two duplicate cultures was analysed using M-A (ratio-intensity) scatterplots (data not shown). The vast majority of the points in each comparison (with vs. without downshift) were contained within boundaries described by the equation μ ± 3σ, where μ and σ are the average and standard deviation, respectively, of M in a moving window of 5,000 data-points sorted in ascending order of A [22,23]. The genes listed in this table were outside the boundaries in both of the two comparisons. The microarray data was also analysed using an online version of Rank Product algorithm [26], which detects differentially regulated genes in replicated microarray experiments. Overall the analysis of the M-A scatterplots appears to have been more sensitive. In two cases, it identified all of the genes in a cluster with related function, while Rank Product did not (see PPA1287-90 and PPA1758-60). It should be noted that genes linked to iron homeostasis were detected, *e.g.* genes encoding the ferrous iron transport proteins A and B (PPA1676 and PPA1677, respectively), and the production of a peptide-based iron chelators (PPA1287-1291). The potassium used to culture *P. acnes* was contaminated with trace amounts of iron. Thus, removing the potassium also removed a source of iron. P-values are for change in expression. ^+^Transcription starts within PPA0114. Click here for file

Additional file 6 **Processing of *P. acnes *tRNA.** n.d. = not detected (scale of 0–1000 dRNA-seq reads). Click here for file

Additional file 7 **Sequence alignment of *P. acnes *promoters associated with the translational machinery.** (A) shows ungapped sequences (+5 to −60) aligned to the ‘-10 box’ (consensus sequence of T.A.n.n.n.T), which was identified using MEME [69] and an initial search window of −1 to −15. (B) as (A), except gaps have been introduced 5 nt upstream of the −10 boxes to maximise alignment to the ‘-35 box’ (consensus sequence of G/A.n.T/G.T/G.n.G). Highlighting indicates nucleotides that match the consensus sequences (Figure 7). Click here for file

Additional file 8 **List of annotated and possible sRNAs in *P. acnes.*** Click here for file

Additional file 9 List of possible leaderless mRNAs. Click here for file

Additional file 10 List of genes requiring reannotation. Click here for file

Additional file 11 **Degradation of 5′-triphosphorylated RNA by TEX.** Total *P. acnes* RNA was isolated as described in *Methods*. A 5′-triphosphorylated form of *E. coli cspA* mRNA was synthesised by *in vitro* transcription using T7 RNA polymerase (Invitrogen) using conditions stated by the manufacturer [121]. 0.5 μg of *cspA* was added to 1.0 μg of total RNA and treated with TEX using Reaction Buffer B and 1 U of enzyme for 1 h as specified by the vendor (Epicentre® Biotechnologies). The reaction products were purified by phenol: chloroform extraction and analysed by gel electrophoresis (1.2% [w/v] agarose). Lane 1 shows the control sample before treatment. Lanes 2 and 3 shows the products following incubation without (reaction buffer only) and with TEX, respectively. Click here for file
