# Supplementary figures and images for: A combination of improved differential and global RNA-seq reveals pervasive transcription initiation and events in all stages of the life-cycle of functional RNAs in Propionibacterium acnes, a major contributor to wide-spread human disease
Source: BMC Genomics. 2013 Sep 14;14:620. doi: 10.1186/1471-2164-14-620 (PMC3848588; doi:10.1186/1471-2164-14-620)

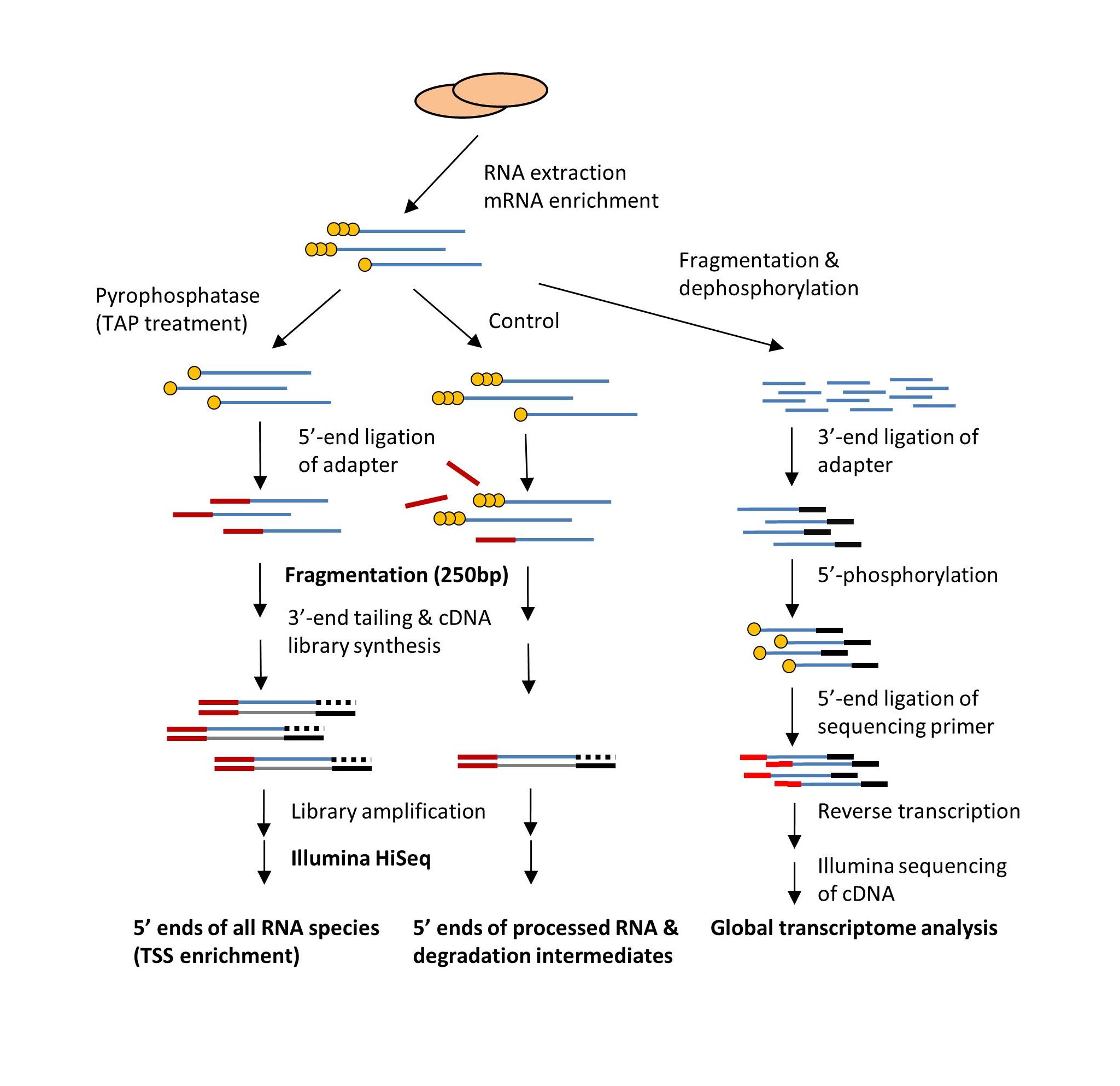

Supplement: Additional file 1 — Schematic illustration of combined differential and global RNA-seq approach. RNA samples were enriched for mRNA by depleting 16S and 23S rRNA. To differentiate 5′-triphosphorylated ends (three yellow, filled circles) generated by transcription from 5′-monophosphorylated ends (single yellow, filled circles) produced by RNA processing or degradation, an aliquot was incubated with tobacco acid pyrophosphatase (TAP; branch on left), which leaves a monophosphate on 5′ ends that were originally triphosphorylated. As a control, another aliquot was incubated under the same conditions, but without TAP (branch in centre). Both aliquots of this pair were then incubated separately with an adapter (red bar) that is only able to ligate to 5′-monophosphorylated ends. After terminating the 5′-end ligation reaction, the RNA was fragmented (not shown) to generate 3′ ends close to the native 5′ ends tagged with adaptor. The 3′ ends of the resulting fragments were then tailed using poly(A) polymerase (broken black bar). Fragmentation following the attachment of the 5′ adaptor allowed the efficient cloning of 5′ ends associated with long as well as short transcripts. To identify transcription units onto which 5′ ends could be mapped, a separate aliquot of the enriched mRNA was analysed using FRT-seq, an amplification-free form of strand-specific global RNA-seq (branch on right). To allow the mapping of all segments of transcripts, the RNA was fragmented and dephosphorylated prior to the ligation of adapter to 3′ ends (solid black bar). The RNA was then 5′ monophosphorylated to allow the ligation of a second adapter (red bar). The individual fragments were then reverse transcribed on the flow-cell without amplification, thus avoiding PCR biases and duplicates. The fragments were sequenced using an Illumina Genome Analyzer. [file 1471-2164-14-620-S1.jpeg]

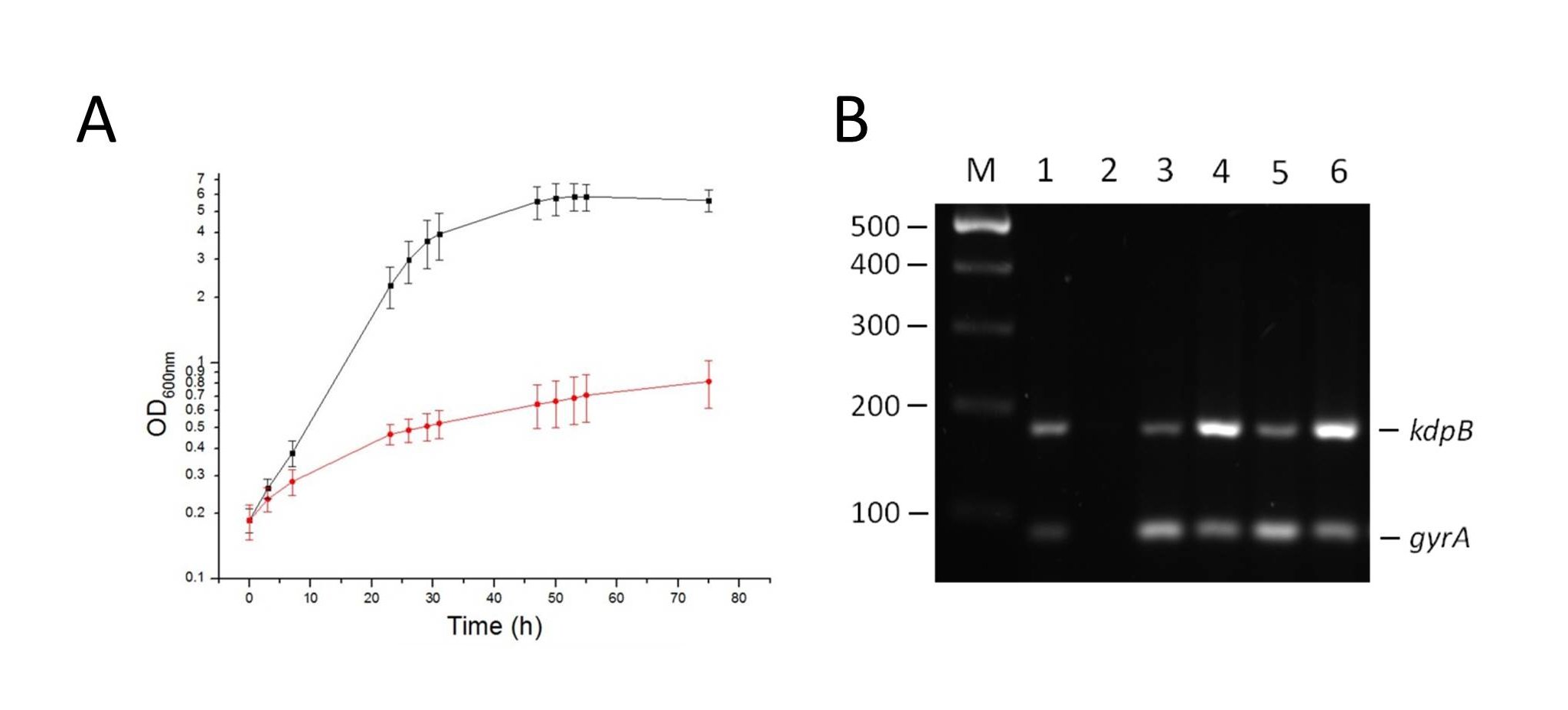

Supplement: Additional file 2 — Batch culture of P. acnes in Holland Synthetic Medium. (A), the growth profiles were constructed from the results of replicate cultures (n = 13). The black and red plots correspond to growth following subculture without and with a potassium downshift, respectively. The error bars indicate the standard deviation of the OD600 readings. Times given are from the point of subculture. RNA was isolated from cultures 1 h after subculturing. There was no distinct exponential phase following the potassium downshift: cells grew without a discernible lag phase, but their growth rate appeared to decrease steadily with time. This may reflect the utilisation of phosphate reserves that were accumulated during growth on TYG. The doubling-time and specific growth rate during exponential growth in the absence of potassium downshift were 6.2 h and 0.111 h-1, respectively. (B), RT-PCR analysis of RNA isolated with and without potassium downshift: cDNA was synthesised from equal amount of P. acnes RNA, and used as template for PCR amplification of segments of the target genes PPA0010 (gyrA) and PPA0116 (kdpB) and analysed by electrophoresis using a 2.0% [w/v] agarose gel. Lane M shows a 100-bp DNA ladder (Fermentas), lanes 1 and 2 show PCR product using genomic DNA and no template, respectively. Lanes 3 and 4 show the PCR products of using cDNA synthesised from RNA isolated from cells subcultured without and with downshift, respectively. Lanes 5 and 6 are as lanes 3 and 4, but for a biological replicate. The sequences of the PCR primers were PPA0010F, 5′-CCCGTACTGGTCAGCGTTTA; PPA0010R, 5′-GCCGTCTGCTTGTACAGGTT; PPA0116F, 5′-CGGCAAGCAACTACTCATCA; and PPA0116R, 5′-TAAAGATGATCGCCGAGAGC. The gyrA transcript served as an internal control. Following potassium downshift, the kdpB gene is clearly induced. [file 1471-2164-14-620-S2.jpeg]

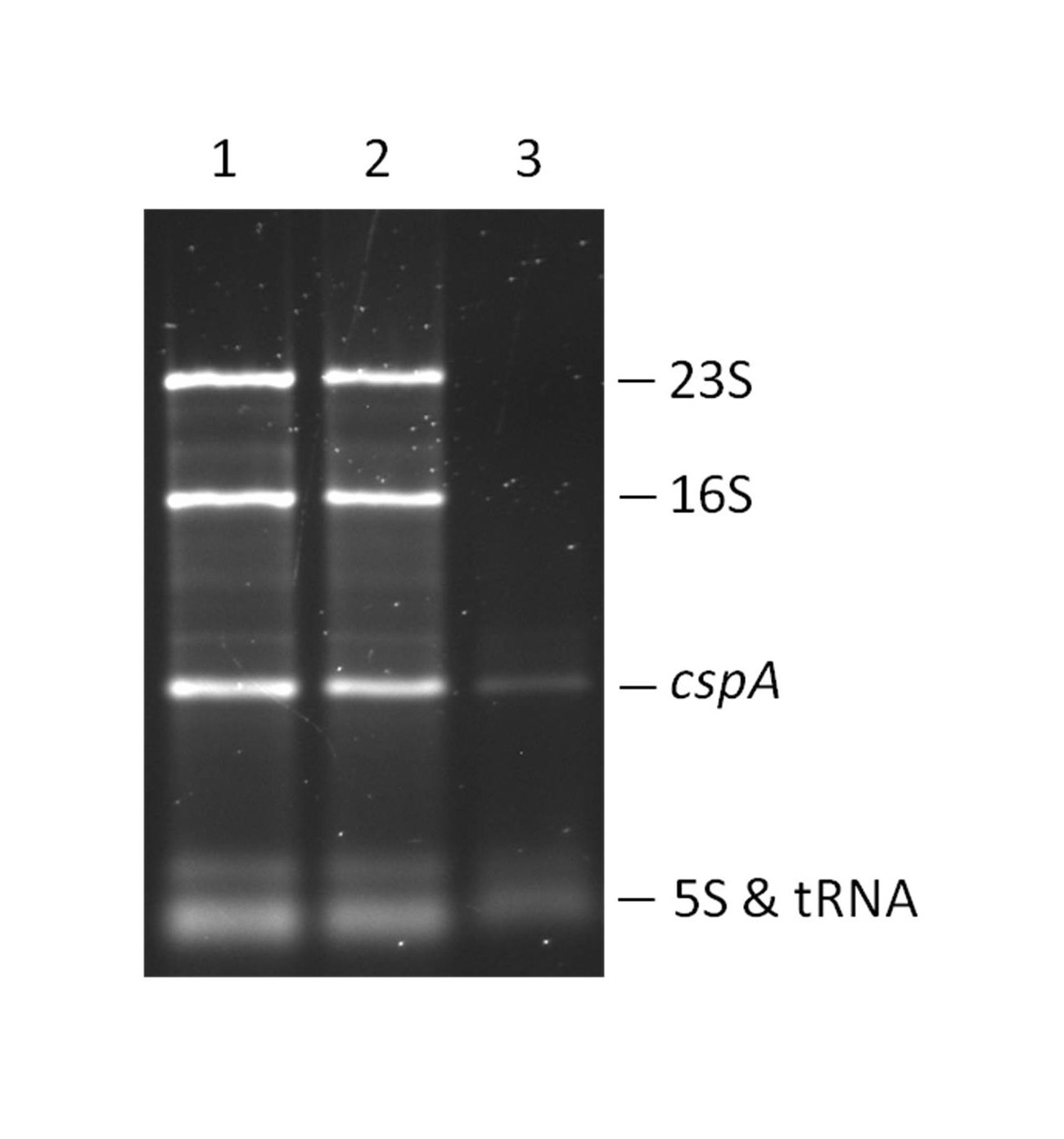

Supplement: Additional file 11 — Degradation of 5′-triphosphorylated RNA by TEX. Total P. acnes RNA was isolated as described in Methods. A 5′-triphosphorylated form of E. coli cspA mRNA was synthesised by in vitro transcription using T7 RNA polymerase (Invitrogen) using conditions stated by the manufacturer [121]. 0.5 μg of cspA was added to 1.0 μg of total RNA and treated with TEX using Reaction Buffer B and 1 U of enzyme for 1 h as specified by the vendor (Epicentre® Biotechnologies). The reaction products were purified by phenol: chloroform extraction and analysed by gel electrophoresis (1.2% [w/v] agarose). Lane 1 shows the control sample before treatment. Lanes 2 and 3 shows the products following incubation without (reaction buffer only) and with TEX, respectively. [file 1471-2164-14-620-S11.jpeg]
