# Supplementary material for: A combination of improved differential and global RNA-seq reveals pervasive transcription initiation and events in all stages of the life-cycle of functional RNAs in Propionibacterium acnes, a major contributor to wide-spread human disease
Source: BMC Genomics. 2013 Sep 14;14:620. doi: 10.1186/1471-2164-14-620 (PMC3848588; doi:10.1186/1471-2164-14-620)
Supplement: Additional file 3 — Transcriptional start sites identified for P. acnes. All of the positions listed in this table were either enriched (EN) following treatment with TAP in all 4 of 4 experiments (see Figure 1) or associated with an obvious leading edge of transcription (LE) as judged by manual inspection of the global RNA-seq data or both. Nucleotide positions within 8 nt of each other were classified as belonging to the same TSS. The p-values are the probability that the number of reads corresponding to a 5′ end increase following treatment with TAP, i.e. are associated with a TSS, according to Rank product analysis [25,26]. [file 1471-2164-14-620-S3.docx]

| **ID** | **Strand** | **LE** | **.p-value** |
| --- | --- | --- | --- |
| EN-62 | - | yes | 2.2E-07 |
| EN-159-61 | + | yes | 4.7E-08 |
| EN-209 | + | no | 5.5E-07 |
| EN-420 | - | no | 0.0E+00 |
| EN-537 | - | no | 2.5E-07 |
| EN-1094 | - | no | 1.1E-07 |
| EN-2177 | + | yes | 3.1E-08 |
| EN-4196 | + | yes | 0.0E+00 |
| EN-5333 | + | no | 2.9E-06 |
| EN-5707 | - | no | 9.2E-08 |
| EN-5920 | + | no | 6.5E-07 |
| EN-8304 | + | no | 2.4E-07 |
| EN-8554 | - | no | 0.0E+00 |
| EN-8649 | - | no | 1.5E-07 |
| LE-9642 | + | only | n/a |
| EN-10046 | - | no | 3.1E-08 |
| EN-12952 | - | yes | 1.3E-06 |
| EN-13781 | + | no | 1.6E-08 |
| EN-14466 | + | yes | 2.0E-07 |
| EN-15516 | - | no | 0.0E+00 |
| EN-15532 | - | no | 6.2E-08 |
| EN-15828 | + | no | 4.6E-06 |
| EN-16234 | - | no | 3.1E-08 |
| EN-16267 | + | no | 2.4E-07 |
| EN-16355 | - | yes | 7.7E-08 |
| EN-16648-51 | + | yes | 0.0E+00 |
| EN-17960-62 | + | no | 0.0E+00 |
| EN-21606 | - | no | 2.3E-07 |
| EN-22599-01 | + | yes | 1.1E-07 |
| EN-22813 | + | no | 0.0E+00 |
| EN-25506 | + | yes | 9.4E-08 |
| EN-25926 | + | yes | 4.9E-07 |
| EN-27990 | - | no | 1.2E-07 |
| EN-28525 | + | no | 1.1E-07 |
| EN-28606 | - | no | 6.2E-08 |
| EN-28683 | + | no | 4.2E-07 |
| EN-28725 | + | no | 1.1E-07 |
| EN-29312 | + | yes | 0.0E+00 |
| EN-29381-86 | + | yes | 0.0E+00 |
| EN-30585 | + | no | 0.0E+00 |
| EN-31009 | - | no | 0.0E+00 |
| EN-32561 | + | no | 0.0E+00 |
| EN-33124 | - | no | 0.0E+00 |
| EN-33167-69 | + | no | 2.5E-07 |
| EN-33231 | + | yes | 1.6E-08 |
| EN-33411-13 | - | yes | 1.1E-07 |
| EN-33505 | + | no | 2.4E-07 |
| EN-34769 | + | no | 5.0E-07 |
| EN-35652 | + | no | 3.1E-08 |
| EN-36009 | + | no | 0.0E+00 |
| EN-36053 | + | no | 1.1E-07 |
| EN-37908 | - | no | 0.0E+00 |
| EN-38376 | - | yes | 1.5E-08 |
| EN-39355 | - | no | 1.7E-07 |
| EN-42225 | - | no | 0.0E+00 |
| EN-42743 | - | no | 0.0E+00 |
| EN-43569 | - | no | 2.0E-07 |
| EN-46489 | - | no | 3.1E-08 |
| EN-47117-19 | - | no | 9.2E-08 |
| LE-47353 | + | only | n/a |
| EN-47438 | + | yes | 0.0E+00 |
| EN-48200 | - | yes | 6.9E-07 |
| EN-49259 | + | no | 3.1E-07 |
| EN-49466 | + | no | 9.4E-08 |
| EN-49893-95 | + | no | 1.1E-07 |
| LE-50161 | - | only | n/a |
| EN-50323 | - | no | 4.2E-07 |
| EN-52431 | - | no | 1.1E-07 |
| EN-52743 | - | no | 2.0E-07 |
| EN-53769 | + | no | 4.7E-08 |
| EN-54282 | + | yes | 0.0E+00 |
| EN-54660 | + | no | 0.0E+00 |
| EN-54739 | - | yes | 9.2E-08 |
| EN-54868 | - | no | 2.5E-07 |
| EN-55358 | - | no | 1.5E-07 |
| EN-55875-78 | + | no | 0.0E+00 |
| EN-56180 | - | no | 1.7E-07 |
| EN-56385 | - | no | 4.6E-08 |
| EN-58108 | + | no | 0.0E+00 |
| EN-58143 | - | no | 0.0E+00 |
| EN-58155 | + | no | 2.8E-07 |
| EN-58874 | + | no | 5.5E-07 |
| EN-59388 | + | yes | 0.0E+00 |
| EN-59508 | + | no | 5.5E-07 |
| EN-59649 | + | no | 1.4E-07 |
| EN-59751 | - | no | 6.2E-08 |
| EN-60879-82 | - | no | 4.6E-08 |
| EN-61043 | + | no | 1.1E-07 |
| EN-61104 | - | no | 6.2E-08 |
| EN-63821 | - | yes | 4.6E-08 |
| EN-64257 | + | no | 1.7E-07 |
| EN-64366 | - | no | 2.8E-07 |
| EN-67629 | - | no | 9.2E-08 |
| EN-69340 | + | yes | 0.0E+00 |
| EN-69922 | - | no | 1.2E-07 |
| EN-71692 | - | no | 2.3E-07 |
| LE-71843 | + | only | n/a |
| EN-73615 | - | no | 2.4E-06 |
| EN-74021 | - | yes | 6.8E-07 |
| EN-74204 | - | no | 9.2E-08 |
| EN-75511 | + | no | 7.9E-08 |
| EN-78802 | + | yes | 5.7E-07 |
| EN-78855-57 | + | yes | 4.5E-06 |
| EN-81325 | + | no | 6.6E-07 |
| EN-81851 | + | yes | 4.7E-08 |
| EN-83149-52 | + | no | 5.0E-07 |
| EN-84486 | + | no | 0.0E+00 |
| EN-84843 | + | no | 7.9E-08 |
| EN-84937 | + | no | 1.2E-06 |
| EN-84949 | + | no | 0.0E+00 |
| EN-85210 | - | no | 9.2E-08 |
| EN-85493 | + | yes | 2.5E-07 |
| EN-85563 | + | no | 0.0E+00 |
| EN-85745 | + | no | 8.0E-07 |
| EN-86981 | + | no | 0.0E+00 |
| EN-86995 | + | no | 4.7E-08 |
| EN-87907 | + | no | 7.9E-08 |
| EN-88283-85 | - | no | 1.5E-08 |
| EN-88291 | + | no | 5.5E-07 |
| EN-88847 | - | no | 7.8E-07 |
| EN-88870 | + | no | 1.1E-07 |
| EN-89167 | - | no | 1.7E-07 |
| EN-89764 | + | no | 1.6E-08 |
| EN-89797 | - | no | 1.1E-06 |
| EN-91291 | - | no | 9.2E-08 |
| EN-92414 | + | no | 7.9E-08 |
| EN-93233 | + | no | 0.0E+00 |
| EN-93513 | + | no | 2.0E-07 |
| EN-93653 | - | no | 0.0E+00 |
| EN-93668 | + | no | 9.8E-07 |
| EN-94011 | + | no | 1.6E-08 |
| EN-95550 | - | yes | 1.2E-05 |
| EN-95813-15 | + | yes | 2.0E-06 |
| EN-96333 | + | no | 1.6E-08 |
| EN-98122 | + | no | 0.0E+00 |
| EN-98496 | - | no | 1.5E-07 |
| LE-98741-45 | + | only | n/a |
| EN-98913 | - | no | 1.7E-07 |
| EN-99695 | + | no | 0.0E+00 |
| EN-101767 | + | yes | 0.0E+00 |
| EN-102654 | - | no | 1.2E-07 |
| EN-102830 | + | no | 2.8E-07 |
| EN-102866 | + | no | 0.0E+00 |
| EN-106998 | - | no | 0.0E+00 |
| EN-107171-74 | + | no | 2.4E-07 |
| EN-107414-17 | + | yes | 0.0E+00 |
| EN-107733 | - | no | 1.1E-07 |
| EN-108526 | - | no | 6.2E-08 |
| EN-108530-38 | + | yes | 3.1E-08 |
| EN-108605 | + | no | 4.4E-07 |
| EN-108630 | - | yes | 1.2E-07 |
| EN-108693-96 | + | yes | 0.0E+00 |
| EN-108777 | + | no | 0.0E+00 |
| EN-109406-09 | + | yes | 0.0E+00 |
| EN-109761 | - | no | 1.0E-06 |
| EN-111113 | + | no | 0.0E+00 |
| EN-111249 | + | no | 0.0E+00 |
| EN-112050 | + | no | 0.0E+00 |
| EN-112198 | + | no | 3.1E-06 |
| EN-112423 | - | yes | 0.0E+00 |
| EN-112723 | + | no | 0.0E+00 |
| EN-116691 | - | no | 1.8E-07 |
| EN-117728 | + | no | 1.1E-07 |
| EN-120167 | - | yes | 0.0E+00 |
| EN-121490 | + | no | 0.0E+00 |
| EN-122303 | + | no | 5.7E-07 |
| EN-123374 | + | no | 0.0E+00 |
| EN-126291 | + | no | 0.0E+00 |
| EN-128717 | + | no | 1.6E-08 |
| EN-128753 | + | no | 0.0E+00 |
| EN-129393 | + | no | 5.5E-07 |
| EN-129767 | + | no | 1.1E-07 |
| EN-130160 | - | yes | 0.0E+00 |
| EN-131118 | - | no | 3.1E-08 |
| EN-132188 | + | no | 1.6E-08 |
| EN-132386 | - | no | 0.0E+00 |
| LE-132888 | - | only | n/a |
| LE-133015 | + | only | n/a |
| EN-135024 | + | no | 5.5E-07 |
| EN-135024 | - | no | 0.0E+00 |
| EN-136299 | + | no | 7.1E-07 |
| LE-137320-22 | + | only | n/a |
| EN-139308 | + | no | 0.0E+00 |
| EN-139344 | + | no | 1.0E-06 |
| EN-141031 | - | no | 1.5E-08 |
| EN-141111 | + | no | 1.1E-07 |
| EN-141412 | + | no | 1.6E-08 |
| EN-141913 | + | no | 4.7E-08 |
| EN-143608 | + | no | 0.0E+00 |
| EN-143636 | - | no | 9.2E-08 |
| EN-145183 | + | yes | 0.0E+00 |
| EN-145449 | + | no | 9.4E-08 |
| EN-146259-61 | - | yes | 1.7E-07 |
| EN-148251 | + | yes | 1.6E-08 |
| EN-148754 | + | no | 2.5E-07 |
| EN-149028 | - | no | 7.7E-06 |
| LE-149245 | + | only | n/a |
| EN-149502 | + | no | 6.3E-07 |
| EN-150724 | - | no | 2.0E-07 |
| EN-150815 | + | no | 1.7E-07 |
| EN-151067 | + | no | 2.4E-07 |
| EN-151653 | - | no | 1.5E-08 |
| EN-152004 | + | no | 2.4E-07 |
| EN-152240 | + | no | 1.1E-07 |
| EN-152300 | + | no | 5.7E-07 |
| EN-153061-63 | + | no | 5.7E-07 |
| EN-153205 | + | no | 0.0E+00 |
| EN-153963 | + | no | 0.0E+00 |
| EN-155009 | + | no | 0.0E+00 |
| EN-156266 | + | no | 0.0E+00 |
| EN-157289 | - | yes | 2.7E-06 |
| EN-157527 | + | yes | 6.6E-05 |
| EN-158296 | - | no | 1.0E-06 |
| EN-158312 | - | no | 4.8E-07 |
| EN-158321 | + | no | 0.0E+00 |
| EN-159189 | - | no | 0.0E+00 |
| EN-159547-51 | + | no | 0.0E+00 |
| EN-159638 | - | no | 0.0E+00 |
| EN-160353 | - | no | 1.5E-08 |
| EN-160580 | + | no | 7.9E-08 |
| EN-160753 | - | yes | 1.6E-06 |
| EN-160898 | + | yes | 1.5E-05 |
| EN-161669 | - | no | 2.0E-07 |
| EN-162048 | + | no | 2.4E-07 |
| EN-162302 | + | no | 0.0E+00 |
| EN-163525 | + | no | 3.1E-08 |
| EN-163730 | - | no | 0.0E+00 |
| EN-163842 | + | no | 0.0E+00 |
| EN-163849 | - | no | 9.2E-08 |
| EN-163880 | - | yes | 4.6E-08 |
| EN-164594 | + | no | 0.0E+00 |
| EN-164623 | + | no | 0.0E+00 |
| EN-164717 | + | no | 4.9E-07 |
| EN-165270 | + | no | 2.8E-07 |
| EN-165410 | + | no | 0.0E+00 |
| EN-165747 | + | no | 0.0E+00 |
| EN-166907 | + | no | 4.7E-08 |
| LE-167222 | + | only | n/a |
| EN-167939 | - | no | 1.1E-07 |
| EN-169533 | - | no | 1.8E-07 |
| EN-169588 | - | yes | 4.6E-08 |
| EN-173374 | + | no | 7.1E-07 |
| EN-173741 | - | yes | 0.0E+00 |
| EN-173795 | + | yes | 1.5E-05 |
| EN-176004 | + | no | 6.0E-07 |
| EN-176189 | + | no | 1.6E-08 |
| EN-177527 | + | no | 1.1E-07 |
| EN-180704 | - | no | 3.5E-07 |
| EN-181711 | + | no | 4.7E-08 |
| EN-181862 | + | yes | 0.0E+00 |
| EN-183102-04 | - | yes | 2.2E-07 |
| LE-183102-07 | - | only | n/a |
| EN-183208 | + | no | 4.7E-08 |
| EN-183559 | - | no | 0.0E+00 |
| LE-186527 | + | only | n/a |
| EN-186778-80 | + | no | 0.0E+00 |
| EN-187192 | + | no | 0.0E+00 |
| EN-190597 | - | no | 2.0E-07 |
| EN-191114 | - | yes | 1.0E-06 |
| EN-195069 | + | no | 4.7E-08 |
| EN-196452 | + | no | 9.4E-08 |
| EN-196578 | + | no | 3.1E-08 |
| EN-198127 | + | no | 0.0E+00 |
| LE-198194 | - | only | n/a |
| EN-198407 | + | yes | 2.7E-07 |
| EN-199776 | + | yes | 7.3E-06 |
| EN-201366 | + | yes | 5.0E-07 |
| EN-201858 | - | no | 7.8E-07 |
| EN-201907-12 | - | yes | 0.0E+00 |
| EN-202812 | - | no | 4.2E-07 |
| EN-203181 | + | no | 2.8E-07 |
| EN-203708 | + | no | 0.0E+00 |
| EN-204789 | + | no | 3.3E-07 |
| EN-205381 | - | yes | 1.8E-07 |
| EN-205392 | + | no | 0.0E+00 |
| EN-206045-47 | + | no | 0.0E+00 |
| EN-206128-30 | + | no | 3.1E-08 |
| EN-206544 | - | no | 3.1E-08 |
| EN-206888 | + | no | 0.0E+00 |
| EN-206900-04 | + | yes | 0.0E+00 |
| EN-207376 | + | no | 1.1E-07 |
| EN-209217 | + | no | 4.7E-08 |
| EN-212318 | + | no | 2.4E-07 |
| EN-213052 | - | no | 6.2E-08 |
| EN-213202 | - | yes | 1.8E-06 |
| EN-215179 | - | no | 4.6E-08 |
| EN-215324 | - | no | 4.6E-08 |
| EN-215342 | + | no | 1.6E-08 |
| EN-215351 | + | no | 9.4E-08 |
| EN-215402 | - | no | 0.0E+00 |
| EN-215425 | + | no | 1.6E-06 |
| EN-215980-82 | + | no | 0.0E+00 |
| EN-216397 | - | no | 9.2E-08 |
| EN-216917 | + | yes | 0.0E+00 |
| EN-217717 | - | no | 6.2E-08 |
| LE-218796 | - | only | n/a |
| EN-218860 | + | yes | 4.4E-07 |
| EN-219005-08 | + | yes | 9.4E-08 |
| EN-219152 | + | no | 4.1E-07 |
| EN-219263 | + | no | 0.0E+00 |
| EN-220927 | + | no | 0.0E+00 |
| EN-221826 | + | no | 1.1E-07 |
| EN-222745 | - | no | 6.2E-08 |
| EN-222825 | + | no | 0.0E+00 |
| EN-222874 | - | yes | 6.6E-07 |
| EN-224719 | + | no | 4.2E-07 |
| EN-226605 | + | yes | 7.9E-08 |
| EN-227607 | - | no | 0.0E+00 |
| EN-227809 | - | no | 2.0E-07 |
| EN-228303 | - | no | 6.2E-08 |
| EN-230534 | - | no | 1.8E-07 |
| EN-231342-44 | - | yes | 1.2E-07 |
| EN-232499 | - | no | 0.0E+00 |
| EN-232509 | + | no | 3.6E-07 |
| EN-233541 | + | no | 1.6E-08 |
| EN-233555 | - | no | 0.0E+00 |
| EN-233633 | + | no | 2.4E-07 |
| EN-233667 | - | no | 0.0E+00 |
| EN-233886 | + | no | 1.6E-08 |
| EN-234105 | + | yes | 3.1E-08 |
| EN-235199 | + | no | 4.7E-08 |
| EN-235523 | - | no | 6.2E-08 |
| EN-235787 | + | yes | 0.0E+00 |
| EN-238359 | + | no | 0.0E+00 |
| EN-238386 | + | no | 1.6E-08 |
| EN-238431 | + | no | 0.0E+00 |
| EN-238639 | - | no | 7.7E-08 |
| EN-240032 | - | no | 1.5E-08 |
| EN-240090-92 | - | yes | 8.2E-07 |
| EN-240791 | - | no | 9.2E-08 |
| EN-241300 | + | yes | 3.6E-07 |
| EN-241486-89 | + | yes | 2.8E-07 |
| EN-241939 | + | no | 4.2E-07 |
| EN-243505 | + | no | 5.5E-08 |
| EN-244147-49 | + | no | 0.0E+00 |
| EN-245164-66 | + | no | 2.2E-07 |
| EN-246875-77 | - | yes | 6.2E-08 |
| EN-247034-37 | + | yes | 8.7E-06 |
| EN-247271 | - | no | 0.0E+00 |
| EN-247592 | + | no | 0.0E+00 |
| EN-247596 | - | no | 1.5E-08 |
| EN-247726 | + | no | 0.0E+00 |
| EN-247785 | - | no | 0.0E+00 |
| EN-248368 | - | no | 0.0E+00 |
| EN-248614 | - | no | 1.1E-07 |
| EN-249184 | - | no | 0.0E+00 |
| EN-250883 | + | no | 0.0E+00 |
| EN-251555 | - | yes | 2.0E-07 |
| LE-251811 | - | only | n/a |
| EN-252282 | - | yes | 2.8E-06 |
| EN-252385-87 | + | yes | 3.2E-06 |
| EN-252455 | + | no | 0.0E+00 |
| EN-252962 | + | yes | 4.4E-07 |
| EN-253334 | - | no | 1.6E-06 |
| EN-253638 | + | no | 1.1E-07 |
| EN-254512-14 | - | no | 1.5E-08 |
| EN-255896 | + | no | 1.1E-07 |
| EN-256011 | + | no | 0.0E+00 |
| EN-256488 | + | no | 2.4E-07 |
| EN-256572 | - | no | 4.6E-08 |
| EN-257094 | - | no | 6.2E-08 |
| EN-257182-85 | + | no | 9.1E-07 |
| EN-257755 | + | no | 3.6E-07 |
| EN-258531 | + | no | 1.1E-07 |
| EN-259272 | + | yes | 0.0E+00 |
| EN-259770 | + | no | 4.7E-08 |
| EN-260255 | + | no | 0.0E+00 |
| EN-260370 | - | yes | 1.6E-06 |
| EN-262310 | + | no | 0.0E+00 |
| EN-262668-70 | + | yes | 2.5E-06 |
| EN-262890 | - | yes | 2.9E-07 |
| EN-262964 | + | no | 4.7E-08 |
| EN-264693-95 | + | yes | 9.4E-07 |
| EN-264713 | + | no | 4.7E-08 |
| EN-265714 | - | no | 1.1E-07 |
| EN-265734 | + | no | 0.0E+00 |
| EN-266867 | + | no | 0.0E+00 |
| EN-267239 | - | no | 0.0E+00 |
| EN-267329 | + | no | 0.0E+00 |
| EN-267845-47 | + | yes | 1.1E-07 |
| EN-269032 | + | no | 7.9E-08 |
| EN-270791 | + | no | 9.4E-08 |
| EN-270921 | + | no | 0.0E+00 |
| EN-272376 | + | no | 2.4E-07 |
| EN-274234-40 | - | yes | 0.0E+00 |
| EN-275457 | - | no | 2.0E-07 |
| EN-275479 | + | no | 4.7E-08 |
| EN-275499 | + | yes | 0.0E+00 |
| EN-275563 | + | yes | 1.1E-07 |
| EN-275859 | + | no | 4.7E-08 |
| EN-276159 | + | no | 3.1E-07 |
| EN-277326 | - | yes | 0.0E+00 |
| EN-277396 | + | yes | 2.7E-07 |
| EN-278478 | + | no | 3.1E-08 |
| EN-279372 | - | no | 8.8E-07 |
| EN-279736 | - | no | 3.1E-08 |
| EN-279754 | + | no | 7.9E-08 |
| EN-279928 | - | no | 1.2E-06 |
| EN-279985 | - | no | 6.2E-08 |
| EN-279994 | - | yes | 4.2E-07 |
| EN-280003 | + | no | 3.1E-08 |
| EN-281142 | - | no | 0.0E+00 |
| EN-281407 | - | no | 4.6E-08 |
| EN-282252 | - | yes | 0.0E+00 |
| EN-285194 | + | no | 1.6E-08 |
| EN-285508 | - | no | 3.1E-06 |
| EN-287581 | - | yes | 0.0E+00 |
| EN-287826 | + | no | 0.0E+00 |
| EN-287978 | + | no | 1.0E-06 |
| EN-288138 | + | no | 0.0E+00 |
| EN-290135 | - | yes | 6.2E-08 |
| EN-290218 | - | no | 1.5E-08 |
| EN-291382 | - | no | 4.2E-07 |
| EN-292865 | - | no | 0.0E+00 |
| EN-293486 | - | no | 0.0E+00 |
| EN-293790 | - | no | 1.1E-07 |
| EN-294670 | - | no | 2.3E-07 |
| EN-296987 | + | no | 7.9E-08 |
| EN-297204 | + | no | 3.1E-07 |
| EN-298439 | + | no | 2.4E-05 |
| EN-298496 | + | yes | 0.0E+00 |
| EN-299156 | + | no | 2.7E-07 |
| EN-299168 | + | no | 2.4E-07 |
| EN-299963 | - | no | 0.0E+00 |
| EN-300050 | + | no | 7.9E-08 |
| EN-300305 | + | no | 0.0E+00 |
| EN-301858 | + | yes | 1.4E-06 |
| EN-303706 | + | no | 1.4E-06 |
| EN-303924 | - | no | 1.2E-06 |
| LE-304032 | + | only | n/a |
| EN-304139 | - | no | 9.2E-08 |
| EN-304263 | + | yes | 0.0E+00 |
| EN-304998 | - | no | 1.7E-07 |
| EN-305226 | - | no | 3.1E-07 |
| EN-305958-60 | + | no | 4.7E-08 |
| EN-306936 | + | yes | 9.4E-08 |
| EN-307514 | - | yes | 9.2E-08 |
| EN-307561 | + | no | 1.6E-08 |
| EN-308224 | - | no | 0.0E+00 |
| EN-309555 | + | no | 0.0E+00 |
| EN-309581 | - | no | 4.9E-07 |
| EN-310165 | + | yes | 0.0E+00 |
| EN-310280 | - | no | 2.0E-07 |
| EN-310296 | + | yes | 6.1E-07 |
| LE-310324 | - | only | n/a |
| EN-310331 | + | no | 0.0E+00 |
| EN-310405 | + | no | 3.1E-08 |
| EN-312570 | + | no | 1.1E-07 |
| EN-312708 | + | yes | 1.6E-08 |
| EN-313371-74 | + | yes | 1.6E-08 |
| EN-313378 | - | no | 6.2E-08 |
| EN-313407 | + | no | 9.4E-08 |
| EN-313725 | + | no | 1.7E-07 |
| EN-315951 | + | no | 0.0E+00 |
| EN-317525 | - | no | 1.5E-08 |
| EN-317703 | - | no | 9.2E-08 |
| EN-318765 | + | no | 0.0E+00 |
| EN-320261-64 | + | yes | 9.4E-08 |
| EN-320603 | + | no | 0.0E+00 |
| EN-320857 | + | no | 0.0E+00 |
| EN-321129 | + | no | 2.2E-07 |
| EN-321507 | - | no | 0.0E+00 |
| EN-322498 | + | no | 3.1E-06 |
| EN-322741 | + | no | 2.4E-07 |
| EN-323389 | + | no | 1.6E-08 |
| EN-323416 | + | no | 0.0E+00 |
| EN-323564 | - | no | 1.2E-05 |
| EN-323573 | + | no | 2.7E-07 |
| EN-323779 | + | no | 0.0E+00 |
| EN-323911 | + | no | 2.0E-07 |
| EN-324020 | + | no | 4.7E-08 |
| EN-324041-43 | + | yes | 3.0E-06 |
| EN-324931 | - | no | 0.0E+00 |
| EN-325091 | + | no | 0.0E+00 |
| EN-325431 | + | no | 4.4E-07 |
| EN-326067-70 | + | yes | 7.6E-06 |
| EN-326429 | - | yes | 1.8E-07 |
| EN-326575-78 | + | yes | 3.3E-07 |
| EN-327211 | - | no | 5.5E-06 |
| EN-327308 | - | no | 0.0E+00 |
| EN-328744 | - | no | 9.2E-08 |
| EN-331253 | - | no | 4.6E-08 |
| EN-332153 | + | yes | 3.1E-08 |
| EN-332582 | - | no | 0.0E+00 |
| EN-333106 | - | no | 2.7E-06 |
| EN-334428 | - | no | 9.2E-08 |
| EN-334537 | + | no | 9.4E-08 |
| EN-335107 | + | no | 3.1E-08 |
| EN-336125 | + | no | 2.4E-07 |
| EN-336475-78 | + | yes | 1.6E-08 |
| EN-337023 | - | no | 2.0E-06 |
| EN-337879 | - | no | 4.5E-06 |
| EN-337893 | + | no | 0.0E+00 |
| EN-338548 | - | no | 0.0E+00 |
| EN-338577 | - | no | 3.1E-08 |
| EN-339238 | + | no | 0.0E+00 |
| EN-339255 | + | no | 7.7E-07 |
| EN-344395-97 | + | yes | 1.1E-07 |
| EN-344821 | - | no | 0.0E+00 |
| EN-345997 | - | no | 0.0E+00 |
| EN-348407 | + | no | 3.3E-07 |
| EN-350102 | - | no | 1.5E-08 |
| EN-350182 | - | no | 0.0E+00 |
| EN-350629 | + | yes | 4.7E-08 |
| EN-351284 | + | no | 3.1E-08 |
| EN-351416 | + | no | 0.0E+00 |
| EN-351814 | - | no | 0.0E+00 |
| EN-352565-68 | + | yes | 0.0E+00 |
| EN-352837 | - | no | 0.0E+00 |
| EN-355205-07 | - | no | 6.9E-07 |
| EN-355224 | + | no | 7.9E-08 |
| EN-355443-46 | - | yes | 3.1E-08 |
| EN-356230 | - | no | 2.0E-07 |
| EN-356364 | - | no | 1.5E-07 |
| EN-356802 | - | no | 1.2E-07 |
| EN-357759 | - | yes | 1.3E-06 |
| EN-357871-73 | + | yes | 2.0E-07 |
| EN-358127 | + | no | 7.9E-08 |
| EN-359761 | - | no | 2.3E-07 |
| EN-359864 | + | no | 0.0E+00 |
| EN-360729 | - | no | 9.2E-08 |
| EN-361156 | + | no | 0.0E+00 |
| EN-361917 | + | no | 2.4E-07 |
| EN-362975 | + | no | 0.0E+00 |
| EN-363108-10 | - | no | 4.4E-06 |
| EN-363640 | - | no | 1.5E-08 |
| EN-364560 | - | no | 3.0E-06 |
| EN-364578 | + | no | 0.0E+00 |
| EN-365993 | - | no | 0.0E+00 |
| EN-366295 | - | no | 1.0E-05 |
| EN-366655 | + | no | 7.9E-08 |
| EN-366974 | - | yes | 8.8E-07 |
| EN-367055 | + | no | 2.8E-07 |
| EN-367125 | + | no | 1.1E-07 |
| EN-367220-22 | + | no | 4.7E-08 |
| EN-367517 | + | no | 2.4E-07 |
| EN-367713 | + | no | 0.0E+00 |
| EN-367771-74 | + | yes | 1.6E-08 |
| EN-369861 | + | yes | 2.7E-07 |
| EN-371387 | + | no | 0.0E+00 |
| EN-371738-41 | + | yes | 1.1E-07 |
| EN-372174 | - | no | 6.2E-08 |
| EN-372347 | + | no | 2.0E-07 |
| EN-373354 | - | no | 0.0E+00 |
| EN-374149 | + | no | 0.0E+00 |
| EN-374572 | + | yes | 0.0E+00 |
| EN-375627-30 | + | no | 0.0E+00 |
| EN-377061 | - | no | 1.8E-06 |
| EN-377749 | + | no | 5.0E-07 |
| EN-378261 | - | no | 1.5E-07 |
| EN-379207 | - | yes | 1.2E-07 |
| EN-379600 | + | no | 0.0E+00 |
| EN-380730 | + | no | 7.1E-07 |
| EN-380907 | - | yes | 1.1E-06 |
| EN-382217 | + | yes | 7.9E-08 |
| EN-383139 | - | no | 4.6E-08 |
| EN-383235 | + | yes | 3.3E-07 |
| EN-384370 | + | no | 0.0E+00 |
| EN-386870 | - | no | 3.1E-08 |
| EN-387710 | - | yes | 0.0E+00 |
| EN-389307-10 | + | no | 1.6E-08 |
| EN-389332 | - | yes | 3.1E-08 |
| EN-389922 | - | no | 7.8E-07 |
| EN-390219 | - | no | 9.2E-08 |
| EN-390318 | - | no | 0.0E+00 |
| EN-391526 | + | no | 1.1E-07 |
| EN-392074 | + | no | 1.6E-08 |
| EN-393050 | + | yes | 0.0E+00 |
| EN-394736 | + | no | 1.6E-08 |
| EN-396137 | - | no | 1.5E-08 |
| EN-396371 | - | no | 1.8E-07 |
| EN-396911 | - | yes | 9.2E-08 |
| EN-396972 | + | yes | 4.7E-08 |
| EN-398318 | - | yes | 6.8E-07 |
| EN-398357 | + | yes | 5.5E-07 |
| EN-399961-64 | + | no | 7.9E-07 |
| EN-400394 | + | no | 4.7E-08 |
| EN-401215 | - | no | 7.7E-08 |
| EN-401705 | - | no | 6.2E-08 |
| EN-403327 | - | no | 4.9E-07 |
| EN-404092 | - | no | 3.1E-08 |
| EN-404192 | - | yes | 1.1E-05 |
| EN-404258 | + | yes | 1.1E-07 |
| EN-404627 | - | no | 0.0E+00 |
| EN-404663 | + | no | 7.9E-08 |
| EN-405034 | + | no | 9.8E-07 |
| EN-405535 | - | no | 0.0E+00 |
| EN-405950 | - | no | 5.7E-07 |
| LE-405973 | + | only | n/a |
| EN-405984-86 | - | no | 0.0E+00 |
| LE-405994 | + | only | n/a |
| EN-406992-94 | - | yes | 4.6E-08 |
| EN-407190 | + | yes | 3.5E-05 |
| EN-407648 | + | no | 4.7E-08 |
| EN-407887 | - | no | 6.6E-07 |
| EN-408276 | + | no | 3.1E-08 |
| EN-409224 | + | no | 2.8E-07 |
| EN-409623 | - | yes | 1.2E-07 |
| EN-409768 | + | no | 0.0E+00 |
| EN-410401 | - | no | 0.0E+00 |
| EN-410420 | + | no | 7.6E-07 |
| EN-410555 | + | yes | 1.1E-05 |
| EN-411439-42 | + | no | 0.0E+00 |
| EN-412518 | - | yes | 2.0E-06 |
| EN-413035 | + | no | 1.1E-07 |
| EN-413735 | - | yes | 1.1E-07 |
| EN-414724 | - | no | 2.4E-07 |
| EN-415258 | - | no | 1.1E-07 |
| EN-415387 | - | no | 0.0E+00 |
| EN-415448-50 | - | no | 9.2E-08 |
| EN-415862 | - | no | 0.0E+00 |
| EN-416096 | - | no | 0.0E+00 |
| EN-416325 | - | no | 6.9E-07 |
| EN-416627 | + | no | 0.0E+00 |
| EN-417616 | + | yes | 0.0E+00 |
| EN-417979 | + | no | 2.4E-07 |
| EN-418168 | - | no | 0.0E+00 |
| EN-418713 | - | no | 6.2E-08 |
| EN-418732 | + | no | 1.7E-05 |
| EN-419298-02 | + | yes | 0.0E+00 |
| EN-420176 | + | yes | 0.0E+00 |
| EN-421347 | - | yes | 7.7E-08 |
| EN-423575 | + | no | 9.4E-08 |
| EN-426741-43 | + | no | 7.9E-08 |
| EN-428429 | - | yes | 2.3E-07 |
| EN-428468 | + | yes | 0.0E+00 |
| EN-429137 | - | yes | 6.2E-08 |
| LE-430775-80 | + | only | n/a |
| EN-430822 | + | no | 3.1E-08 |
| EN-431534 | + | no | 0.0E+00 |
| EN-432713-15 | - | yes | 7.8E-07 |
| EN-432731 | + | yes | 0.0E+00 |
| EN-432746 | + | no | 0.0E+00 |
| EN-432761 | + | no | 1.6E-08 |
| EN-434388 | - | yes | 0.0E+00 |
| EN-434625 | + | yes | 2.8E-07 |
| EN-436532 | - | no | 0.0E+00 |
| EN-437114 | + | no | 8.3E-07 |
| EN-437147 | - | no | 1.5E-07 |
| EN-437275-77 | + | no | 1.6E-08 |
| EN-437611 | - | no | 1.1E-07 |
| EN-437627 | + | no | 1.6E-08 |
| EN-438523 | + | no | 4.7E-08 |
| LE-439815 | - | only | n/a |
| EN-439836 | + | no | 0.0E+00 |
| EN-440309 | + | no | 1.6E-08 |
| EN-441336 | - | no | 1.1E-07 |
| EN-441870 | - | yes | 1.2E-07 |
| EN-442133 | + | no | 4.7E-08 |
| EN-442351 | + | yes | 0.0E+00 |
| EN-442382 | + | no | 0.0E+00 |
| EN-442781 | + | no | 7.9E-08 |
| EN-443846 | - | no | 0.0E+00 |
| EN-443976 | - | no | 0.0E+00 |
| EN-443987 | + | no | 0.0E+00 |
| EN-444147 | - | yes | 1.5E-07 |
| EN-445053 | + | no | 1.6E-08 |
| EN-446735 | + | no | 0.0E+00 |
| EN-447829 | + | no | 1.1E-07 |
| EN-447861 | + | no | 4.7E-08 |
| EN-447936 | - | yes | 2.3E-07 |
| EN-447955 | + | yes | 0.0E+00 |
| EN-447963 | - | no | 0.0E+00 |
| EN-447988-90 | + | no | 0.0E+00 |
| EN-449577-79 | + | yes | 0.0E+00 |
| EN-449703 | - | no | 9.2E-08 |
| EN-449721 | + | no | 5.2E-07 |
| EN-449975 | + | no | 0.0E+00 |
| EN-450509 | + | no | 2.5E-07 |
| EN-451099 | + | no | 0.0E+00 |
| EN-451779 | + | yes | 0.0E+00 |
| EN-452255 | - | no | 1.5E-07 |
| EN-452509 | + | no | 1.6E-08 |
| EN-453463 | + | no | 0.0E+00 |
| EN-453470-72 | - | yes | 2.0E-07 |
| EN-453574 | + | yes | 5.2E-07 |
| EN-453589 | + | no | 9.4E-08 |
| EN-453945 | - | no | 3.1E-08 |
| EN-454223 | - | no | 0.0E+00 |
| EN-454327 | - | no | 1.7E-06 |
| EN-455308 | - | no | 9.2E-08 |
| EN-455388-91 | + | yes | 2.1E-05 |
| EN-455851 | + | no | 3.3E-07 |
| EN-456573 | + | no | 2.4E-07 |
| EN-458330 | - | yes | 2.0E-07 |
| LE-458331 | - | only | n/a |
| EN-458363 | - | no | 0.0E+00 |
| EN-458377 | + | no | 0.0E+00 |
| EN-458469 | + | no | 2.4E-06 |
| EN-459661 | + | no | 7.9E-08 |
| LE-460965 | - | only | n/a |
| EN-461019 | + | no | 2.7E-06 |
| EN-462695 | - | no | 1.5E-08 |
| EN-463106 | + | no | 9.4E-08 |
| EN-463710 | + | yes | 1.6E-08 |
| EN-465197 | - | no | 2.0E-07 |
| EN-465215 | + | no | 9.4E-08 |
| EN-466913 | + | no | 1.6E-08 |
| EN-469090 | - | yes | 7.8E-07 |
| EN-469618 | + | no | 2.4E-07 |
| EN-470451 | + | yes | 0.0E+00 |
| EN-470655 | + | no | 1.1E-07 |
| EN-470748 | + | yes | 0.0E+00 |
| EN-472914 | - | no | 0.0E+00 |
| EN-473367 | + | no | 0.0E+00 |
| EN-473559 | - | no | 4.6E-08 |
| EN-474028 | - | no | 0.0E+00 |
| EN-475186 | - | no | 9.2E-08 |
| EN-475973 | - | no | 0.0E+00 |
| EN-476077 | + | yes | 3.1E-08 |
| EN-477216 | + | yes | 7.9E-08 |
| EN-477592 | + | no | 0.0E+00 |
| EN-479084 | + | yes | 9.5E-05 |
| EN-480388 | - | yes | 6.2E-08 |
| EN-480479 | + | yes | 0.0E+00 |
| EN-483207 | - | no | 1.7E-07 |
| EN-483471 | - | no | 1.5E-07 |
| EN-483937 | + | no | 9.4E-08 |
| EN-484192 | + | no | 4.9E-07 |
| EN-484501 | + | no | 1.1E-07 |
| EN-484571 | + | no | 3.1E-08 |
| EN-485249 | + | yes | 1.6E-08 |
| EN-486443 | + | no | 0.0E+00 |
| EN-487498 | + | no | 1.1E-07 |
| EN-487827 | + | no | 3.1E-08 |
| EN-488128 | + | no | 1.1E-07 |
| EN-488253 | + | no | 1.7E-07 |
| EN-488566 | + | no | 9.4E-08 |
| LE-488742 | + | only | n/a |
| EN-489143-46 | + | no | 1.2E-06 |
| EN-490993 | + | no | 7.9E-08 |
| EN-491040-42 | - | no | 3.1E-08 |
| EN-491091 | - | yes | 4.6E-08 |
| EN-491822 | - | no | 0.0E+00 |
| EN-492652-54 | + | yes | 1.4E-06 |
| EN-492826 | + | no | 5.7E-07 |
| EN-493108 | + | no | 3.1E-08 |
| EN-493332 | + | no | 1.6E-08 |
| EN-493648 | - | no | 1.5E-07 |
| EN-494083 | + | no | 2.4E-07 |
| EN-494477 | - | no | 1.5E-07 |
| EN-494493 | + | no | 1.6E-05 |
| EN-494640 | + | yes | 6.0E-07 |
| EN-495209 | + | no | 4.6E-07 |
| EN-495299 | + | no | 6.9E-07 |
| EN-495517 | + | no | 4.9E-07 |
| EN-496254 | - | no | 1.1E-06 |
| EN-496346 | - | no | 2.0E-06 |
| EN-497820 | - | no | 1.4E-06 |
| LE-497957 | + | only | n/a |
| EN-499019 | + | no | 0.0E+00 |
| EN-499598 | - | no | 6.2E-08 |
| EN-500499-02 | + | no | 0.0E+00 |
| EN-500592 | - | no | 0.0E+00 |
| EN-501976 | + | no | 0.0E+00 |
| EN-502027 | + | yes | 5.7E-07 |
| EN-502589 | + | no | 1.9E-06 |
| EN-504061 | + | no | 4.7E-08 |
| EN-505043 | + | no | 3.3E-07 |
| EN-505259 | + | no | 3.1E-08 |
| EN-506156 | + | no | 2.4E-07 |
| EN-506530 | - | no | 4.6E-08 |
| EN-507191 | - | yes | 1.2E-07 |
| EN-507805 | - | no | 0.0E+00 |
| EN-508453 | + | no | 0.0E+00 |
| EN-509178 | + | yes | 0.0E+00 |
| EN-509636 | - | no | 6.2E-08 |
| EN-509648 | - | no | 1.8E-07 |
| EN-509778 | + | no | 1.1E-07 |
| EN-512052 | + | no | 4.7E-08 |
| EN-512692 | + | no | 0.0E+00 |
| EN-512993 | + | no | 0.0E+00 |
| EN-513430 | + | no | 0.0E+00 |
| EN-513502 | + | no | 0.0E+00 |
| EN-515007 | + | no | 0.0E+00 |
| EN-515180 | - | yes | 0.0E+00 |
| EN-515877 | - | no | 1.8E-07 |
| EN-516236 | - | no | 0.0E+00 |
| EN-516434 | + | no | 0.0E+00 |
| EN-517178 | + | no | 1.1E-07 |
| EN-518818 | - | yes | 1.5E-08 |
| EN-518907 | + | no | 0.0E+00 |
| EN-519206 | + | no | 6.5E-07 |
| EN-519370 | - | no | 2.5E-07 |
| EN-520004-07 | + | no | 1.6E-08 |
| EN-520185 | + | no | 4.7E-08 |
| EN-521395 | + | no | 1.6E-08 |
| EN-521923 | - | no | 2.0E-07 |
| EN-521967 | - | no | 1.2E-06 |
| EN-522033 | - | yes | 0.0E+00 |
| EN-522621 | + | no | 0.0E+00 |
| EN-523593 | - | no | 2.9E-07 |
| EN-523981 | + | yes | 1.1E-07 |
| EN-525729 | + | yes | 0.0E+00 |
| EN-526608 | + | no | 1.1E-07 |
| EN-527105 | - | yes | 1.5E-08 |
| LE-527221-23 | + | only | n/a |
| EN-527440 | - | no | 0.0E+00 |
| EN-527495 | + | no | 0.0E+00 |
| EN-527845 | + | no | 0.0E+00 |
| EN-528088 | + | no | 4.7E-08 |
| EN-528169 | + | no | 0.0E+00 |
| EN-528492 | + | no | 8.4E-06 |
| EN-529662 | - | no | 2.5E-07 |
| EN-529719 | + | no | 1.1E-07 |
| EN-531196 | + | no | 1.6E-08 |
| EN-531916-22 | - | yes | 4.6E-08 |
| EN-531940 | + | yes | 3.1E-08 |
| EN-531960 | + | no | 0.0E+00 |
| EN-532076-84 | + | yes | 1.0E-06 |
| EN-534002 | + | yes | 0.0E+00 |
| EN-534178 | - | no | 1.5E-08 |
| EN-534248-52 | + | no | 5.3E-07 |
| EN-535438 | + | yes | 1.2E-06 |
| EN-536160 | - | no | 1.5E-08 |
| EN-537166-69 | + | no | 1.6E-08 |
| LE-537408 | + | only | n/a |
| EN-537907 | + | no | 4.7E-08 |
| EN-538112 | + | no | 0.0E+00 |
| EN-538424 | - | yes | 1.1E-07 |
| LE-539795 | - | only | n/a |
| EN-541852 | - | no | 9.2E-08 |
| EN-541866 | - | no | 1.2E-07 |
| EN-542221 | - | no | 0.0E+00 |
| EN-542988 | + | no | 1.6E-08 |
| EN-544123-25 | - | yes | 3.8E-06 |
| EN-544181 | - | no | 6.2E-08 |
| EN-544192 | + | yes | 4.4E-07 |
| EN-544749 | + | no | 0.0E+00 |
| EN-545415 | - | no | 4.6E-08 |
| EN-545792-94 | + | no | 9.4E-08 |
| EN-546293 | + | yes | 0.0E+00 |
| EN-546346 | + | no | 3.1E-08 |
| EN-546359 | + | no | 6.4E-06 |
| EN-547297 | + | yes | 4.3E-06 |
| EN-547437-39 | + | no | 2.7E-06 |
| EN-547842 | - | yes | 1.1E-07 |
| EN-548418 | + | no | 0.0E+00 |
| EN-549078 | + | no | 0.0E+00 |
| EN-550559 | + | yes | 2.4E-07 |
| EN-551362 | - | no | 6.2E-08 |
| EN-551700 | - | no | 4.6E-08 |
| EN-551864-67 | + | yes | 0.0E+00 |
| EN-552041 | - | yes | 3.6E-06 |
| EN-552054 | - | no | 9.2E-08 |
| EN-552141 | + | no | 1.6E-06 |
| EN-552464 | - | yes | 1.8E-07 |
| EN-552473 | + | yes | 0.0E+00 |
| EN-553060 | - | no | 0.0E+00 |
| EN-553077 | + | no | 1.6E-08 |
| EN-553432 | + | no | 0.0E+00 |
| EN-553608-10 | + | no | 2.8E-07 |
| EN-553911 | + | no | 1.6E-08 |
| EN-554797 | - | no | 0.0E+00 |
| EN-555887-90 | + | no | 3.1E-08 |
| EN-556186 | + | no | 0.0E+00 |
| EN-556200 | + | yes | 1.6E-08 |
| EN-556242 | - | no | 0.0E+00 |
| EN-556256 | - | no | 3.1E-08 |
| EN-556261 | + | no | 2.4E-07 |
| EN-556855 | + | no | 0.0E+00 |
| EN-557312 | + | yes | 4.7E-08 |
| EN-560752 | + | no | 0.0E+00 |
| EN-561218 | + | no | 9.4E-08 |
| EN-561546 | + | yes | 5.0E-05 |
| EN-561968 | - | no | 9.2E-08 |
| EN-562036 | + | no | 4.7E-08 |
| EN-563416 | - | no | 1.2E-07 |
| EN-563433-36 | + | no | 0.0E+00 |
| EN-564058 | - | yes | 1.4E-06 |
| EN-564174 | + | no | 0.0E+00 |
| EN-564200 | + | no | 2.5E-07 |
| EN-564271 | + | no | 1.1E-06 |
| EN-564488-90 | + | no | 3.1E-08 |
| EN-564738 | + | no | 2.5E-07 |
| EN-564893 | - | no | 2.0E-07 |
| EN-564970 | + | no | 4.4E-07 |
| EN-564981 | + | no | 4.7E-08 |
| EN-565039 | + | no | 3.6E-07 |
| EN-565188 | + | yes | 3.1E-07 |
| EN-565787 | + | yes | 2.8E-07 |
| EN-565816 | - | no | 0.0E+00 |
| EN-565896 | + | no | 1.4E-06 |
| EN-566387 | + | no | 3.3E-07 |
| EN-566401 | + | no | 4.4E-07 |
| EN-567634 | + | no | 1.6E-08 |
| EN-567938 | + | no | 3.3E-07 |
| EN-568231 | + | no | 0.0E+00 |
| EN-569551 | + | yes | 9.4E-08 |
| EN-569577 | + | no | 4.4E-06 |
| EN-569734 | - | no | 2.3E-07 |
| EN-570330-33 | + | no | 0.0E+00 |
| EN-570565 | + | no | 7.9E-08 |
| EN-570605 | - | no | 7.2E-06 |
| EN-572184 | + | no | 1.1E-07 |
| LE-574372 | - | only | n/a |
| EN-574701 | + | no | 1.1E-07 |
| EN-574985 | + | yes | 4.7E-08 |
| EN-576235 | - | no | 3.1E-08 |
| EN-576629 | - | no | 1.5E-08 |
| EN-577300 | + | no | 2.7E-07 |
| EN-577576 | - | no | 1.5E-07 |
| EN-578338 | - | yes | 1.5E-07 |
| EN-578437 | + | no | 0.0E+00 |
| EN-578506 | - | yes | 4.2E-07 |
| EN-579856 | + | no | 1.6E-08 |
| LE-580556 | + | only | n/a |
| EN-580673 | + | no | 0.0E+00 |
| EN-581080 | + | yes | 5.7E-07 |
| EN-582851 | + | no | 2.8E-07 |
| EN-583253 | - | yes | 2.0E-07 |
| EN-583316-18 | + | yes | 1.2E-06 |
| EN-583471 | + | no | 0.0E+00 |
| EN-584375 | - | no | 2.2E-07 |
| EN-585839 | + | yes | 4.4E-07 |
| EN-586175 | - | yes | 0.0E+00 |
| EN-586767 | + | no | 9.4E-08 |
| EN-588312 | + | no | 0.0E+00 |
| EN-588494-96 | + | yes | 0.0E+00 |
| EN-588593 | - | no | 0.0E+00 |
| EN-589092 | + | no | 1.6E-08 |
| EN-589140 | + | no | 5.4E-06 |
| EN-592680 | - | no | 7.8E-07 |
| EN-593513-16 | + | yes | 0.0E+00 |
| EN-593679 | - | no | 0.0E+00 |
| EN-593698 | + | no | 0.0E+00 |
| EN-594570 | + | no | 0.0E+00 |
| EN-595353 | + | no | 1.6E-08 |
| EN-597113 | + | yes | 1.2E-05 |
| EN-597150 | + | no | 3.1E-08 |
| EN-598138 | + | no | 0.0E+00 |
| EN-598423 | + | no | 9.4E-08 |
| LE-599029 | + | only | n/a |
| EN-600895 | + | no | 0.0E+00 |
| EN-601083 | - | yes | 9.2E-08 |
| EN-601107 | + | no | 0.0E+00 |
| EN-601203 | + | no | 1.1E-07 |
| LE-601249-51 | - | only | n/a |
| EN-602468 | + | yes | 4.7E-08 |
| EN-602502-04 | + | yes | 3.3E-07 |
| EN-602708 | + | no | 3.3E-07 |
| EN-602759-67 | + | yes | 0.0E+00 |
| EN-603401 | + | no | 1.6E-08 |
| EN-603428 | + | no | 1.1E-07 |
| EN-603495-98 | + | no | 0.0E+00 |
| EN-604412 | - | no | 6.2E-08 |
| EN-604969 | + | yes | 0.0E+00 |
| EN-605435 | - | no | 0.0E+00 |
| EN-605570-72 | + | yes | 1.1E-07 |
| EN-605678-81 | + | yes | 0.0E+00 |
| EN-605784-87 | + | yes | 0.0E+00 |
| EN-605881-83 | + | no | 4.7E-06 |
| EN-606031 | + | no | 4.7E-08 |
| EN-607385 | + | no | 1.9E-06 |
| EN-607854 | + | no | 9.4E-08 |
| EN-607870-77 | + | no | 5.0E-07 |
| EN-608951 | + | no | 2.1E-06 |
| EN-610171 | + | no | 2.4E-05 |
| EN-611466 | - | no | 0.0E+00 |
| EN-611596-04 | - | yes | 0.0E+00 |
| EN-611606 | - | yes | 4.6E-08 |
| EN-611936 | + | no | 1.6E-08 |
| EN-612055 | - | no | 1.1E-07 |
| EN-612112 | + | yes | 0.0E+00 |
| EN-612329 | + | no | 1.6E-08 |
| EN-612427 | + | no | 9.1E-07 |
| EN-612672 | + | yes | 2.0E-06 |
| EN-613949-52 | + | no | 1.6E-08 |
| EN-614292 | + | no | 0.0E+00 |
| EN-614514 | - | no | 3.1E-08 |
| EN-614532 | + | no | 5.7E-07 |
| EN-615197 | - | no | 0.0E+00 |
| EN-615214 | + | no | 1.1E-07 |
| EN-615612 | - | yes | 1.5E-08 |
| EN-615874-77 | + | no | 0.0E+00 |
| EN-616371 | - | yes | 2.5E-07 |
| EN-616864 | + | no | 1.6E-08 |
| EN-617052 | + | no | 2.8E-07 |
| EN-617131 | + | no | 0.0E+00 |
| EN-618789-93 | + | yes | 0.0E+00 |
| EN-619022-25 | + | yes | 2.7E-06 |
| EN-619864 | + | no | 0.0E+00 |
| EN-620292 | + | yes | 4.4E-06 |
| EN-620325 | + | no | 2.0E-07 |
| EN-621885-87 | - | yes | 2.5E-07 |
| EN-622325 | - | no | 6.2E-08 |
| EN-622360 | + | yes | 0.0E+00 |
| EN-623505 | - | no | 1.5E-08 |
| EN-623718 | - | no | 1.7E-07 |
| EN-624369 | + | no | 1.6E-08 |
| EN-625198 | + | no | 0.0E+00 |
| EN-625488 | - | yes | 1.9E-06 |
| EN-626366 | - | no | 9.2E-08 |
| EN-626378 | + | no | 0.0E+00 |
| EN-627215 | + | no | 3.3E-07 |
| EN-627846 | + | no | 9.4E-08 |
| EN-628657 | + | yes | 0.0E+00 |
| EN-628737 | + | no | 0.0E+00 |
| EN-629901 | - | yes | 6.3E-07 |
| EN-631418 | + | no | 1.6E-08 |
| EN-631754 | + | no | 0.0E+00 |
| EN-632302 | - | no | 6.2E-08 |
| EN-632907 | + | yes | 5.5E-07 |
| EN-634069 | + | no | 3.1E-08 |
| EN-634222 | + | no | 1.6E-08 |
| EN-634271 | + | no | 1.6E-08 |
| LE-635103 | - | only | n/a |
| EN-635190 | + | yes | 8.3E-07 |
| EN-635461 | - | no | 1.5E-08 |
| EN-636184 | - | no | 4.6E-08 |
| EN-636614-17 | + | yes | 0.0E+00 |
| EN-637760 | + | no | 0.0E+00 |
| EN-638402 | + | no | 1.1E-07 |
| EN-639156 | - | no | 0.0E+00 |
| EN-639204-06 | - | yes | 1.1E-06 |
| EN-639212-15 | + | yes | 0.0E+00 |
| EN-639334 | + | no | 1.7E-07 |
| EN-639665 | + | yes | 1.4E-07 |
| EN-640548 | + | no | 4.7E-08 |
| EN-640955 | + | yes | 1.9E-06 |
| EN-641678 | - | no | 1.1E-07 |
| EN-642234 | - | yes | 2.8E-06 |
| EN-642240 | + | no | 3.1E-08 |
| EN-642334 | + | no | 3.1E-08 |
| EN-642345-47 | + | yes | 6.0E-07 |
| EN-642501 | + | no | 0.0E+00 |
| EN-642622 | + | no | 0.0E+00 |
| EN-642633 | + | no | 1.6E-08 |
| EN-642687 | + | no | 2.8E-07 |
| EN-642974 | + | yes | 0.0E+00 |
| EN-643908 | + | no | 3.3E-07 |
| EN-645928 | + | yes | 1.6E-08 |
| EN-646981 | - | no | 0.0E+00 |
| EN-647128 | - | no | 6.2E-08 |
| EN-647171 | - | no | 2.0E-07 |
| EN-647919 | - | no | 1.2E-07 |
| EN-647942 | + | no | 0.0E+00 |
| LE-647993 | - | only | n/a |
| EN-648162 | + | no | 0.0E+00 |
| EN-648724 | + | no | 3.3E-07 |
| EN-649120 | - | no | 0.0E+00 |
| EN-649169 | + | no | 0.0E+00 |
| EN-649446 | + | yes | 4.7E-08 |
| EN-649779 | + | no | 1.1E-07 |
| EN-649910 | + | no | 3.3E-07 |
| EN-651011 | + | yes | 2.0E-07 |
| EN-652906 | + | no | 0.0E+00 |
| EN-653807 | + | no | 8.3E-07 |
| EN-654724 | + | no | 0.0E+00 |
| EN-655516-19 | + | no | 0.0E+00 |
| EN-656662 | + | no | 2.2E-07 |
| EN-656686 | - | no | 1.0E-06 |
| EN-656880 | + | yes | 0.0E+00 |
| EN-657179 | - | no | 3.1E-08 |
| EN-659320 | - | yes | 9.2E-08 |
| EN-659339-45 | + | no | 1.7E-07 |
| EN-659887 | + | no | 0.0E+00 |
| EN-660790 | + | no | 0.0E+00 |
| EN-660912-15 | + | no | 1.6E-08 |
| EN-660977 | + | no | 9.4E-08 |
| EN-661312 | + | no | 3.3E-07 |
| EN-661438 | + | no | 0.0E+00 |
| EN-662257-59 | + | yes | 0.0E+00 |
| EN-662382 | - | no | 0.0E+00 |
| EN-662829 | - | yes | 0.0E+00 |
| EN-663501 | + | no | 1.1E-07 |
| EN-663678 | + | no | 2.9E-06 |
| EN-663930 | - | no | 0.0E+00 |
| EN-664204-06 | + | yes | 7.7E-07 |
| EN-664498 | - | no | 6.9E-07 |
| LE-664516 | - | only | n/a |
| EN-664773 | + | no | 4.7E-08 |
| EN-665658 | - | yes | 0.0E+00 |
| EN-666181 | + | yes | 8.3E-07 |
| EN-666265 | - | no | 6.9E-07 |
| EN-666269-73 | + | no | 0.0E+00 |
| EN-667037-40 | + | no | 2.2E-07 |
| EN-667454 | + | yes | 0.0E+00 |
| EN-667589 | + | no | 1.6E-08 |
| EN-668276 | + | yes | 0.0E+00 |
| EN-669584 | + | no | 1.6E-08 |
| EN-671543 | + | no | 2.8E-07 |
| EN-672361-63 | + | no | 1.6E-08 |
| EN-675627 | - | yes | 1.5E-08 |
| EN-675801 | + | no | 0.0E+00 |
| EN-676052 | + | no | 5.6E-06 |
| EN-676374 | - | no | 1.5E-08 |
| EN-676533 | - | no | 0.0E+00 |
| EN-676656 | + | no | 1.3E-07 |
| EN-679486 | + | no | 1.6E-08 |
| EN-680445 | - | no | 6.2E-08 |
| EN-680988 | - | no | 1.2E-05 |
| EN-681127 | - | no | 0.0E+00 |
| EN-681481-83 | + | no | 1.1E-07 |
| EN-682570 | - | no | 6.2E-08 |
| EN-682587 | + | no | 4.7E-08 |
| EN-683369 | - | no | 1.6E-06 |
| EN-684810 | - | no | 2.3E-06 |
| EN-688106 | + | no | 1.1E-07 |
| EN-688390-92 | + | no | 5.3E-07 |
| EN-689518 | - | yes | 1.2E-06 |
| EN-690030-32 | + | yes | 1.4E-06 |
| EN-690108 | - | no | 0.0E+00 |
| EN-690471 | + | yes | 3.0E-06 |
| EN-690675 | + | no | 1.6E-08 |
| EN-691492 | + | no | 4.7E-07 |
| EN-691848 | - | no | 1.5E-08 |
| EN-693527 | - | no | 1.1E-07 |
| EN-693542-45 | + | yes | 0.0E+00 |
| EN-693693-01 | + | no | 1.8E-05 |
| EN-694880 | + | no | 1.1E-07 |
| EN-696798 | - | no | 0.0E+00 |
| EN-696911-13 | - | yes | 2.5E-07 |
| EN-698253 | + | no | 1.6E-08 |
| EN-699470 | + | yes | 0.0E+00 |
| EN-701582 | - | no | 1.1E-07 |
| EN-701664 | + | yes | 1.1E-07 |
| EN-702404-09 | + | no | 4.7E-07 |
| EN-702803-07 | + | no | 3.1E-08 |
| EN-703102 | + | yes | 9.8E-07 |
| EN-703209 | + | no | 2.8E-07 |
| EN-704600 | + | no | 3.1E-08 |
| EN-704662 | + | no | 9.4E-08 |
| EN-704693 | + | no | 6.3E-07 |
| EN-706648 | - | yes | 0.0E+00 |
| EN-706656 | - | yes | 2.3E-07 |
| EN-706704 | + | yes | 0.0E+00 |
| EN-707657 | + | yes | 0.0E+00 |
| EN-708078 | - | no | 0.0E+00 |
| EN-709082 | + | no | 0.0E+00 |
| EN-709796-98 | - | no | 2.8E-06 |
| EN-710916-20 | + | no | 1.1E-07 |
| EN-711014 | - | no | 6.3E-07 |
| EN-712847-49 | - | yes | 1.8E-06 |
| EN-712867 | - | yes | 0.0E+00 |
| EN-712934 | + | yes | 0.0E+00 |
| EN-713876 | - | yes | 0.0E+00 |
| EN-713937 | + | yes | 0.0E+00 |
| EN-715551 | - | no | 0.0E+00 |
| EN-716094 | - | yes | 2.3E-07 |
| EN-716303 | + | yes | 5.5E-07 |
| EN-716782-84 | - | no | 2.3E-07 |
| EN-717439 | + | no | 8.2E-07 |
| EN-717464-67 | + | yes | 7.9E-08 |
| LE-718285 | + | only | n/a |
| EN-718577 | + | no | 2.5E-07 |
| EN-718682 | - | no | 4.6E-08 |
| EN-718684 | + | no | 9.4E-08 |
| EN-719343 | + | yes | 4.7E-08 |
| EN-720186 | + | yes | 0.0E+00 |
| EN-720558 | - | no | 0.0E+00 |
| EN-720928 | + | no | 0.0E+00 |
| EN-721330 | - | yes | 0.0E+00 |
| EN-721834 | + | no | 2.8E-07 |
| EN-725033 | + | yes | 5.0E-07 |
| EN-725963 | - | no | 2.2E-06 |
| EN-726057 | - | no | 2.2E-07 |
| EN-726469 | - | no | 1.5E-08 |
| EN-726484 | + | no | 4.7E-08 |
| EN-727512 | - | no | 4.6E-08 |
| EN-727624-26 | - | yes | 7.7E-08 |
| EN-728897 | - | yes | 9.2E-08 |
| EN-728919 | - | no | 2.0E-07 |
| EN-728967 | + | yes | 6.9E-07 |
| EN-730218-21 | + | no | 6.5E-06 |
| EN-731133 | - | no | 6.2E-08 |
| EN-731267 | - | no | 2.0E-07 |
| EN-731285 | + | no | 0.0E+00 |
| EN-734483 | - | no | 2.0E-07 |
| EN-734720-23 | - | no | 1.2E-07 |
| EN-735521-23 | - | yes | 0.0E+00 |
| EN-736038 | + | no | 7.9E-08 |
| EN-736470 | - | no | 6.2E-08 |
| EN-736709 | - | no | 7.5E-07 |
| EN-736731 | + | yes | 7.9E-08 |
| EN-737101 | + | no | 0.0E+00 |
| EN-737853 | - | yes | 0.0E+00 |
| EN-737889 | + | no | 7.9E-08 |
| EN-739617 | - | yes | 0.0E+00 |
| EN-740139 | - | yes | 2.3E-07 |
| EN-740158 | + | yes | 0.0E+00 |
| EN-740225 | + | no | 2.5E-07 |
| EN-740336 | - | no | 1.5E-08 |
| EN-740532 | + | no | 0.0E+00 |
| EN-740636 | + | no | 4.7E-08 |
| EN-741297-03 | + | no | 0.0E+00 |
| EN-742246 | + | no | 1.1E-07 |
| EN-742347 | + | no | 4.7E-08 |
| EN-742426 | + | no | 0.0E+00 |
| LE-743910-13 | - | only | n/a |
| LE-744036 | + | only | n/a |
| EN-744509-11 | + | no | 2.4E-07 |
| EN-744640 | - | no | 9.2E-08 |
| EN-745051 | + | yes | 0.0E+00 |
| EN-747088-90 | + | no | 1.6E-08 |
| EN-747508 | + | no | 1.1E-07 |
| LE-750162 | - | only | n/a |
| EN-750299 | + | no | 0.0E+00 |
| EN-756064 | + | no | 0.0E+00 |
| EN-756200 | + | yes | 0.0E+00 |
| EN-756254 | + | no | 1.6E-08 |
| EN-756441 | + | yes | 2.8E-07 |
| EN-756551 | + | no | 7.9E-08 |
| EN-756576-78 | + | no | 1.6E-08 |
| EN-757487 | + | no | 2.7E-07 |
| EN-757535 | - | yes | 0.0E+00 |
| EN-757693 | + | no | 4.7E-08 |
| EN-758449 | + | no | 1.6E-08 |
| EN-759849 | - | yes | 1.1E-07 |
| EN-760202 | + | no | 0.0E+00 |
| EN-760777 | - | no | 2.6E-05 |
| EN-761600 | - | yes | 1.2E-07 |
| EN-762557 | - | no | 0.0E+00 |
| EN-762867 | - | no | 0.0E+00 |
| EN-763005-09 | + | yes | 0.0E+00 |
| EN-763546 | + | no | 0.0E+00 |
| EN-764060 | - | no | 3.1E-07 |
| EN-764806-08 | - | no | 0.0E+00 |
| EN-764909 | + | yes | 1.6E-08 |
| EN-764914-16 | - | no | 1.5E-08 |
| EN-764939 | + | no | 4.7E-08 |
| EN-765190 | - | no | 1.5E-08 |
| EN-765230 | + | no | 0.0E+00 |
| EN-766492 | - | yes | 9.2E-08 |
| EN-767266 | - | no | 4.0E-06 |
| EN-767490 | - | no | 1.1E-07 |
| EN-768211 | - | yes | 0.0E+00 |
| EN-768284 | + | no | 0.0E+00 |
| EN-768847-55 | - | no | 2.3E-07 |
| EN-768894 | - | no | 1.5E-07 |
| EN-769717 | - | yes | 0.0E+00 |
| EN-769732 | + | yes | 4.2E-07 |
| EN-769776 | + | no | 1.6E-08 |
| EN-769973 | + | no | 1.6E-08 |
| EN-770258 | - | no | 0.0E+00 |
| EN-771016 | + | no | 0.0E+00 |
| EN-771405 | - | no | 0.0E+00 |
| EN-771571 | + | yes | 8.7E-06 |
| EN-772105 | + | no | 2.8E-07 |
| EN-773802 | + | no | 4.7E-08 |
| EN-775687 | + | no | 1.2E-06 |
| EN-775876 | + | no | 0.0E+00 |
| EN-776372 | - | no | 2.3E-07 |
| EN-776546 | + | yes | 1.3E-06 |
| EN-777146 | + | no | 0.0E+00 |
| EN-778327 | - | yes | 0.0E+00 |
| EN-778680 | + | no | 3.1E-08 |
| EN-778944 | - | no | 0.0E+00 |
| EN-778972-75 | + | no | 3.1E-08 |
| EN-779290 | - | no | 0.0E+00 |
| EN-779830 | - | no | 8.8E-06 |
| EN-781140-42 | + | no | 5.7E-07 |
| EN-781355 | - | yes | 3.1E-06 |
| EN-781388 | + | yes | 0.0E+00 |
| EN-781414 | + | no | 7.9E-08 |
| EN-781479-82 | + | no | 4.4E-07 |
| EN-781876 | + | no | 1.6E-08 |
| EN-784858 | + | no | 4.7E-06 |
| EN-785643-45 | + | no | 7.9E-08 |
| EN-786039 | - | yes | 0.0E+00 |
| EN-786138 | + | yes | 0.0E+00 |
| EN-786344 | + | no | 2.8E-07 |
| EN-787428 | + | no | 3.1E-08 |
| EN-787439 | - | no | 0.0E+00 |
| EN-787442 | + | no | 0.0E+00 |
| EN-787980-88 | + | no | 0.0E+00 |
| EN-788348 | + | yes | 2.4E-07 |
| EN-788785 | + | no | 1.6E-08 |
| EN-788985 | - | no | 7.7E-08 |
| LE-789397 | + | only | n/a |
| EN-789647 | - | yes | 0.0E+00 |
| EN-789739 | - | no | 1.2E-06 |
| EN-789757-59 | + | yes | 0.0E+00 |
| EN-789954 | + | no | 1.6E-08 |
| EN-790057 | - | no | 2.2E-07 |
| EN-790188 | + | no | 7.1E-07 |
| EN-790876-79 | + | no | 9.4E-08 |
| EN-791700 | + | no | 0.0E+00 |
| EN-792369 | - | no | 0.0E+00 |
| EN-792471 | + | no | 1.1E-07 |
| EN-792747 | - | yes | 1.5E-06 |
| EN-792921-23 | + | yes | 0.0E+00 |
| EN-793221 | - | no | 1.1E-07 |
| EN-794094 | - | no | 0.0E+00 |
| EN-795729-31 | + | yes | 3.1E-08 |
| EN-795754 | - | yes | 0.0E+00 |
| EN-796044 | + | no | 1.3E-07 |
| EN-796421 | + | no | 6.7E-06 |
| EN-796868 | + | no | 1.1E-07 |
| EN-797318 | + | no | 0.0E+00 |
| EN-797593 | + | yes | 2.0E-07 |
| EN-797999 | - | no | 0.0E+00 |
| EN-798093 | + | no | 0.0E+00 |
| EN-798816 | + | no | 3.9E-06 |
| EN-799620 | + | no | 0.0E+00 |
| EN-799620 | - | no | 3.1E-08 |
| EN-800436 | - | no | 0.0E+00 |
| EN-800784 | - | yes | 0.0E+00 |
| LE-802023-25 | + | only | n/a |
| EN-802402 | - | yes | 0.0E+00 |
| EN-803032 | - | no | 0.0E+00 |
| EN-803985 | - | no | 9.2E-08 |
| EN-804077 | + | no | 1.9E-07 |
| EN-804313 | + | no | 1.7E-07 |
| EN-804800 | - | no | 9.2E-08 |
| EN-806108 | - | no | 4.6E-08 |
| EN-806619 | + | no | 9.4E-08 |
| LE-806894-97 | - | only | n/a |
| EN-807476 | + | yes | 0.0E+00 |
| EN-807683 | - | no | 0.0E+00 |
| EN-808051 | - | no | 1.1E-07 |
| EN-808212 | + | no | 9.4E-08 |
| LE-808900 | + | only | n/a |
| EN-809848 | + | no | 1.1E-07 |
| EN-809911 | + | no | 4.7E-08 |
| EN-810002 | - | no | 6.2E-08 |
| EN-810034 | - | no | 6.2E-08 |
| EN-811478 | + | no | 0.0E+00 |
| EN-811589 | + | no | 1.1E-07 |
| EN-811600 | + | no | 3.9E-07 |
| EN-812460 | + | no | 1.1E-07 |
| EN-812780 | + | no | 0.0E+00 |
| EN-813000 | - | no | 3.1E-08 |
| EN-813051 | - | no | 0.0E+00 |
| EN-814283 | - | no | 3.5E-07 |
| EN-814409 | - | no | 0.0E+00 |
| EN-814751 | + | no | 6.0E-07 |
| EN-815686 | - | no | 2.6E-07 |
| LE-815801 | + | only | n/a |
| EN-816829 | - | no | 1.7E-07 |
| EN-817744 | - | no | 9.2E-08 |
| EN-817762 | - | yes | 2.3E-07 |
| EN-817834 | + | yes | 3.2E-06 |
| EN-818155 | - | no | 1.1E-07 |
| EN-820062 | - | no | 7.8E-07 |
| EN-822210 | + | no | 0.0E+00 |
| LE-822851 | - | only | n/a |
| LE-823052 | + | only | n/a |
| EN-824741 | - | no | 1.1E-07 |
| EN-824817 | - | yes | 1.7E-07 |
| EN-826913 | + | no | 1.6E-06 |
| EN-827427-30 | + | no | 0.0E+00 |
| EN-827470 | + | no | 1.6E-08 |
| EN-828355 | + | no | 9.4E-08 |
| EN-829066 | + | no | 0.0E+00 |
| EN-830012 | + | no | 5.0E-07 |
| EN-831040 | + | no | 5.0E-07 |
| EN-832810 | + | no | 2.4E-07 |
| EN-833200-03 | + | no | 2.8E-07 |
| EN-833594 | + | no | 0.0E+00 |
| EN-835090 | + | no | 4.7E-08 |
| EN-835978-80 | + | no | 1.4E-07 |
| EN-837030-32 | - | no | 2.3E-07 |
| EN-837316 | + | yes | 4.6E-07 |
| EN-838891 | + | no | 0.0E+00 |
| EN-838968 | - | no | 5.4E-07 |
| EN-839001 | + | no | 4.7E-07 |
| EN-839151 | + | no | 2.5E-07 |
| EN-839464-66 | + | yes | 1.6E-08 |
| EN-839476-79 | + | yes | 2.5E-06 |
| EN-840090 | + | no | 1.1E-07 |
| EN-840189 | + | no | 0.0E+00 |
| EN-840333-35 | + | no | 2.0E-07 |
| EN-840418 | + | yes | 2.8E-07 |
| EN-841188 | - | no | 3.1E-08 |
| EN-841657 | + | no | 2.4E-07 |
| EN-841711 | + | yes | 0.0E+00 |
| EN-842478 | + | no | 1.3E-06 |
| EN-843738 | - | no | 6.8E-07 |
| EN-843812 | + | yes | 2.0E-07 |
| EN-843836 | + | no | 0.0E+00 |
| EN-844811 | - | no | 9.2E-08 |
| EN-844898 | + | no | 0.0E+00 |
| EN-846422 | - | yes | 0.0E+00 |
| EN-846563-65 | + | yes | 3.1E-08 |
| EN-847721 | - | no | 1.5E-07 |
| EN-847733 | + | yes | 4.4E-07 |
| EN-851078 | + | no | 1.1E-06 |
| EN-851922 | + | no | 1.3E-07 |
| EN-852759 | + | no | 6.1E-06 |
| LE-854372 | - | only | n/a |
| EN-854380 | + | no | 1.0E-06 |
| EN-854389-94 | + | yes | 1.3E-05 |
| EN-854492 | + | yes | 1.6E-08 |
| EN-856206 | - | no | 0.0E+00 |
| EN-856770 | - | no | 1.5E-06 |
| EN-857711 | + | no | 3.1E-06 |
| EN-857806 | + | no | 0.0E+00 |
| EN-859207-09 | + | yes | 1.1E-07 |
| EN-859313 | + | no | 7.9E-08 |
| EN-861126 | - | no | 9.2E-08 |
| EN-861867 | - | no | 6.2E-08 |
| EN-863403 | + | no | 2.4E-07 |
| EN-863415-17 | + | no | 3.9E-06 |
| EN-863550 | + | no | 0.0E+00 |
| EN-863795-99 | + | no | 2.4E-07 |
| EN-864160-63 | + | no | 4.7E-08 |
| EN-865227 | + | no | 3.1E-08 |
| EN-866224 | - | no | 0.0E+00 |
| EN-866461 | + | no | 1.6E-08 |
| EN-867089-93 | + | no | 3.1E-08 |
| EN-868543 | + | no | 0.0E+00 |
| EN-868685-88 | + | no | 1.6E-08 |
| EN-869077 | - | no | 6.2E-08 |
| EN-869095 | + | no | 0.0E+00 |
| EN-869218 | + | yes | 1.0E-06 |
| EN-869856 | - | no | 0.0E+00 |
| EN-869880 | + | no | 0.0E+00 |
| EN-871263 | + | yes | 5.0E-07 |
| EN-874475-79 | + | yes | 2.3E-06 |
| EN-875095 | + | no | 0.0E+00 |
| EN-876261 | - | no | 4.6E-08 |
| EN-878276 | + | no | 0.0E+00 |
| EN-878993 | - | no | 9.2E-08 |
| EN-879027 | - | no | 4.6E-07 |
| EN-879204 | + | yes | 3.1E-08 |
| EN-879577 | + | yes | 0.0E+00 |
| EN-879619 | + | no | 3.1E-08 |
| EN-879790 | - | no | 4.6E-08 |
| EN-879932-34 | - | yes | 9.2E-08 |
| EN-880097 | - | no | 1.8E-07 |
| EN-880117 | + | no | 3.1E-08 |
| EN-880277 | - | no | 0.0E+00 |
| EN-880560 | + | no | 9.4E-08 |
| EN-880971 | + | yes | 0.0E+00 |
| EN-881382-84 | - | no | 2.3E-07 |
| EN-881422 | + | no | 6.9E-07 |
| EN-881738 | + | no | 0.0E+00 |
| EN-882313 | - | no | 0.0E+00 |
| EN-882712 | - | no | 4.2E-07 |
| EN-883321 | + | no | 0.0E+00 |
| EN-883575-78 | + | no | 1.1E-07 |
| EN-883881-83 | - | yes | 2.3E-07 |
| EN-884878 | - | no | 0.0E+00 |
| EN-885153 | + | no | 6.5E-07 |
| EN-885271-75 | + | yes | 0.0E+00 |
| EN-886162 | - | no | 4.6E-08 |
| EN-886213 | - | yes | 0.0E+00 |
| EN-886233 | - | no | 2.2E-07 |
| EN-886312 | + | yes | 2.0E-06 |
| EN-886318 | - | no | 0.0E+00 |
| EN-886435 | - | no | 0.0E+00 |
| EN-888889 | + | no | 3.6E-07 |
| EN-889785 | + | no | 0.0E+00 |
| EN-890457 | - | no | 0.0E+00 |
| EN-890564 | + | yes | 3.3E-06 |
| EN-891641 | + | no | 2.4E-07 |
| EN-892305-07 | + | yes | 5.0E-07 |
| EN-892475 | + | no | 3.6E-07 |
| EN-894159 | - | no | 1.1E-07 |
| EN-894512 | + | no | 1.6E-08 |
| EN-894656 | + | no | 1.1E-07 |
| EN-895248 | + | no | 2.0E-07 |
| EN-896234 | + | no | 6.9E-06 |
| EN-896373 | + | no | 1.1E-07 |
| EN-897559 | + | no | 4.7E-08 |
| EN-897587 | + | yes | 3.3E-07 |
| EN-898511 | - | no | 3.5E-07 |
| EN-899695 | + | no | 4.4E-07 |
| EN-900406 | - | no | 0.0E+00 |
| EN-900630 | + | no | 4.7E-08 |
| EN-900889 | + | yes | 5.5E-07 |
| EN-902323 | + | no | 3.3E-07 |
| EN-903482 | + | no | 5.7E-07 |
| EN-903564 | + | no | 0.0E+00 |
| EN-903629 | + | no | 1.6E-08 |
| EN-904808 | + | yes | 1.8E-05 |
| EN-906681 | - | no | 0.0E+00 |
| EN-906693 | + | yes | 3.3E-07 |
| EN-908328 | + | no | 4.7E-08 |
| EN-908442 | + | no | 1.7E-07 |
| EN-909135 | + | no | 0.0E+00 |
| EN-909196-99 | + | no | 3.1E-08 |
| EN-909219 | + | no | 3.1E-08 |
| EN-909602 | + | yes | 7.9E-08 |
| EN-910490-92 | + | yes | 4.7E-08 |
| EN-911231 | + | no | 7.9E-08 |
| EN-911799 | - | no | 0.0E+00 |
| EN-912132 | + | no | 3.1E-08 |
| EN-912526 | + | no | 3.3E-07 |
| EN-912683 | + | no | 0.0E+00 |
| EN-912954 | + | no | 1.1E-07 |
| EN-913044 | + | no | 1.1E-07 |
| EN-913755-58 | + | yes | 6.0E-06 |
| EN-913984 | + | no | 0.0E+00 |
| EN-914703 | + | no | 2.4E-07 |
| EN-915226 | + | no | 0.0E+00 |
| EN-915247 | + | no | 1.1E-07 |
| EN-915587 | + | no | 0.0E+00 |
| EN-917221 | - | no | 1.5E-08 |
| EN-917263 | - | no | 0.0E+00 |
| EN-917826-30 | + | yes | 3.1E-08 |
| EN-917847 | + | no | 3.9E-07 |
| EN-919707 | + | yes | 1.1E-07 |
| EN-921685 | + | no | 0.0E+00 |
| EN-921708 | + | yes | 2.4E-07 |
| EN-923956 | - | no | 1.1E-07 |
| EN-924594 | - | yes | 1.1E-06 |
| EN-927634 | - | no | 0.0E+00 |
| EN-928086 | + | yes | 2.8E-07 |
| EN-929084-86 | + | no | 0.0E+00 |
| EN-929841 | - | yes | 0.0E+00 |
| EN-930813 | - | no | 0.0E+00 |
| EN-930837 | + | no | 0.0E+00 |
| EN-931329 | + | no | 2.8E-07 |
| EN-931834 | - | no | 3.5E-07 |
| EN-931846 | + | yes | 3.1E-08 |
| EN-932133 | + | no | 7.9E-08 |
| EN-932575-79 | + | no | 7.9E-08 |
| EN-933430 | - | no | 6.2E-08 |
| EN-934693 | - | no | 0.0E+00 |
| EN-934700 | + | no | 0.0E+00 |
| EN-934711-13 | + | yes | 4.7E-08 |
| EN-934948 | + | yes | 1.2E-06 |
| EN-935853 | - | no | 0.0E+00 |
| EN-936049 | + | yes | 7.9E-07 |
| EN-936443 | - | no | 9.2E-08 |
| EN-936551 | - | no | 0.0E+00 |
| EN-937265 | + | no | 0.0E+00 |
| EN-937968 | - | no | 1.5E-07 |
| EN-938626 | - | no | 0.0E+00 |
| EN-938696 | - | no | 2.3E-07 |
| EN-938785 | - | no | 9.2E-08 |
| EN-939852-54 | - | no | 4.2E-07 |
| EN-940600 | + | yes | 4.7E-08 |
| EN-940789 | + | no | 0.0E+00 |
| EN-941199 | + | no | 1.7E-07 |
| EN-942647 | - | no | 0.0E+00 |
| EN-942781 | + | no | 4.0E-06 |
| EN-943332 | + | no | 0.0E+00 |
| EN-945288-90 | + | yes | 2.0E-07 |
| EN-945606 | + | no | 0.0E+00 |
| EN-945869-71 | + | no | 0.0E+00 |
| EN-945942 | + | no | 2.9E-06 |
| EN-945977-79 | + | no | 1.7E-06 |
| EN-946385 | + | no | 7.9E-08 |
| LE-947536 | + | only | n/a |
| LE-947658 | - | only | n/a |
| EN-947837 | + | no | 0.0E+00 |
| EN-947990 | + | no | 0.0E+00 |
| EN-949758 | + | no | 2.0E-07 |
| EN-951357 | + | yes | 2.2E-06 |
| EN-954916 | - | no | 1.5E-07 |
| EN-955009 | - | no | 1.5E-08 |
| EN-955369-72 | + | yes | 0.0E+00 |
| EN-958606 | + | no | 0.0E+00 |
| EN-959366-68 | + | no | 6.6E-06 |
| EN-960276 | - | yes | 2.0E-07 |
| EN-960421 | - | no | 0.0E+00 |
| EN-961169-71 | + | no | 1.1E-07 |
| EN-962421 | + | no | 5.0E-07 |
| EN-962491 | + | no | 4.7E-08 |
| EN-962714 | + | yes | 2.0E-07 |
| EN-964384 | + | no | 2.0E-07 |
| EN-965032 | + | no | 2.0E-07 |
| EN-965382-87 | + | no | 2.5E-07 |
| EN-966085 | + | no | 1.7E-07 |
| EN-967720 | - | no | 0.0E+00 |
| EN-967743 | + | no | 4.7E-08 |
| EN-968877 | - | no | 2.2E-07 |
| EN-969134 | - | yes | 0.0E+00 |
| EN-969549 | - | no | 0.0E+00 |
| EN-969725 | - | yes | 1.5E-07 |
| LE-969725-30 | - | only | n/a |
| EN-969742 | + | no | 4.7E-08 |
| EN-969814-17 | + | yes | 9.1E-06 |
| EN-970326 | - | no | 2.0E-07 |
| EN-972211 | + | no | 1.6E-08 |
| EN-972287 | + | no | 3.3E-07 |
| EN-974000 | + | no | 7.9E-08 |
| EN-975567 | + | no | 3.1E-08 |
| EN-975724 | + | yes | 1.1E-06 |
| EN-975931 | - | no | 1.5E-07 |
| EN-977085 | + | no | 1.1E-07 |
| EN-977906 | + | no | 3.1E-08 |
| EN-977948 | + | yes | 6.1E-07 |
| EN-978854 | - | no | 1.5E-08 |
| EN-979361 | - | yes | 0.0E+00 |
| EN-979792 | - | no | 0.0E+00 |
| EN-979897-00 | - | no | 4.6E-08 |
| EN-981381 | + | yes | 8.3E-07 |
| EN-981426 | + | no | 0.0E+00 |
| EN-981518 | - | yes | 0.0E+00 |
| EN-981535-37 | + | no | 0.0E+00 |
| EN-982031 | + | no | 0.0E+00 |
| EN-982171 | + | no | 2.8E-07 |
| EN-983828 | + | no | 1.6E-08 |
| EN-984004 | - | no | 2.2E-07 |
| EN-988959 | - | no | 0.0E+00 |
| EN-989811 | - | no | 0.0E+00 |
| EN-989977 | + | yes | 1.1E-07 |
| EN-990209 | - | no | 0.0E+00 |
| EN-990453 | - | yes | 8.9E-06 |
| EN-990787 | - | no | 6.0E-07 |
| EN-990856 | + | no | 3.1E-08 |
| EN-992077 | + | no | 1.6E-08 |
| EN-992329 | + | yes | 2.2E-07 |
| EN-992354 | - | no | 2.3E-07 |
| EN-992750 | - | no | 9.7E-07 |
| EN-993659 | + | no | 5.7E-07 |
| EN-994723 | + | no | 4.7E-08 |
| EN-995977 | - | no | 3.5E-07 |
| EN-996004 | + | no | 4.9E-07 |
| LE-996061 | + | only | n/a |
| EN-996566 | + | yes | 0.0E+00 |
| EN-998385-88 | + | no | 0.0E+00 |
| EN-999947 | - | no | 4.6E-06 |
| EN-999969 | + | no | 3.0E-06 |
| EN-1000145 | - | no | 1.4E-07 |
| EN-1000269 | + | no | 5.2E-07 |
| EN-1000374 | + | yes | 1.6E-08 |
| EN-1000439 | + | no | 0.0E+00 |
| EN-1001013 | + | yes | 0.0E+00 |
| EN-1001025 | + | yes | 0.0E+00 |
| EN-1002663 | + | no | 4.7E-08 |
| EN-1004977 | - | no | 0.0E+00 |
| EN-1006293 | - | yes | 6.2E-08 |
| EN-1007146 | - | no | 0.0E+00 |
| EN-1007227 | - | no | 0.0E+00 |
| EN-1007244 | + | yes | 2.4E-07 |
| EN-1007293 | + | no | 0.0E+00 |
| EN-1007683 | - | no | 9.2E-08 |
| EN-1008557 | + | yes | 1.2E-06 |
| EN-1008574 | + | no | 2.2E-07 |
| EN-1008757 | + | no | 7.9E-08 |
| EN-1009216-19 | + | yes | 0.0E+00 |
| EN-1009613 | - | yes | 4.6E-08 |
| EN-1009662 | + | no | 4.7E-08 |
| EN-1011665 | - | no | 4.6E-08 |
| EN-1011744 | + | no | 4.7E-08 |
| EN-1012400 | + | no | 1.6E-08 |
| EN-1014070 | + | no | 0.0E+00 |
| EN-1014498 | + | no | 7.9E-08 |
| EN-1014566 | - | no | 3.1E-08 |
| EN-1014574 | + | no | 0.0E+00 |
| EN-1014860 | - | no | 1.1E-07 |
| EN-1014897 | + | no | 2.8E-07 |
| EN-1015016 | - | yes | 2.5E-07 |
| EN-1015027 | + | no | 0.0E+00 |
| EN-1015214 | + | no | 2.8E-07 |
| EN-1015510 | - | no | 1.5E-07 |
| EN-1015519 | + | no | 3.6E-07 |
| EN-1015856-63 | - | yes | 0.0E+00 |
| EN-1015874-76 | + | yes | 3.6E-07 |
| EN-1016349 | + | no | 0.0E+00 |
| EN-1016376 | + | no | 7.9E-08 |
| EN-1016469-71 | - | yes | 0.0E+00 |
| EN-1016480 | + | yes | 1.6E-08 |
| EN-1016743 | - | no | 0.0E+00 |
| EN-1017533 | + | no | 0.0E+00 |
| EN-1018154 | - | no | 9.2E-08 |
| EN-1019370 | - | no | 0.0E+00 |
| EN-1021930 | + | yes | 3.6E-07 |
| EN-1022462 | + | yes | 0.0E+00 |
| EN-1022699-02 | + | no | 1.4E-07 |
| EN-1023082-84 | - | yes | 0.0E+00 |
| EN-1024597 | - | no | 6.2E-08 |
| EN-1025409 | - | no | 2.0E-06 |
| EN-1025484 | + | yes | 1.6E-08 |
| EN-1025754 | + | no | 7.9E-08 |
| EN-1026176 | + | no | 1.2E-06 |
| EN-1026758 | - | no | 1.5E-08 |
| EN-1027403 | - | no | 4.9E-07 |
| EN-1027593 | + | no | 4.7E-08 |
| EN-1027725 | - | yes | 8.3E-07 |
| EN-1027779 | - | yes | 9.1E-07 |
| EN-1027918 | + | no | 1.1E-07 |
| EN-1027940 | + | no | 3.0E-07 |
| EN-1027951-53 | + | yes | 9.8E-07 |
| EN-1029184 | - | yes | 6.9E-07 |
| EN-1029267 | + | yes | 2.0E-06 |
| EN-1032555 | - | no | 5.7E-07 |
| EN-1032850 | + | no | 0.0E+00 |
| EN-1033113 | + | no | 2.8E-07 |
| EN-1035874 | - | no | 4.6E-08 |
| EN-1037249 | + | no | 4.7E-06 |
| EN-1037840-44 | - | no | 3.1E-08 |
| EN-1039373 | - | no | 0.0E+00 |
| EN-1039754 | + | no | 9.4E-08 |
| LE-1039814 | + | only | n/a |
| EN-1041521 | + | no | 1.6E-08 |
| EN-1041632 | - | no | 6.2E-08 |
| EN-1041676 | + | no | 1.6E-08 |
| EN-1041864 | + | no | 0.0E+00 |
| EN-1042580 | + | yes | 3.3E-07 |
| EN-1042697 | + | no | 1.6E-08 |
| EN-1042868 | - | no | 3.1E-08 |
| EN-1043168 | + | yes | 0.0E+00 |
| EN-1044734 | + | no | 9.4E-08 |
| EN-1045509-12 | + | no | 0.0E+00 |
| LE-1045871 | - | only | n/a |
| EN-1046567 | + | no | 4.7E-08 |
| EN-1046918 | + | yes | 5.0E-07 |
| EN-1047785 | - | no | 3.1E-08 |
| EN-1048093 | + | no | 0.0E+00 |
| EN-1048841 | + | no | 1.6E-08 |
| EN-1049837 | - | no | 0.0E+00 |
| EN-1049882 | + | yes | 2.8E-07 |
| EN-1050421 | + | no | 2.0E-07 |
| EN-1050686 | + | no | 0.0E+00 |
| EN-1052277 | + | yes | 1.1E-07 |
| EN-1052782 | - | no | 0.0E+00 |
| EN-1052811 | - | no | 2.6E-07 |
| EN-1053598 | + | no | 0.0E+00 |
| EN-1055166 | + | yes | 0.0E+00 |
| EN-1055681 | + | yes | 6.0E-07 |
| EN-1056424-28 | - | no | 0.0E+00 |
| EN-1056786 | + | no | 3.1E-08 |
| EN-1057157 | + | no | 0.0E+00 |
| EN-1058406 | - | no | 0.0E+00 |
| EN-1058573 | - | no | 8.3E-07 |
| LE-1058587 | + | only | n/a |
| LE-1058658 | - | only | n/a |
| EN-1059328 | - | no | 2.2E-05 |
| EN-1059401 | - | no | 1.2E-06 |
| EN-1059780 | - | no | 1.1E-07 |
| EN-1061929 | + | no | 2.4E-07 |
| EN-1062052 | - | yes | 1.8E-07 |
| EN-1063603 | + | yes | 0.0E+00 |
| EN-1065092 | + | no | 0.0E+00 |
| EN-1065866 | + | no | 0.0E+00 |
| EN-1067840 | - | no | 7.8E-07 |
| EN-1068406 | - | no | 6.2E-08 |
| EN-1069188-90 | + | no | 5.5E-07 |
| EN-1069245 | - | yes | 1.7E-07 |
| EN-1069254 | + | no | 1.6E-08 |
| EN-1069347 | + | yes | 1.0E-06 |
| EN-1071349 | - | no | 1.1E-07 |
| EN-1071980 | + | no | 1.6E-08 |
| EN-1072145 | - | yes | 0.0E+00 |
| EN-1072688 | + | no | 4.7E-08 |
| EN-1073355-58 | + | no | 0.0E+00 |
| EN-1073456 | + | no | 1.6E-08 |
| EN-1073600 | + | no | 3.1E-08 |
| EN-1074228 | - | yes | 0.0E+00 |
| EN-1075218 | + | yes | 9.1E-07 |
| EN-1075234 | + | no | 3.1E-08 |
| EN-1075333 | + | no | 1.4E-06 |
| EN-1076257 | + | yes | 1.1E-07 |
| EN-1077509 | + | no | 4.7E-08 |
| EN-1077863 | + | no | 2.5E-07 |
| EN-1078182-84 | + | no | 3.1E-08 |
| EN-1078903-05 | + | yes | 4.3E-06 |
| EN-1080017 | - | no | 9.2E-08 |
| EN-1080350 | + | no | 1.1E-07 |
| EN-1080383 | - | no | 3.1E-08 |
| EN-1080499 | + | yes | 1.7E-07 |
| EN-1080758 | - | no | 1.1E-07 |
| EN-1080841 | + | no | 2.8E-07 |
| EN-1081244 | + | no | 4.7E-08 |
| EN-1081373 | + | no | 1.1E-07 |
| EN-1081853 | + | no | 0.0E+00 |
| EN-1082084 | + | no | 1.6E-08 |
| EN-1083831 | - | yes | 5.2E-06 |
| EN-1084504 | - | yes | 3.1E-08 |
| EN-1084686 | + | no | 0.0E+00 |
| EN-1085798 | + | no | 0.0E+00 |
| EN-1087329 | - | no | 0.0E+00 |
| EN-1087430 | - | no | 5.4E-07 |
| EN-1087470 | - | no | 6.6E-07 |
| EN-1087803 | - | no | 0.0E+00 |
| EN-1088050 | + | no | 9.4E-08 |
| EN-1088763 | + | no | 0.0E+00 |
| EN-1090465 | + | yes | 2.5E-06 |
| EN-1090914 | + | no | 3.1E-08 |
| EN-1091479 | + | no | 2.4E-07 |
| EN-1092046 | + | no | 1.4E-06 |
| EN-1093221 | + | yes | 6.8E-07 |
| EN-1093392 | - | no | 9.2E-08 |
| EN-1093522 | - | no | 1.8E-07 |
| EN-1093578 | + | no | 0.0E+00 |
| EN-1094476 | - | yes | 4.2E-07 |
| EN-1094533 | + | no | 4.7E-08 |
| EN-1094775 | + | yes | 2.2E-05 |
| EN-1094924 | + | no | 0.0E+00 |
| EN-1095086 | + | no | 2.7E-06 |
| EN-1095472 | + | no | 3.1E-08 |
| EN-1097031 | + | no | 3.1E-08 |
| EN-1098796 | + | no | 0.0E+00 |
| EN-1099634 | + | no | 1.3E-05 |
| EN-1101230 | + | no | 4.4E-07 |
| EN-1101306 | + | yes | 0.0E+00 |
| EN-1101309 | - | no | 4.6E-08 |
| EN-1101429 | - | no | 2.0E-07 |
| EN-1101559 | + | no | 0.0E+00 |
| EN-1101807 | - | no | 0.0E+00 |
| EN-1101825 | + | no | 0.0E+00 |
| EN-1101837 | + | no | 1.1E-07 |
| EN-1102005 | + | no | 0.0E+00 |
| EN-1102734 | - | no | 0.0E+00 |
| EN-1102810 | - | no | 1.7E-07 |
| LE-1103186 | - | only | n/a |
| EN-1103281 | + | no | 0.0E+00 |
| EN-1104035 | + | no | 4.7E-08 |
| EN-1104227 | + | no | 1.0E-06 |
| EN-1104240-43 | - | no | 1.8E-06 |
| EN-1104716-18 | - | no | 1.5E-08 |
| EN-1105102 | + | no | 1.4E-07 |
| EN-1105488 | + | no | 0.0E+00 |
| EN-1106831 | + | no | 0.0E+00 |
| EN-1106844 | - | no | 0.0E+00 |
| EN-1106876 | + | no | 2.8E-07 |
| EN-1107431 | + | no | 2.2E-07 |
| EN-1107881 | + | no | 5.0E-07 |
| EN-1108816-18 | + | no | 0.0E+00 |
| EN-1109407 | - | no | 0.0E+00 |
| LE-1109579 | + | only | n/a |
| EN-1109761-67 | + | no | 0.0E+00 |
| EN-1110151-53 | + | yes | 1.1E-07 |
| EN-1110196-99 | + | yes | 0.0E+00 |
| EN-1112490 | + | no | 0.0E+00 |
| EN-1112832 | - | no | 0.0E+00 |
| EN-1113155 | - | no | 0.0E+00 |
| EN-1113420 | - | no | 4.2E-06 |
| EN-1113620-22 | + | yes | 3.1E-08 |
| EN-1113850 | + | no | 2.0E-07 |
| EN-1114037 | - | no | 6.2E-08 |
| EN-1114266 | + | no | 2.8E-07 |
| EN-1114827 | + | yes | 4.7E-08 |
| EN-1114984 | - | no | 3.1E-08 |
| EN-1116070 | + | no | 1.1E-07 |
| EN-1116152 | + | no | 0.0E+00 |
| EN-1116569 | + | no | 0.0E+00 |
| EN-1122325-28 | - | yes | 1.5E-08 |
| EN-1122360 | + | no | 4.7E-08 |
| EN-1122408 | + | yes | 4.7E-08 |
| EN-1122517 | + | yes | 0.0E+00 |
| EN-1122626 | - | no | 6.2E-08 |
| EN-1123902 | - | no | 1.5E-07 |
| EN-1125394 | - | yes | 1.5E-08 |
| EN-1125416 | + | no | 0.0E+00 |
| EN-1126062 | - | no | 0.0E+00 |
| EN-1126836 | - | no | 9.2E-08 |
| EN-1126965 | + | no | 9.4E-08 |
| EN-1127200 | + | no | 0.0E+00 |
| EN-1128954 | - | yes | 0.0E+00 |
| EN-1129091-94 | + | yes | 2.0E-07 |
| EN-1129299 | - | no | 0.0E+00 |
| EN-1131717 | + | yes | 1.0E-06 |
| EN-1131937 | - | no | 1.5E-08 |
| EN-1132614-21 | - | yes | 1.1E-05 |
| EN-1132644 | + | yes | 5.7E-07 |
| EN-1132729 | + | no | 2.5E-06 |
| EN-1135006 | - | no | 0.0E+00 |
| EN-1135034-36 | - | no | 1.2E-07 |
| EN-1135417 | + | no | 1.6E-06 |
| EN-1135750 | + | no | 2.8E-07 |
| EN-1136185 | - | no | 3.1E-08 |
| EN-1136509 | + | yes | 0.0E+00 |
| EN-1136612 | + | no | 1.6E-08 |
| EN-1137103 | + | no | 9.4E-08 |
| EN-1137491 | - | no | 1.1E-07 |
| EN-1137500 | + | no | 0.0E+00 |
| EN-1139286 | + | yes | 1.6E-08 |
| EN-1139696 | + | no | 1.6E-08 |
| EN-1141344 | + | no | 0.0E+00 |
| EN-1141591 | + | no | 4.4E-07 |
| EN-1141827 | - | yes | 7.8E-07 |
| EN-1142361-63 | + | no | 1.6E-08 |
| EN-1142945 | - | no | 0.0E+00 |
| EN-1144415 | + | no | 4.2E-07 |
| EN-1144766 | + | no | 4.5E-07 |
| EN-1145429-31 | - | yes | 5.9E-06 |
| EN-1145521 | + | yes | 1.6E-08 |
| EN-1146258 | - | yes | 1.0E-06 |
| EN-1146264 | + | no | 1.1E-07 |
| EN-1146302-05 | + | no | 9.0E-06 |
| EN-1147101 | + | no | 1.7E-07 |
| EN-1147203 | - | no | 0.0E+00 |
| EN-1147290 | + | yes | 3.6E-06 |
| EN-1149864 | + | yes | 0.0E+00 |
| EN-1149891 | + | no | 0.0E+00 |
| EN-1149947 | - | yes | 2.0E-07 |
| EN-1150080 | + | no | 3.5E-06 |
| EN-1150620 | + | no | 1.4E-07 |
| EN-1151241 | - | yes | 0.0E+00 |
| EN-1151914 | + | no | 1.6E-08 |
| EN-1152133-36 | + | yes | 2.2E-06 |
| EN-1152247 | + | no | 0.0E+00 |
| EN-1152424 | - | no | 9.2E-08 |
| EN-1152886-90 | + | no | 1.6E-08 |
| EN-1153537-39 | + | no | 0.0E+00 |
| EN-1154536 | + | yes | 4.7E-08 |
| EN-1154611 | + | no | 1.0E-06 |
| EN-1154728 | - | no | 9.2E-08 |
| EN-1155532 | + | no | 0.0E+00 |
| EN-1155862 | + | no | 0.0E+00 |
| EN-1156456-60 | + | no | 1.6E-08 |
| EN-1156689 | - | no | 4.6E-08 |
| EN-1156708 | + | no | 0.0E+00 |
| EN-1156806 | - | no | 6.0E-07 |
| EN-1157597 | + | no | 0.0E+00 |
| EN-1159093 | + | no | 2.4E-07 |
| EN-1159185 | + | no | 4.7E-08 |
| EN-1160415 | - | yes | 2.0E-07 |
| EN-1160528 | + | no | 3.0E-06 |
| EN-1161869-71 | + | no | 1.1E-07 |
| EN-1162252 | - | yes | 0.0E+00 |
| EN-1163120 | + | no | 0.0E+00 |
| EN-1163170 | - | yes | 0.0E+00 |
| EN-1163354 | + | no | 3.1E-08 |
| EN-1164188 | - | no | 0.0E+00 |
| EN-1164601 | - | no | 0.0E+00 |
| EN-1164654 | - | no | 2.9E-07 |
| EN-1166294-96 | + | no | 3.3E-07 |
| EN-1167481 | - | yes | 1.5E-07 |
| EN-1167501 | + | no | 0.0E+00 |
| EN-1167796-98 | + | yes | 1.1E-07 |
| EN-1168453 | + | no | 9.4E-08 |
| EN-1168668 | + | no | 0.0E+00 |
| EN-1168968-71 | + | no | 0.0E+00 |
| EN-1169338 | - | no | 1.8E-07 |
| EN-1169379 | + | no | 0.0E+00 |
| EN-1171177 | + | no | 9.4E-08 |
| EN-1171275 | + | yes | 0.0E+00 |
| EN-1171790 | - | no | 2.0E-07 |
| EN-1173247 | + | no | 1.1E-07 |
| EN-1173307-10 | + | no | 4.7E-08 |
| EN-1174606 | + | no | 1.6E-08 |
| EN-1175121 | + | no | 0.0E+00 |
| EN-1175552-54 | + | yes | 0.0E+00 |
| EN-1176025 | + | no | 1.4E-06 |
| EN-1176099 | - | no | 0.0E+00 |
| EN-1176775 | - | no | 0.0E+00 |
| EN-1176880 | + | no | 0.0E+00 |
| EN-1177039 | + | no | 0.0E+00 |
| EN-1177164 | + | yes | 1.7E-05 |
| EN-1177237 | + | no | 2.2E-07 |
| EN-1177600 | + | no | 1.6E-08 |
| EN-1178875 | + | no | 3.1E-08 |
| EN-1178913 | + | no | 1.1E-07 |
| EN-1179339 | - | no | 7.8E-07 |
| EN-1179391 | - | no | 3.1E-08 |
| EN-1179415 | + | yes | 2.4E-07 |
| EN-1179823 | + | no | 2.4E-07 |
| EN-1180257 | + | no | 0.0E+00 |
| EN-1181472 | + | yes | 0.0E+00 |
| EN-1184210-12 | - | yes | 0.0E+00 |
| EN-1184220 | - | yes | 2.5E-06 |
| EN-1184226 | - | yes | 2.0E-07 |
| EN-1184312 | + | no | 8.7E-07 |
| EN-1185378 | + | no | 1.4E-07 |
| EN-1187111 | - | no | 5.8E-07 |
| EN-1188177-79 | + | no | 1.7E-07 |
| EN-1188598 | - | no | 3.1E-08 |
| EN-1189068 | + | yes | 4.7E-08 |
| EN-1189369 | - | no | 3.5E-07 |
| EN-1189643 | - | no | 6.6E-06 |
| EN-1190166 | - | no | 2.5E-07 |
| EN-1190265 | + | yes | 0.0E+00 |
| EN-1190316 | + | no | 0.0E+00 |
| EN-1190361 | - | no | 2.0E-07 |
| EN-1190638 | + | no | 2.8E-07 |
| EN-1190640 | - | no | 0.0E+00 |
| EN-1191287 | + | yes | 0.0E+00 |
| EN-1191455-57 | - | yes | 1.8E-07 |
| EN-1191960 | - | no | 1.1E-07 |
| EN-1192025-28 | - | no | 6.2E-08 |
| EN-1192044 | + | no | 9.4E-08 |
| EN-1193109 | - | no | 6.2E-08 |
| EN-1193404 | - | yes | 3.1E-08 |
| EN-1193502 | + | yes | 9.4E-06 |
| EN-1193844 | + | no | 4.7E-08 |
| EN-1194132 | + | no | 6.0E-07 |
| EN-1195050 | + | yes | 0.0E+00 |
| EN-1195633 | - | yes | 0.0E+00 |
| EN-1195963 | + | no | 3.3E-07 |
| EN-1196203 | - | yes | 1.5E-07 |
| EN-1196591 | - | no | 2.3E-07 |
| EN-1196911 | - | no | 2.0E-07 |
| EN-1198468 | - | yes | 1.1E-06 |
| LE-1198600 | + | only | n/a |
| EN-1199143 | + | no | 0.0E+00 |
| EN-1199737 | + | no | 0.0E+00 |
| EN-1199937-40 | + | no | 0.0E+00 |
| EN-1200117 | + | no | 0.0E+00 |
| EN-1202004 | - | yes | 6.2E-08 |
| EN-1204115 | - | no | 1.1E-07 |
| EN-1204372 | + | no | 7.9E-08 |
| EN-1205150-52 | + | no | 0.0E+00 |
| LE-1205160 | - | only | n/a |
| EN-1205284 | + | no | 0.0E+00 |
| EN-1205700 | - | no | 0.0E+00 |
| EN-1206179 | - | no | 1.8E-06 |
| EN-1206430-32 | - | no | 5.3E-06 |
| EN-1206450 | + | no | 2.0E-07 |
| EN-1207177 | - | no | 1.5E-07 |
| EN-1207467-69 | + | yes | 0.0E+00 |
| EN-1208289 | + | no | 4.7E-08 |
| EN-1208754 | - | no | 1.5E-08 |
| EN-1208891 | - | no | 2.3E-07 |
| EN-1211829-31 | + | yes | 0.0E+00 |
| EN-1213093 | - | no | 4.6E-08 |
| EN-1213305-07 | + | no | 0.0E+00 |
| EN-1213559 | + | no | 3.1E-08 |
| EN-1214043 | - | no | 4.2E-07 |
| EN-1214874 | + | no | 1.0E-06 |
| EN-1215815 | + | no | 1.0E-06 |
| EN-1215829 | - | no | 1.2E-07 |
| EN-1216814 | - | no | 1.7E-07 |
| EN-1217030-33 | - | yes | 1.5E-08 |
| EN-1217042 | - | yes | 0.0E+00 |
| EN-1217095 | - | no | 9.2E-08 |
| EN-1217134 | - | no | 0.0E+00 |
| EN-1218041 | + | no | 1.7E-07 |
| EN-1219057 | + | no | 0.0E+00 |
| EN-1219262 | + | no | 0.0E+00 |
| EN-1219452 | + | no | 4.7E-08 |
| EN-1219645 | + | no | 0.0E+00 |
| EN-1220130 | - | no | 4.8E-07 |
| EN-1220499 | + | yes | 2.0E-07 |
| EN-1220637 | - | no | 0.0E+00 |
| EN-1220739 | - | yes | 0.0E+00 |
| EN-1221426 | + | no | 1.4E-07 |
| EN-1222224 | + | no | 4.7E-08 |
| EN-1224767 | - | no | 0.0E+00 |
| EN-1224843 | + | yes | 2.4E-07 |
| EN-1224983-85 | - | no | 1.7E-07 |
| EN-1225162 | + | no | 0.0E+00 |
| EN-1225736 | + | no | 9.4E-08 |
| EN-1226189 | - | yes | 4.6E-08 |
| EN-1227206 | + | no | 4.7E-08 |
| EN-1227454 | - | yes | 0.0E+00 |
| EN-1228004 | + | no | 2.7E-07 |
| EN-1229215 | - | no | 6.2E-08 |
| EN-1229837 | + | no | 3.1E-06 |
| EN-1231415 | - | no | 1.1E-07 |
| EN-1232285 | - | no | 1.8E-07 |
| EN-1232568 | - | no | 0.0E+00 |
| EN-1234190 | + | no | 0.0E+00 |
| EN-1234794 | + | no | 0.0E+00 |
| EN-1235177 | + | no | 0.0E+00 |
| EN-1235187 | + | no | 0.0E+00 |
| EN-1235587-89 | - | no | 0.0E+00 |
| EN-1235854 | - | yes | 0.0E+00 |
| EN-1235868-75 | + | no | 1.1E-07 |
| EN-1236117 | + | no | 1.2E-06 |
| EN-1236435 | + | no | 4.7E-08 |
| EN-1236506 | - | no | 2.0E-07 |
| EN-1236614 | - | no | 1.8E-07 |
| EN-1237851 | + | no | 1.2E-06 |
| EN-1238418 | + | no | 0.0E+00 |
| EN-1238489 | - | no | 0.0E+00 |
| EN-1238854 | - | no | 1.1E-07 |
| EN-1239122 | - | no | 0.0E+00 |
| EN-1239232-34 | + | no | 5.7E-07 |
| EN-1239376 | - | yes | 1.1E-07 |
| EN-1239922 | + | no | 0.0E+00 |
| EN-1240258 | - | no | 4.8E-07 |
| EN-1240306 | + | yes | 3.3E-07 |
| EN-1240497 | - | no | 2.5E-07 |
| EN-1241133 | - | no | 3.1E-08 |
| EN-1241356 | + | no | 0.0E+00 |
| EN-1243835 | + | no | 2.4E-07 |
| EN-1244176 | + | yes | 6.7E-06 |
| EN-1244200 | - | no | 0.0E+00 |
| EN-1244221 | + | no | 1.6E-08 |
| EN-1246503 | + | no | 2.2E-07 |
| EN-1247119 | - | yes | 6.2E-08 |
| LE-1247405 | - | only | n/a |
| EN-1247558 | + | no | 4.7E-08 |
| EN-1248551 | - | no | 0.0E+00 |
| EN-1248645 | + | yes | 0.0E+00 |
| EN-1248692 | + | no | 1.6E-08 |
| EN-1249105 | + | no | 0.0E+00 |
| EN-1249452 | + | yes | 1.7E-07 |
| EN-1251316-19 | - | yes | 2.0E-07 |
| EN-1251563 | - | no | 6.9E-07 |
| EN-1251652 | - | no | 6.6E-07 |
| EN-1251721 | + | no | 2.4E-07 |
| EN-1252214 | - | no | 2.0E-07 |
| EN-1252224 | + | no | 7.9E-08 |
| EN-1252609 | + | no | 3.3E-07 |
| EN-1253118 | + | no | 8.1E-06 |
| EN-1253641 | + | no | 4.0E-06 |
| EN-1254532 | - | no | 2.0E-07 |
| EN-1255006 | - | no | 4.9E-07 |
| EN-1255057 | - | yes | 6.8E-07 |
| EN-1255659 | + | no | 6.8E-06 |
| EN-1255780 | + | no | 0.0E+00 |
| EN-1256251 | + | no | 2.0E-07 |
| EN-1258174 | - | no | 6.2E-08 |
| EN-1258196-99 | + | yes | 4.7E-08 |
| EN-1259184 | + | no | 2.7E-07 |
| EN-1259973 | - | no | 0.0E+00 |
| EN-1261165 | + | no | 7.1E-07 |
| EN-1261373 | + | no | 3.6E-06 |
| EN-1261931 | + | no | 1.6E-08 |
| EN-1262713 | + | no | 0.0E+00 |
| EN-1262755 | + | no | 1.1E-07 |
| EN-1262978 | + | no | 5.0E-07 |
| EN-1263179-86 | + | no | 0.0E+00 |
| EN-1263849 | + | no | 4.7E-08 |
| EN-1266473 | + | no | 5.7E-07 |
| EN-1266515 | + | no | 2.8E-07 |
| EN-1268092 | - | no | 1.1E-07 |
| EN-1268418 | + | no | 0.0E+00 |
| EN-1269885 | - | no | 1.5E-07 |
| EN-1270130 | - | yes | 1.5E-07 |
| EN-1270161 | + | no | 3.3E-07 |
| EN-1272846 | - | no | 2.0E-07 |
| EN-1273272-74 | - | no | 0.0E+00 |
| EN-1273602 | - | yes | 7.7E-06 |
| EN-1275663 | + | no | 9.4E-08 |
| EN-1276482 | + | no | 3.0E-06 |
| EN-1277328 | + | no | 0.0E+00 |
| EN-1277397 | - | no | 4.6E-08 |
| EN-1278069 | - | no | 0.0E+00 |
| LE-1278152 | + | only | n/a |
| EN-1278559 | - | no | 6.2E-08 |
| EN-1278863 | + | no | 1.1E-07 |
| EN-1279925 | + | no | 0.0E+00 |
| EN-1279979 | + | no | 1.1E-07 |
| LE-1280121 | + | only | n/a |
| EN-1280790 | - | no | 0.0E+00 |
| EN-1282092 | + | no | 1.2E-06 |
| EN-1282914 | + | no | 4.7E-08 |
| EN-1283314 | + | no | 1.6E-08 |
| EN-1283349 | - | no | 0.0E+00 |
| EN-1284015 | + | yes | 2.4E-07 |
| EN-1284213 | - | no | 1.7E-07 |
| EN-1284849 | + | no | 0.0E+00 |
| EN-1285106-08 | - | no | 1.1E-07 |
| EN-1285989 | - | no | 4.2E-07 |
| EN-1286370 | + | no | 5.5E-07 |
| EN-1287179 | + | no | 1.1E-07 |
| EN-1287868 | - | no | 7.8E-07 |
| EN-1287910 | - | no | 1.1E-07 |
| EN-1289196 | + | no | 2.0E-06 |
| EN-1289206-09 | + | no | 3.1E-08 |
| EN-1290383 | - | no | 0.0E+00 |
| EN-1291164 | + | no | 2.2E-06 |
| EN-1291642 | - | no | 1.7E-07 |
| EN-1292361 | - | no | 0.0E+00 |
| EN-1292698 | - | no | 0.0E+00 |
| EN-1292721 | + | no | 9.4E-08 |
| EN-1293071 | + | no | 9.4E-08 |
| EN-1293339-43 | - | no | 1.4E-06 |
| EN-1293414 | + | no | 0.0E+00 |
| LE-1293706 | - | only | n/a |
| EN-1293943 | + | no | 0.0E+00 |
| EN-1294216 | - | no | 2.0E-07 |
| EN-1294422 | + | yes | 1.6E-08 |
| EN-1296314 | + | no | 6.6E-06 |
| EN-1296906-09 | + | no | 0.0E+00 |
| EN-1297687 | - | no | 9.2E-08 |
| EN-1297696 | + | no | 1.6E-08 |
| EN-1298033 | - | no | 2.0E-07 |
| EN-1298914 | - | no | 0.0E+00 |
| EN-1299807 | + | no | 6.0E-07 |
| EN-1300332 | + | no | 4.7E-08 |
| EN-1300425 | - | no | 1.5E-08 |
| EN-1301835 | + | no | 1.6E-08 |
| EN-1303268 | + | no | 0.0E+00 |
| EN-1303970 | - | no | 4.6E-08 |
| EN-1304216-18 | + | yes | 1.5E-06 |
| EN-1305152 | + | no | 3.1E-08 |
| EN-1305187 | - | no | 0.0E+00 |
| EN-1305288 | - | no | 0.0E+00 |
| EN-1305767 | - | no | 0.0E+00 |
| EN-1305972 | - | no | 2.3E-07 |
| EN-1306065 | - | no | 3.1E-08 |
| EN-1306338 | - | no | 6.8E-07 |
| EN-1306372 | + | no | 1.6E-08 |
| EN-1306863 | - | no | 2.3E-07 |
| EN-1307083-85 | - | no | 3.1E-08 |
| EN-1307882 | - | no | 0.0E+00 |
| EN-1307946 | - | yes | 0.0E+00 |
| EN-1308196 | + | yes | 0.0E+00 |
| EN-1308408 | - | no | 1.2E-07 |
| EN-1308587 | + | no | 1.1E-07 |
| EN-1309200 | - | no | 1.5E-08 |
| LE-1309609 | + | only | n/a |
| EN-1309685 | + | no | 0.0E+00 |
| EN-1309999 | - | no | 1.5E-08 |
| EN-1310571-73 | - | no | 3.4E-07 |
| EN-1310881 | - | no | 2.3E-07 |
| EN-1311312-14 | + | no | 4.7E-08 |
| EN-1311495 | + | no | 3.3E-07 |
| EN-1313784 | + | no | 0.0E+00 |
| EN-1313888 | + | no | 4.7E-08 |
| EN-1314126 | + | no | 0.0E+00 |
| EN-1314224 | - | yes | 0.0E+00 |
| EN-1314280-84 | + | yes | 1.6E-08 |
| EN-1314845 | + | no | 0.0E+00 |
| EN-1315151 | - | no | 6.2E-08 |
| EN-1315248 | + | no | 3.1E-08 |
| EN-1315993 | + | no | 6.5E-07 |
| EN-1316002 | + | no | 0.0E+00 |
| EN-1316299-01 | + | no | 1.1E-05 |
| EN-1316940 | + | no | 5.8E-07 |
| EN-1317362 | + | no | 0.0E+00 |
| EN-1317417 | + | no | 0.0E+00 |
| EN-1317682 | + | no | 0.0E+00 |
| EN-1318165 | + | no | 3.1E-08 |
| EN-1318964 | - | no | 0.0E+00 |
| EN-1319341 | - | no | 0.0E+00 |
| EN-1319977 | + | no | 0.0E+00 |
| EN-1320030 | + | no | 1.1E-07 |
| EN-1320579 | - | no | 2.0E-07 |
| EN-1323382 | - | no | 0.0E+00 |
| EN-1324833 | - | no | 4.6E-08 |
| EN-1324985 | + | no | 0.0E+00 |
| EN-1325267 | - | yes | 9.2E-08 |
| EN-1327053 | - | no | 1.5E-08 |
| EN-1327068 | + | no | 7.9E-08 |
| EN-1327140-47 | + | no | 4.4E-07 |
| EN-1328102-04 | - | no | 9.2E-08 |
| EN-1328119 | + | no | 4.7E-08 |
| EN-1329354 | - | no | 6.2E-08 |
| EN-1329373 | + | no | 4.7E-08 |
| EN-1330262 | - | yes | 3.5E-06 |
| EN-1330399 | + | no | 1.6E-08 |
| EN-1330494-96 | + | no | 0.0E+00 |
| EN-1331064-66 | - | no | 1.4E-07 |
| LE-1331678 | - | only | n/a |
| EN-1331724 | - | no | 4.6E-08 |
| EN-1332587 | - | yes | 6.8E-07 |
| EN-1332637-42 | + | yes | 1.1E-07 |
| EN-1332650 | + | no | 0.0E+00 |
| EN-1334757-60 | - | no | 0.0E+00 |
| EN-1334794 | - | no | 6.2E-08 |
| EN-1335012 | - | yes | 5.2E-05 |
| EN-1335132 | + | yes | 9.4E-08 |
| EN-1335849 | + | yes | 0.0E+00 |
| EN-1336420 | + | no | 0.0E+00 |
| EN-1336601 | + | no | 1.7E-07 |
| EN-1338702 | - | no | 9.2E-07 |
| EN-1338937 | - | yes | 8.3E-07 |
| EN-1338954 | + | no | 4.1E-07 |
| EN-1339319 | + | no | 0.0E+00 |
| EN-1339643 | - | no | 3.1E-08 |
| EN-1339919 | - | yes | 1.1E-07 |
| EN-1340639 | - | no | 9.2E-08 |
| EN-1340715 | - | no | 1.8E-07 |
| EN-1340773 | - | no | 2.3E-07 |
| EN-1340951 | - | no | 1.1E-07 |
| EN-1341038 | - | yes | 0.0E+00 |
| EN-1341806 | - | yes | 6.4E-06 |
| EN-1341909 | - | no | 0.0E+00 |
| EN-1342549 | - | no | 9.5E-07 |
| EN-1343070 | + | yes | 4.7E-08 |
| EN-1343790 | - | no | 1.5E-07 |
| EN-1343890 | + | no | 3.1E-08 |
| EN-1344405 | - | no | 1.5E-07 |
| EN-1344991 | - | no | 0.0E+00 |
| EN-1345069 | - | no | 1.5E-07 |
| EN-1345306 | - | no | 7.7E-08 |
| EN-1347597 | + | no | 0.0E+00 |
| EN-1347989 | + | no | 1.1E-07 |
| EN-1349482 | - | no | 4.6E-08 |
| EN-1349491 | - | no | 6.9E-07 |
| EN-1351007 | - | no | 0.0E+00 |
| EN-1351314-17 | + | yes | 6.7E-06 |
| EN-1351869 | - | no | 1.5E-07 |
| EN-1352430 | - | yes | 1.2E-06 |
| EN-1352447-49 | + | yes | 0.0E+00 |
| EN-1352848 | - | no | 0.0E+00 |
| EN-1352867 | + | no | 1.6E-08 |
| EN-1353075 | - | yes | 0.0E+00 |
| EN-1354845 | - | no | 4.6E-08 |
| EN-1355091 | - | yes | 3.5E-07 |
| EN-1357443 | - | no | 0.0E+00 |
| EN-1357838 | - | no | 6.2E-08 |
| EN-1358060 | + | no | 2.8E-07 |
| EN-1358798 | + | no | 3.1E-08 |
| LE-1359646 | - | only | n/a |
| EN-1359664 | + | no | 0.0E+00 |
| EN-1359901 | + | no | 0.0E+00 |
| EN-1360471 | - | no | 0.0E+00 |
| EN-1360687 | + | no | 3.6E-07 |
| EN-1361494 | - | no | 0.0E+00 |
| EN-1363380 | - | no | 0.0E+00 |
| EN-1363392 | + | no | 0.0E+00 |
| EN-1363449 | - | no | 2.0E-07 |
| EN-1363511 | - | no | 6.9E-07 |
| EN-1364491 | - | yes | 6.9E-07 |
| EN-1364587 | + | yes | 4.4E-07 |
| EN-1364736-39 | + | no | 2.8E-07 |
| EN-1365427 | - | no | 0.0E+00 |
| EN-1365691-93 | - | yes | 0.0E+00 |
| EN-1366145 | + | yes | 1.6E-08 |
| EN-1368351 | - | no | 0.0E+00 |
| EN-1368737 | - | no | 2.0E-07 |
| EN-1368882 | - | no | 6.2E-08 |
| EN-1370018 | + | no | 6.1E-06 |
| EN-1370506 | - | no | 1.1E-07 |
| EN-1370675 | - | no | 3.1E-06 |
| EN-1370692 | + | yes | 1.3E-07 |
| EN-1370772 | + | no | 1.6E-08 |
| EN-1370822 | + | no | 4.9E-06 |
| EN-1371448 | - | no | 0.0E+00 |
| EN-1372686 | - | no | 9.2E-08 |
| EN-1372793-96 | + | yes | 6.2E-06 |
| EN-1373154 | - | no | 1.5E-07 |
| EN-1373258-60 | - | no | 0.0E+00 |
| EN-1373997 | - | no | 2.3E-07 |
| EN-1374080 | - | no | 9.2E-08 |
| EN-1374093-95 | - | yes | 6.2E-08 |
| EN-1374116 | + | yes | 0.0E+00 |
| EN-1374133 | - | no | 6.2E-08 |
| EN-1374192 | + | yes | 7.1E-07 |
| EN-1374210 | + | no | 2.5E-06 |
| EN-1376348 | + | no | 1.6E-08 |
| EN-1376381 | - | no | 0.0E+00 |
| EN-1376396 | + | no | 1.6E-08 |
| EN-1377440 | - | yes | 2.2E-06 |
| EN-1378348 | - | yes | 9.2E-08 |
| EN-1378538 | + | yes | 0.0E+00 |
| EN-1379943 | + | no | 4.7E-08 |
| EN-1380561 | - | yes | 2.3E-07 |
| EN-1381455 | - | no | 2.3E-07 |
| EN-1382316 | - | no | 0.0E+00 |
| EN-1383545 | + | no | 2.5E-07 |
| EN-1384206-15 | + | no | 3.1E-06 |
| EN-1384211 | - | no | 9.2E-08 |
| EN-1385419 | - | no | 1.5E-08 |
| EN-1385507 | - | yes | 1.5E-07 |
| EN-1385584 | + | no | 3.1E-08 |
| EN-1385648 | + | yes | 4.5E-06 |
| EN-1386448 | - | no | 2.3E-07 |
| LE-1386466 | + | only | n/a |
| EN-1386577 | + | no | 2.8E-07 |
| EN-1386592 | + | no | 4.7E-08 |
| EN-1386923-26 | + | no | 0.0E+00 |
| LE-1387006 | + | only | n/a |
| EN-1387025 | + | no | 7.9E-08 |
| EN-1387327-30 | + | yes | 2.5E-07 |
| EN-1387545 | + | no | 4.7E-08 |
| EN-1388706 | + | no | 0.0E+00 |
| EN-1389551 | - | yes | 0.0E+00 |
| EN-1390839 | - | yes | 2.0E-07 |
| EN-1390855 | + | no | 1.6E-08 |
| EN-1391383 | - | no | 9.2E-08 |
| EN-1395254 | - | no | 2.3E-07 |
| EN-1396962 | + | no | 4.7E-07 |
| EN-1397373 | + | yes | 0.0E+00 |
| EN-1397588 | - | no | 1.2E-06 |
| EN-1404430 | + | no | 3.1E-08 |
| EN-1405422 | - | yes | 9.2E-08 |
| EN-1405593 | - | no | 0.0E+00 |
| EN-1407263 | - | no | 4.6E-08 |
| EN-1411220 | - | yes | 2.0E-07 |
| EN-1411614 | - | no | 0.0E+00 |
| EN-1412016-18 | + | no | 4.7E-08 |
| EN-1412364-67 | - | no | 6.9E-07 |
| EN-1412528 | - | yes | 0.0E+00 |
| EN-1413035 | + | no | 0.0E+00 |
| EN-1414556 | - | yes | 1.0E-06 |
| EN-1414559 | + | no | 1.1E-07 |
| EN-1415720-22 | - | no | 2.0E-07 |
| EN-1415900 | - | no | 1.1E-07 |
| EN-1416262 | - | no | 4.6E-08 |
| EN-1416749 | - | no | 1.2E-07 |
| EN-1417207 | - | yes | 4.8E-07 |
| LE-1417272 | + | only | n/a |
| EN-1417432 | + | no | 2.0E-07 |
| EN-1418112 | + | no | 0.0E+00 |
| EN-1418875 | - | no | 3.1E-08 |
| EN-1419206 | - | no | 6.2E-08 |
| EN-1419266 | - | no | 2.3E-07 |
| EN-1420273 | + | no | 9.4E-08 |
| EN-1420413 | + | no | 4.4E-07 |
| EN-1420840 | - | no | 6.9E-07 |
| EN-1422393 | - | yes | 2.5E-07 |
| EN-1422513 | + | yes | 1.4E-06 |
| EN-1423111 | + | no | 0.0E+00 |
| EN-1423923 | - | no | 2.0E-07 |
| EN-1424215 | + | no | 0.0E+00 |
| EN-1426665 | - | no | 2.0E-07 |
| EN-1426711 | - | no | 0.0E+00 |
| EN-1426902 | - | no | 1.1E-06 |
| EN-1427115 | - | no | 3.1E-08 |
| EN-1427143 | - | no | 1.1E-07 |
| EN-1427517 | + | no | 0.0E+00 |
| EN-1427773 | + | no | 2.0E-07 |
| EN-1428752 | - | no | 2.0E-07 |
| EN-1429012-17 | - | no | 9.4E-07 |
| EN-1429115 | - | no | 0.0E+00 |
| EN-1430423 | - | no | 1.2E-07 |
| EN-1430979 | + | no | 7.9E-08 |
| EN-1431752 | - | no | 4.8E-07 |
| EN-1432488 | - | no | 0.0E+00 |
| EN-1432837 | - | no | 2.0E-07 |
| EN-1432850 | + | no | 0.0E+00 |
| EN-1433954 | + | no | 0.0E+00 |
| EN-1434185 | + | no | 0.0E+00 |
| EN-1436714 | + | no | 0.0E+00 |
| EN-1438022 | - | no | 6.2E-08 |
| EN-1438180 | - | yes | 1.5E-07 |
| EN-1438918-21 | + | no | 0.0E+00 |
| EN-1439333 | + | yes | 1.7E-07 |
| EN-1439354 | + | no | 1.3E-07 |
| EN-1439955 | - | no | 3.1E-08 |
| EN-1440168 | - | no | 4.6E-08 |
| EN-1440273 | - | yes | 9.4E-07 |
| EN-1442058 | + | no | 2.4E-07 |
| EN-1442220 | + | no | 0.0E+00 |
| EN-1442591 | + | no | 7.9E-08 |
| EN-1443466 | + | no | 4.4E-07 |
| EN-1443699 | - | no | 0.0E+00 |
| LE-1444914 | - | only | n/a |
| EN-1446432 | + | no | 8.3E-07 |
| EN-1448290 | - | no | 2.5E-06 |
| EN-1448392 | - | no | 1.5E-08 |
| EN-1449360 | - | no | 1.5E-07 |
| LE-1449690 | - | only | n/a |
| EN-1450459 | - | yes | 0.0E+00 |
| EN-1450538 | - | no | 3.1E-08 |
| EN-1450552 | + | no | 0.0E+00 |
| EN-1450909 | + | no | 1.6E-08 |
| EN-1451701 | - | no | 0.0E+00 |
| EN-1454943 | + | yes | 4.7E-08 |
| EN-1455042 | - | no | 2.3E-06 |
| EN-1455083 | - | yes | 0.0E+00 |
| EN-1455297 | - | no | 0.0E+00 |
| EN-1455306 | - | no | 3.1E-08 |
| EN-1455324 | - | no | 0.0E+00 |
| EN-1455351 | - | no | 9.2E-07 |
| EN-1455499 | - | no | 0.0E+00 |
| EN-1455552-54 | + | no | 1.1E-07 |
| EN-1455988 | - | yes | 1.5E-08 |
| EN-1456006 | + | no | 0.0E+00 |
| EN-1456065-67 | - | no | 0.0E+00 |
| EN-1456214 | - | yes | 8.8E-07 |
| EN-1456921 | - | no | 9.2E-08 |
| EN-1457499 | + | no | 0.0E+00 |
| EN-1458183 | + | no | 0.0E+00 |
| EN-1458480 | - | no | 0.0E+00 |
| EN-1458566 | + | no | 1.1E-06 |
| EN-1458807 | - | no | 1.5E-08 |
| EN-1458876 | - | no | 9.2E-08 |
| EN-1459063 | - | no | 3.1E-08 |
| EN-1460016 | - | no | 6.3E-07 |
| EN-1460352 | - | no | 0.0E+00 |
| EN-1460892 | - | no | 2.5E-07 |
| EN-1460911 | + | no | 0.0E+00 |
| EN-1461548-50 | - | yes | 1.5E-08 |
| LE-1462905-07 | - | only | n/a |
| EN-1463970 | + | no | 0.0E+00 |
| EN-1464666 | - | no | 3.1E-08 |
| EN-1465049 | + | no | 1.6E-08 |
| EN-1466701 | + | no | 3.1E-08 |
| EN-1466833-35 | - | no | 0.0E+00 |
| EN-1466859 | - | no | 8.9E-07 |
| EN-1466921 | - | no | 4.6E-08 |
| EN-1467258 | - | no | 4.6E-08 |
| EN-1467291 | - | no | 3.1E-08 |
| EN-1467703 | - | no | 9.5E-07 |
| EN-1467931-34 | - | yes | 9.2E-08 |
| EN-1467961 | - | no | 6.3E-07 |
| EN-1468406 | - | no | 0.0E+00 |
| LE-1468550 | - | only | n/a |
| EN-1468757 | - | no | 1.1E-07 |
| EN-1469904 | + | no | 0.0E+00 |
| EN-1470651 | - | no | 1.5E-08 |
| EN-1474164 | - | no | 0.0E+00 |
| EN-1474724 | - | yes | 1.2E-05 |
| EN-1475347 | - | no | 6.9E-07 |
| EN-1475358 | - | no | 3.1E-08 |
| EN-1475370 | + | no | 4.7E-08 |
| EN-1475487 | - | no | 1.1E-06 |
| EN-1476440 | - | no | 2.6E-07 |
| EN-1476952 | - | no | 1.5E-07 |
| EN-1477062 | - | no | 1.5E-08 |
| EN-1478097 | - | yes | 6.2E-08 |
| EN-1478135 | - | no | 2.3E-07 |
| EN-1478192-95 | + | yes | 3.1E-08 |
| EN-1478337 | - | no | 1.5E-08 |
| EN-1478354 | - | no | 0.0E+00 |
| EN-1478377 | + | no | 1.7E-07 |
| EN-1478489 | - | no | 0.0E+00 |
| EN-1478754 | - | no | 1.7E-06 |
| EN-1478821 | - | no | 1.5E-08 |
| EN-1478835-38 | + | no | 9.4E-08 |
| EN-1480041 | + | no | 0.0E+00 |
| EN-1481507 | + | no | 4.7E-08 |
| EN-1481538 | - | no | 3.1E-07 |
| EN-1481762 | - | yes | 3.4E-06 |
| EN-1482882 | - | yes | 0.0E+00 |
| EN-1483086 | + | no | 1.6E-08 |
| EN-1484142 | + | yes | 0.0E+00 |
| EN-1484465-68 | - | yes | 0.0E+00 |
| EN-1485282-90 | - | no | 0.0E+00 |
| EN-1485295 | - | no | 1.2E-07 |
| EN-1486456 | - | no | 2.0E-07 |
| EN-1486713 | - | no | 1.5E-08 |
| EN-1486740 | - | no | 3.4E-06 |
| EN-1486763 | + | yes | 5.0E-07 |
| EN-1487759 | - | no | 7.8E-07 |
| EN-1487926-29 | - | yes | 2.0E-07 |
| EN-1488431 | + | no | 4.7E-08 |
| EN-1489728 | - | yes | 1.2E-07 |
| EN-1489808 | + | yes | 9.4E-08 |
| EN-1491449 | + | no | 0.0E+00 |
| EN-1492227-29 | - | yes | 6.9E-07 |
| EN-1492296 | - | no | 0.0E+00 |
| EN-1492316 | + | no | 2.4E-07 |
| EN-1492451 | + | no | 0.0E+00 |
| EN-1493295 | + | no | 0.0E+00 |
| EN-1493756 | - | no | 1.4E-06 |
| EN-1494417 | - | no | 4.6E-08 |
| EN-1494571 | - | no | 3.1E-08 |
| EN-1496368 | - | no | 3.1E-08 |
| EN-1497174 | - | yes | 6.2E-08 |
| EN-1498738 | - | no | 1.5E-08 |
| EN-1498799 | + | no | 4.7E-08 |
| EN-1499629 | - | no | 1.8E-07 |
| EN-1499977 | - | no | 1.8E-06 |
| EN-1501338 | - | yes | 1.7E-07 |
| EN-1501458 | - | yes | 6.2E-08 |
| EN-1502571 | - | no | 0.0E+00 |
| EN-1502588 | + | yes | 1.6E-08 |
| EN-1503356 | - | no | 6.6E-07 |
| EN-1503390 | - | no | 1.2E-07 |
| LE-1504318 | + | only | n/a |
| EN-1504454-56 | + | no | 7.9E-08 |
| EN-1504851 | - | no | 3.4E-06 |
| EN-1505092 | - | no | 1.5E-08 |
| EN-1507309 | - | no | 1.5E-08 |
| EN-1507765 | - | yes | 1.1E-07 |
| EN-1508339 | - | no | 7.7E-08 |
| EN-1509875 | + | no | 0.0E+00 |
| EN-1511081-83 | - | yes | 1.7E-07 |
| EN-1511148 | - | yes | 1.5E-07 |
| EN-1511183 | + | no | 1.1E-07 |
| EN-1513214 | + | no | 1.1E-07 |
| EN-1514469-72 | - | no | 9.2E-08 |
| EN-1515018-20 | - | no | 7.7E-08 |
| EN-1515042 | + | no | 3.1E-08 |
| EN-1515130 | - | no | 7.7E-08 |
| EN-1515235 | - | no | 6.2E-08 |
| EN-1515426 | + | no | 4.7E-08 |
| EN-1517072 | - | no | 2.3E-07 |
| EN-1517285 | - | no | 0.0E+00 |
| EN-1517295-97 | - | yes | 1.5E-08 |
| EN-1519736 | - | no | 1.8E-07 |
| EN-1519974 | + | no | 9.4E-08 |
| LE-1520010 | + | only | n/a |
| EN-1520051-53 | + | yes | 1.6E-08 |
| EN-1520447 | - | yes | 2.2E-07 |
| LE-1520448 | - | only | n/a |
| EN-1521325 | - | no | 8.2E-06 |
| EN-1523657 | - | no | 2.5E-06 |
| EN-1525741-44 | - | no | 3.0E-06 |
| EN-1525841 | - | yes | 0.0E+00 |
| EN-1526102 | + | no | 0.0E+00 |
| EN-1526576 | + | no | 0.0E+00 |
| EN-1527036 | - | no | 3.2E-05 |
| EN-1527282 | - | no | 1.1E-07 |
| EN-1527923 | - | no | 4.6E-08 |
| EN-1528039 | - | yes | 2.0E-07 |
| EN-1528664 | - | no | 6.2E-08 |
| EN-1529311 | - | no | 4.6E-08 |
| EN-1530844 | + | no | 2.8E-07 |
| EN-1531145 | - | no | 2.5E-07 |
| EN-1531708-10 | + | yes | 0.0E+00 |
| EN-1531753 | + | no | 1.1E-07 |
| EN-1531884 | - | no | 1.5E-07 |
| EN-1532838 | + | no | 8.2E-07 |
| EN-1533693 | - | no | 2.4E-06 |
| EN-1533944 | - | no | 1.5E-08 |
| EN-1534270 | - | no | 1.1E-07 |
| EN-1534392 | - | no | 2.0E-07 |
| EN-1534405 | - | yes | 8.0E-07 |
| EN-1535756 | + | no | 1.0E-06 |
| EN-1536264-66 | - | no | 1.1E-06 |
| EN-1536907 | + | yes | 6.3E-07 |
| EN-1537644 | - | yes | 0.0E+00 |
| EN-1537655 | - | no | 1.1E-07 |
| EN-1537763 | - | no | 0.0E+00 |
| EN-1538747 | - | no | 1.8E-06 |
| EN-1539476 | - | no | 3.1E-08 |
| EN-1539794 | - | no | 6.2E-08 |
| EN-1540920 | - | no | 6.2E-08 |
| EN-1540932 | - | yes | 2.9E-07 |
| EN-1541205 | + | no | 3.0E-06 |
| EN-1543350 | - | no | 1.1E-06 |
| LE-1543535 | + | only | n/a |
| EN-1543776 | - | yes | 8.8E-07 |
| EN-1544191-93 | + | no | 3.1E-08 |
| EN-1544786 | + | no | 2.8E-07 |
| EN-1544795 | - | no | 1.1E-05 |
| EN-1544892-97 | + | yes | 5.5E-07 |
| EN-1545433 | - | no | 0.0E+00 |
| EN-1545532 | - | no | 7.7E-08 |
| EN-1545959 | - | no | 9.2E-08 |
| EN-1546367 | - | no | 6.2E-08 |
| EN-1546900 | + | yes | 0.0E+00 |
| EN-1546976 | + | no | 1.1E-07 |
| EN-1548435 | - | no | 1.5E-08 |
| EN-1551337-40 | - | no | 3.1E-07 |
| EN-1552178 | + | no | 0.0E+00 |
| EN-1552284 | - | no | 6.2E-08 |
| EN-1552855 | - | yes | 7.8E-07 |
| EN-1553441 | - | yes | 6.6E-07 |
| EN-1555189 | + | no | 0.0E+00 |
| EN-1557096 | - | no | 1.4E-06 |
| EN-1557896 | - | no | 1.4E-06 |
| LE-1558746 | - | only | n/a |
| EN-1559476 | - | yes | 5.9E-06 |
| EN-1560688 | - | no | 2.0E-07 |
| EN-1562378-80 | - | no | 7.8E-06 |
| EN-1562747 | - | no | 1.8E-07 |
| EN-1563651 | - | no | 6.2E-08 |
| EN-1563837 | - | no | 4.2E-07 |
| EN-1563909 | - | no | 0.0E+00 |
| EN-1564269 | + | no | 4.7E-08 |
| EN-1564771 | - | no | 6.0E-07 |
| EN-1564809 | - | yes | 3.3E-06 |
| EN-1565054-56 | - | no | 0.0E+00 |
| EN-1565107 | - | no | 2.3E-07 |
| EN-1565719 | + | yes | 5.7E-07 |
| EN-1565902 | - | no | 7.8E-07 |
| EN-1565921 | + | no | 2.0E-07 |
| EN-1566109-12 | + | yes | 3.1E-06 |
| EN-1567087-90 | + | no | 0.0E+00 |
| EN-1567551 | - | no | 1.5E-08 |
| EN-1568066 | + | yes | 1.6E-08 |
| EN-1568317 | + | no | 1.6E-08 |
| EN-1569464 | - | yes | 1.9E-06 |
| EN-1569592 | - | no | 6.2E-08 |
| EN-1569596-00 | + | yes | 1.6E-08 |
| EN-1570287 | + | no | 0.0E+00 |
| EN-1570435 | - | no | 2.1E-06 |
| EN-1570676 | - | no | 1.1E-07 |
| EN-1571308 | - | no | 1.5E-07 |
| EN-1572285-91 | - | yes | 2.5E-07 |
| EN-1573376-78 | + | yes | 1.1E-07 |
| EN-1573712 | + | no | 9.4E-08 |
| EN-1574039 | - | no | 1.8E-07 |
| EN-1574253-57 | + | no | 4.7E-08 |
| EN-1574275 | + | no | 1.1E-07 |
| EN-1574669 | - | no | 1.5E-08 |
| EN-1574682 | - | no | 2.6E-07 |
| EN-1574844 | - | no | 0.0E+00 |
| EN-1574863 | + | no | 1.1E-06 |
| EN-1575168 | - | no | 4.6E-08 |
| EN-1578127 | - | no | 1.5E-08 |
| EN-1579211 | + | no | 4.7E-08 |
| EN-1579211 | - | no | 0.0E+00 |
| EN-1580820 | + | no | 1.6E-08 |
| EN-1580867 | - | no | 2.0E-07 |
| LE-1580884 | + | only | n/a |
| EN-1581070 | + | no | 6.0E-07 |
| EN-1582695 | + | no | 7.9E-08 |
| EN-1582777 | - | yes | 0.0E+00 |
| EN-1583972 | - | yes | 2.2E-07 |
| EN-1584054 | - | no | 0.0E+00 |
| EN-1584150 | + | no | 0.0E+00 |
| EN-1585728-30 | - | yes | 6.2E-08 |
| EN-1585748 | - | no | 0.0E+00 |
| EN-1585989 | - | no | 1.1E-07 |
| EN-1586545 | - | no | 0.0E+00 |
| EN-1586847 | - | no | 1.8E-07 |
| EN-1586933 | + | no | 3.1E-08 |
| EN-1587956 | - | no | 4.6E-08 |
| EN-1588139 | + | no | 6.3E-08 |
| EN-1588337 | - | no | 1.5E-08 |
| EN-1589389-91 | + | no | 1.6E-08 |
| EN-1589693-95 | - | no | 1.8E-07 |
| EN-1589788 | - | no | 9.2E-08 |
| EN-1590751 | + | no | 2.8E-07 |
| EN-1592119 | + | no | 0.0E+00 |
| EN-1592124-26 | - | yes | 6.2E-08 |
| EN-1592213 | - | no | 1.0E-06 |
| EN-1592301 | - | no | 8.3E-06 |
| LE-1592629 | - | only | n/a |
| EN-1593218 | - | no | 6.2E-08 |
| EN-1593437 | + | yes | 0.0E+00 |
| EN-1593674 | - | yes | 6.3E-07 |
| EN-1596711-15 | + | no | 1.2E-06 |
| EN-1597176 | - | no | 7.8E-07 |
| EN-1597335 | - | no | 1.1E-07 |
| EN-1598268 | + | no | 0.0E+00 |
| EN-1598275 | - | no | 1.8E-06 |
| EN-1598851 | - | yes | 2.3E-07 |
| EN-1599038 | - | no | 3.1E-08 |
| EN-1600267 | - | no | 0.0E+00 |
| EN-1600273 | + | no | 0.0E+00 |
| EN-1600865 | - | no | 0.0E+00 |
| EN-1601368 | - | no | 2.7E-06 |
| EN-1601724 | - | no | 9.2E-08 |
| EN-1602609 | - | no | 3.1E-08 |
| EN-1606490-92 | - | yes | 9.4E-07 |
| EN-1608404 | - | no | 9.2E-08 |
| EN-1608469 | + | no | 1.6E-08 |
| EN-1609203 | - | no | 2.9E-07 |
| EN-1609519 | - | no | 0.0E+00 |
| EN-1609538 | + | no | 1.1E-07 |
| EN-1609679 | - | yes | 2.8E-07 |
| EN-1611849 | + | no | 5.8E-07 |
| EN-1612862 | - | yes | 1.6E-06 |
| EN-1613147-49 | - | no | 1.1E-07 |
| EN-1613779 | + | no | 4.7E-08 |
| EN-1613793 | + | no | 2.4E-07 |
| EN-1614772 | - | yes | 2.8E-06 |
| EN-1614774 | + | no | 1.6E-08 |
| EN-1614866 | + | yes | 3.1E-08 |
| EN-1614877 | - | no | 2.3E-07 |
| EN-1616105 | - | no | 1.7E-07 |
| EN-1616480 | + | no | 4.7E-08 |
| EN-1617228 | - | no | 0.0E+00 |
| EN-1617514 | + | yes | 0.0E+00 |
| EN-1618508 | - | no | 3.5E-05 |
| EN-1618560 | - | no | 0.0E+00 |
| EN-1619683 | - | no | 0.0E+00 |
| EN-1619897 | - | no | 3.5E-07 |
| LE-1620444 | - | only | n/a |
| EN-1621047 | - | yes | 1.1E-07 |
| EN-1622045 | - | no | 4.6E-08 |
| EN-1623841 | - | no | 1.4E-07 |
| EN-1624385 | - | no | 0.0E+00 |
| EN-1624508 | - | yes | 0.0E+00 |
| EN-1625655-57 | - | yes | 0.0E+00 |
| EN-1625671 | - | no | 0.0E+00 |
| EN-1629465 | - | no | 2.0E-07 |
| EN-1629806 | - | no | 1.6E-06 |
| EN-1629914 | + | no | 0.0E+00 |
| EN-1630173-76 | + | yes | 1.6E-08 |
| EN-1630340 | - | no | 1.3E-06 |
| EN-1632139-43 | - | no | 1.5E-07 |
| EN-1633757 | - | yes | 1.1E-05 |
| EN-1633844 | + | no | 2.9E-06 |
| EN-1635166 | + | no | 5.5E-08 |
| EN-1636181 | - | no | 1.1E-07 |
| EN-1636419 | - | no | 1.5E-07 |
| EN-1638926 | - | no | 2.3E-07 |
| EN-1639763 | + | no | 0.0E+00 |
| EN-1639835 | - | yes | 2.2E-07 |
| EN-1640052 | - | no | 1.5E-07 |
| EN-1641417-20 | + | no | 0.0E+00 |
| EN-1641508 | - | no | 2.7E-06 |
| EN-1642082-84 | + | yes | 0.0E+00 |
| EN-1642108-10 | - | yes | 0.0E+00 |
| EN-1642147 | + | yes | 0.0E+00 |
| EN-1642667 | - | no | 2.3E-07 |
| EN-1643075 | + | no | 0.0E+00 |
| EN-1643797-99 | - | no | 0.0E+00 |
| EN-1644133 | - | yes | 0.0E+00 |
| EN-1644241 | + | no | 8.8E-06 |
| EN-1644587 | - | no | 2.0E-07 |
| EN-1646015 | + | no | 6.0E-07 |
| EN-1648615 | + | no | 1.1E-07 |
| EN-1648953 | - | no | 0.0E+00 |
| EN-1649722 | - | no | 1.2E-07 |
| EN-1650326 | - | no | 3.1E-08 |
| EN-1650801 | + | no | 5.2E-07 |
| EN-1651011 | - | no | 6.2E-08 |
| EN-1651108 | - | no | 4.6E-08 |
| EN-1652327 | - | yes | 6.8E-07 |
| EN-1652440 | + | no | 4.7E-07 |
| EN-1653211 | - | no | 1.5E-08 |
| EN-1653574 | - | no | 1.1E-07 |
| EN-1653837 | - | no | 4.6E-08 |
| EN-1654417 | + | no | 4.7E-08 |
| EN-1654714 | - | yes | 6.9E-07 |
| EN-1654717-26 | + | yes | 1.7E-07 |
| EN-1654792 | + | no | 0.0E+00 |
| EN-1654923 | - | no | 0.0E+00 |
| EN-1654943-45 | + | no | 0.0E+00 |
| EN-1655215 | + | no | 2.0E-07 |
| EN-1655485 | - | no | 0.0E+00 |
| EN-1655593 | - | no | 6.2E-08 |
| EN-1656461 | + | no | 0.0E+00 |
| EN-1656712 | + | no | 3.3E-07 |
| EN-1657417 | - | no | 0.0E+00 |
| EN-1657474 | - | yes | 8.8E-07 |
| EN-1657675 | + | no | 9.4E-08 |
| EN-1658998 | + | no | 0.0E+00 |
| EN-1659064 | - | no | 4.6E-08 |
| EN-1659115 | - | no | 3.8E-07 |
| EN-1659517 | - | no | 1.0E-06 |
| EN-1659674 | - | no | 1.5E-07 |
| EN-1659698 | - | yes | 0.0E+00 |
| EN-1659701 | + | yes | 1.1E-07 |
| EN-1660090 | - | no | 2.6E-07 |
| EN-1660283 | - | yes | 3.1E-08 |
| EN-1660302-05 | - | no | 6.9E-07 |
| EN-1661191 | - | no | 1.1E-07 |
| EN-1661944 | - | no | 1.8E-07 |
| EN-1662214 | - | no | 4.6E-08 |
| EN-1662507 | - | no | 1.1E-07 |
| EN-1662762 | - | no | 1.1E-07 |
| EN-1662819 | - | no | 5.8E-06 |
| EN-1666385 | - | no | 1.2E-07 |
| EN-1666433 | + | no | 9.4E-08 |
| EN-1666457 | - | no | 1.5E-08 |
| EN-1667111 | + | no | 0.0E+00 |
| EN-1667190 | - | no | 4.9E-07 |
| EN-1667215 | + | no | 0.0E+00 |
| EN-1668032 | + | no | 0.0E+00 |
| EN-1669229 | - | yes | 9.5E-07 |
| EN-1669453 | + | yes | 0.0E+00 |
| EN-1671154 | - | no | 1.5E-08 |
| EN-1673205 | - | no | 9.2E-08 |
| EN-1674593 | + | yes | 1.6E-08 |
| EN-1674700-02 | - | no | 4.6E-08 |
| EN-1674962 | - | yes | 0.0E+00 |
| EN-1675033-38 | + | no | 1.6E-08 |
| EN-1675961 | - | yes | 6.2E-08 |
| EN-1676224 | - | no | 0.0E+00 |
| EN-1676911-13 | + | no | 0.0E+00 |
| EN-1677081 | + | no | 0.0E+00 |
| EN-1677185-87 | + | yes | 0.0E+00 |
| EN-1677708 | - | yes | 7.7E-08 |
| EN-1677738-42 | + | no | 2.4E-07 |
| EN-1679711 | + | no | 0.0E+00 |
| EN-1680007 | + | no | 0.0E+00 |
| EN-1680212 | - | no | 0.0E+00 |
| EN-1681003 | + | yes | 2.8E-07 |
| EN-1681920 | + | no | 0.0E+00 |
| EN-1682127 | + | no | 1.1E-07 |
| EN-1682213 | - | no | 4.6E-08 |
| EN-1682376 | - | yes | 6.9E-07 |
| EN-1682497 | + | yes | 0.0E+00 |
| EN-1685364 | - | no | 2.0E-07 |
| EN-1685607 | + | no | 2.4E-07 |
| EN-1685897 | + | no | 0.0E+00 |
| EN-1686019 | - | no | 1.5E-07 |
| EN-1686138 | + | no | 3.6E-07 |
| EN-1686266 | + | no | 3.9E-07 |
| EN-1687656 | + | yes | 9.8E-07 |
| EN-1687754-56 | + | no | 1.0E-06 |
| EN-1688769 | + | no | 3.3E-07 |
| EN-1690612 | - | no | 1.5E-08 |
| EN-1691385 | - | no | 9.2E-08 |
| EN-1691742 | + | no | 1.9E-07 |
| EN-1693656 | + | yes | 2.2E-06 |
| EN-1695940 | - | no | 0.0E+00 |
| EN-1696206 | + | no | 3.1E-08 |
| EN-1697850 | - | no | 1.5E-07 |
| EN-1697875 | + | no | 0.0E+00 |
| EN-1698788-90 | + | no | 1.6E-08 |
| EN-1699347 | - | no | 1.4E-06 |
| EN-1699359 | - | no | 0.0E+00 |
| EN-1700524 | - | no | 4.6E-08 |
| EN-1700799 | - | no | 3.1E-08 |
| EN-1701903 | - | no | 1.8E-07 |
| EN-1703887 | - | no | 0.0E+00 |
| EN-1703927 | - | no | 9.5E-07 |
| EN-1704181 | + | no | 7.9E-08 |
| EN-1704194 | + | no | 0.0E+00 |
| EN-1704218 | - | no | 2.5E-07 |
| EN-1704235 | + | no | 7.9E-08 |
| EN-1704369 | + | no | 4.7E-08 |
| EN-1704637 | - | no | 0.0E+00 |
| EN-1704830 | - | yes | 0.0E+00 |
| EN-1704848-51 | + | yes | 0.0E+00 |
| EN-1708330 | + | no | 9.4E-08 |
| EN-1708666 | - | no | 2.0E-07 |
| EN-1709410 | - | yes | 2.0E-07 |
| EN-1709508 | + | no | 1.1E-07 |
| EN-1709537 | + | no | 0.0E+00 |
| EN-1710386 | + | no | 1.1E-07 |
| EN-1719380-82 | - | yes | 4.6E-08 |
| EN-1721606-08 | + | yes | 3.1E-08 |
| EN-1722061 | + | no | 0.0E+00 |
| EN-1723065 | + | no | 4.5E-07 |
| EN-1725100 | + | no | 0.0E+00 |
| EN-1725133 | + | no | 0.0E+00 |
| EN-1725282 | + | no | 1.4E-07 |
| EN-1725349 | + | yes | 0.0E+00 |
| EN-1726272 | - | no | 1.1E-07 |
| EN-1727918 | + | yes | 0.0E+00 |
| EN-1731448 | + | yes | 2.8E-06 |
| EN-1731631-34 | + | no | 0.0E+00 |
| EN-1731985-87 | + | yes | 0.0E+00 |
| EN-1733299 | - | no | 2.3E-07 |
| EN-1734800 | - | no | 6.2E-08 |
| EN-1735239 | - | no | 1.5E-08 |
| EN-1736349 | - | yes | 0.0E+00 |
| EN-1737885 | - | no | 4.6E-08 |
| EN-1738456 | + | no | 4.7E-08 |
| EN-1738513 | - | no | 0.0E+00 |
| EN-1739650 | - | yes | 6.2E-08 |
| EN-1739953 | + | no | 2.7E-07 |
| EN-1741194 | - | no | 1.1E-07 |
| EN-1741332-34 | - | yes | 3.1E-08 |
| EN-1743424 | - | no | 1.5E-07 |
| EN-1744022 | - | no | 0.0E+00 |
| EN-1744298 | - | no | 0.0E+00 |
| EN-1744718 | - | no | 9.2E-08 |
| EN-1745088 | + | no | 0.0E+00 |
| EN-1745088 | - | no | 0.0E+00 |
| EN-1746207-09 | - | yes | 0.0E+00 |
| EN-1746458 | + | yes | 1.6E-08 |
| EN-1747014 | - | yes | 0.0E+00 |
| EN-1747200 | - | yes | 3.1E-08 |
| EN-1748228 | + | no | 1.3E-07 |
| EN-1748728 | - | no | 0.0E+00 |
| EN-1748846 | - | no | 1.5E-08 |
| EN-1749662 | - | yes | 0.0E+00 |
| EN-1750161 | + | no | 0.0E+00 |
| EN-1750334 | - | no | 4.6E-08 |
| EN-1752170 | - | no | 1.4E-06 |
| EN-1752540-43 | - | no | 0.0E+00 |
| EN-1753994 | - | no | 0.0E+00 |
| EN-1754750 | - | yes | 2.1E-06 |
| EN-1755028 | + | no | 1.6E-08 |
| EN-1755037 | - | yes | 4.6E-08 |
| EN-1755654 | - | no | 0.0E+00 |
| EN-1756048 | - | no | 3.1E-08 |
| EN-1756113 | - | no | 2.3E-07 |
| EN-1757297 | - | no | 6.2E-08 |
| EN-1758041 | - | no | 0.0E+00 |
| EN-1758976 | - | no | 0.0E+00 |
| EN-1759085 | - | no | 6.2E-08 |
| EN-1759448 | - | no | 1.8E-06 |
| EN-1760382 | - | no | 0.0E+00 |
| EN-1761197 | - | yes | 1.5E-08 |
| EN-1762569 | - | yes | 1.4E-07 |
| EN-1762886 | + | no | 3.3E-07 |
| EN-1763960 | - | no | 2.0E-07 |
| EN-1764318 | - | no | 9.2E-08 |
| EN-1765724 | - | no | 6.2E-08 |
| EN-1765894 | - | no | 0.0E+00 |
| EN-1766057 | - | no | 1.8E-07 |
| LE-1766231 | - | only | n/a |
| EN-1766484 | - | no | 1.5E-08 |
| EN-1766501 | - | yes | 0.0E+00 |
| EN-1766709 | + | no | 3.6E-07 |
| EN-1768327 | - | no | 0.0E+00 |
| EN-1768382-85 | - | yes | 1.2E-07 |
| LE-1769215 | + | only | n/a |
| EN-1769416 | + | no | 0.0E+00 |
| LE-1769634 | - | only | n/a |
| EN-1770284 | - | no | 1.5E-07 |
| EN-1772806-08 | - | no | 0.0E+00 |
| EN-1773711 | + | no | 9.4E-08 |
| EN-1774754 | - | no | 0.0E+00 |
| EN-1774887 | + | no | 0.0E+00 |
| EN-1775951 | - | yes | 9.2E-07 |
| EN-1775961 | + | yes | 9.4E-08 |
| EN-1779371 | - | no | 4.6E-08 |
| EN-1779417-19 | - | no | 1.1E-07 |
| EN-1779798 | + | no | 4.7E-08 |
| EN-1781907 | - | no | 3.1E-08 |
| EN-1782042 | - | no | 1.5E-08 |
| EN-1782254 | - | no | 1.1E-07 |
| EN-1783516 | - | no | 6.2E-08 |
| EN-1786002 | - | no | 0.0E+00 |
| EN-1786033 | + | yes | 0.0E+00 |
| EN-1786598 | - | no | 9.2E-08 |
| EN-1786824 | + | no | 4.7E-08 |
| EN-1786919 | + | no | 3.1E-08 |
| EN-1787130 | - | no | 1.1E-07 |
| EN-1787483 | - | yes | 6.9E-07 |
| EN-1787638 | - | no | 5.4E-07 |
| EN-1787716 | - | yes | 6.9E-07 |
| EN-1788840 | - | no | 2.2E-07 |
| EN-1789002-04 | + | yes | 1.6E-08 |
| EN-1789368 | + | no | 1.1E-07 |
| EN-1789681 | + | yes | 4.7E-08 |
| EN-1791497 | + | no | 7.9E-08 |
| EN-1791515-17 | - | yes | 3.1E-08 |
| EN-1792446 | + | no | 0.0E+00 |
| EN-1792950 | - | no | 0.0E+00 |
| EN-1793012 | + | no | 0.0E+00 |
| EN-1793962 | - | no | 1.5E-08 |
| EN-1794919 | - | no | 6.2E-08 |
| EN-1795031-39 | - | no | 1.2E-07 |
| EN-1795423 | - | no | 6.2E-08 |
| EN-1795830 | - | no | 7.7E-08 |
| EN-1796280 | + | yes | 0.0E+00 |
| EN-1796353 | + | no | 5.0E-07 |
| EN-1796417-19 | + | no | 7.9E-08 |
| EN-1796487 | + | no | 1.1E-07 |
| EN-1796813 | - | yes | 2.0E-07 |
| LE-1796814 | - | only | n/a |
| EN-1797691 | - | no | 2.1E-05 |
| EN-1798245 | - | no | 1.3E-05 |
| EN-1800026 | - | no | 1.5E-07 |
| EN-1800511 | - | no | 3.0E-06 |
| EN-1802110-13 | - | no | 2.0E-07 |
| EN-1802210-12 | - | no | 2.5E-07 |
| EN-1802377 | - | yes | 0.0E+00 |
| EN-1803522-24 | + | yes | 0.0E+00 |
| EN-1803551 | + | no | 1.1E-07 |
| EN-1805363 | + | no | 4.6E-07 |
| EN-1807051-53 | - | no | 6.2E-08 |
| EN-1807171 | - | no | 0.0E+00 |
| EN-1807767 | - | no | 1.7E-07 |
| EN-1807866 | - | no | 9.2E-08 |
| EN-1807920 | - | yes | 6.3E-07 |
| EN-1808078 | + | yes | 0.0E+00 |
| EN-1808873 | - | no | 0.0E+00 |
| EN-1808969 | - | no | 1.8E-07 |
| EN-1810097 | - | no | 1.2E-07 |
| EN-1810139-43 | + | yes | 4.7E-06 |
| EN-1810520-22 | + | no | 2.4E-07 |
| EN-1811097 | - | no | 1.2E-07 |
| EN-1811823 | - | no | 9.2E-08 |
| EN-1812325 | + | no | 3.1E-08 |
| EN-1812510 | + | no | 1.1E-07 |
| EN-1813559 | - | no | 1.5E-08 |
| EN-1814032 | - | no | 6.3E-06 |
| EN-1814558 | - | yes | 2.0E-07 |
| EN-1814583 | + | yes | 4.7E-08 |
| EN-1814613 | - | no | 1.4E-07 |
| EN-1814931 | + | yes | 0.0E+00 |
| EN-1815018-22 | + | yes | 2.8E-07 |
| EN-1815341 | + | no | 4.7E-08 |
| EN-1815487 | - | yes | 9.2E-08 |
| EN-1815599 | + | yes | 4.7E-07 |
| EN-1815879 | + | no | 7.6E-06 |
| EN-1817604 | + | no | 1.6E-08 |
| EN-1817955 | + | no | 0.0E+00 |
| EN-1818018 | + | no | 3.1E-06 |
| EN-1818965 | + | no | 7.9E-08 |
| EN-1819301 | - | no | 0.0E+00 |
| EN-1819829 | - | no | 0.0E+00 |
| EN-1820063 | - | no | 9.2E-08 |
| EN-1820242 | + | no | 0.0E+00 |
| EN-1820454 | - | yes | 0.0E+00 |
| EN-1820530 | + | no | 0.0E+00 |
| EN-1821639 | - | no | 0.0E+00 |
| EN-1821751 | - | no | 1.1E-07 |
| EN-1822085 | - | yes | 0.0E+00 |
| EN-1822114 | - | no | 6.2E-08 |
| EN-1822694 | - | no | 1.5E-08 |
| EN-1823358 | + | no | 0.0E+00 |
| EN-1824262 | - | no | 6.9E-06 |
| EN-1825973 | - | no | 1.5E-07 |
| EN-1826450 | - | no | 7.8E-07 |
| EN-1826568 | - | no | 0.0E+00 |
| EN-1826684 | + | no | 0.0E+00 |
| EN-1827144-50 | - | no | 2.0E-07 |
| EN-1828244 | - | yes | 4.6E-08 |
| LE-1829163 | - | only | n/a |
| EN-1829669 | - | no | 2.6E-07 |
| EN-1830418 | + | no | 7.9E-08 |
| EN-1831636 | + | yes | 4.7E-08 |
| EN-1832375 | - | no | 3.1E-08 |
| EN-1832945 | - | no | 0.0E+00 |
| EN-1833078 | - | yes | 6.0E-07 |
| EN-1833164 | + | no | 3.3E-07 |
| EN-1833408 | - | no | 0.0E+00 |
| EN-1834109 | - | no | 9.2E-08 |
| EN-1834546 | - | yes | 2.2E-07 |
| LE-1834644 | + | only | n/a |
| EN-1836320 | - | no | 1.1E-07 |
| EN-1837265 | - | no | 1.2E-05 |
| EN-1838099 | - | no | 3.2E-06 |
| LE-1838459 | + | only | n/a |
| EN-1838541 | - | no | 1.1E-07 |
| EN-1839372 | - | no | 1.5E-07 |
| EN-1839715 | - | no | 1.5E-07 |
| EN-1841322 | - | no | 0.0E+00 |
| EN-1841477 | - | no | 0.0E+00 |
| EN-1841861 | - | yes | 8.5E-06 |
| EN-1841993 | + | no | 7.1E-07 |
| EN-1842783 | + | no | 1.1E-07 |
| EN-1843585 | - | no | 0.0E+00 |
| EN-1845286 | + | no | 0.0E+00 |
| EN-1846391 | - | no | 0.0E+00 |
| EN-1846670-72 | + | no | 1.6E-08 |
| EN-1847347-49 | - | no | 3.5E-07 |
| EN-1847651 | + | yes | 0.0E+00 |
| EN-1847694 | - | no | 2.5E-07 |
| EN-1847903 | - | no | 4.4E-06 |
| EN-1848436 | + | no | 0.0E+00 |
| EN-1849359 | - | no | 0.0E+00 |
| EN-1849598 | - | yes | 2.3E-07 |
| EN-1849674 | + | no | 4.7E-08 |
| EN-1850624 | - | no | 2.0E-07 |
| EN-1851422 | - | yes | 1.3E-06 |
| EN-1851441 | + | no | 0.0E+00 |
| EN-1851527 | + | yes | 1.0E-05 |
| EN-1851959 | + | no | 0.0E+00 |
| EN-1852031 | - | no | 9.2E-08 |
| EN-1852561-66 | + | no | 0.0E+00 |
| EN-1852784 | - | no | 6.2E-08 |
| EN-1852816 | + | no | 0.0E+00 |
| EN-1853030 | + | no | 0.0E+00 |
| EN-1853403 | + | yes | 9.3E-06 |
| EN-1853510-12 | + | yes | 2.1E-05 |
| EN-1853945 | + | yes | 0.0E+00 |
| EN-1855932 | - | no | 2.0E-07 |
| EN-1857134 | + | yes | 1.6E-08 |
| EN-1857795 | - | yes | 3.9E-06 |
| LE-1857796 | - | only | n/a |
| EN-1857910 | + | no | 2.8E-07 |
| EN-1859144 | - | no | 4.6E-08 |
| EN-1859254 | - | no | 1.8E-07 |
| EN-1859353 | - | no | 4.0E-06 |
| EN-1860031 | - | yes | 9.2E-08 |
| EN-1860041 | - | no | 0.0E+00 |
| EN-1860100 | - | no | 5.1E-07 |
| EN-1860106 | + | no | 7.9E-07 |
| EN-1860615 | - | no | 6.2E-08 |
| EN-1860626 | + | no | 5.5E-07 |
| EN-1860940 | + | no | 1.6E-08 |
| EN-1862039 | - | no | 9.2E-08 |
| EN-1864065 | - | yes | 1.1E-05 |
| EN-1864261 | + | no | 1.1E-07 |
| EN-1865649 | - | no | 6.2E-08 |
| LE-1865903 | - | only | n/a |
| EN-1866517 | - | no | 1.5E-07 |
| EN-1866841 | + | no | 0.0E+00 |
| LE-1867937-41 | + | only | n/a |
| EN-1869739 | - | no | 2.2E-07 |
| EN-1869942 | - | no | 1.1E-07 |
| EN-1870201 | - | no | 6.9E-07 |
| EN-1870419 | - | no | 1.3E-06 |
| EN-1870483 | - | no | 1.0E-06 |
| EN-1870536 | - | no | 0.0E+00 |
| EN-1871171 | - | yes | 6.0E-07 |
| EN-1871388 | + | no | 2.8E-07 |
| EN-1871601 | - | yes | 0.0E+00 |
| EN-1871772 | - | no | 0.0E+00 |
| EN-1872079 | - | yes | 2.8E-07 |
| EN-1872091 | - | yes | 0.0E+00 |
| EN-1872103 | + | no | 0.0E+00 |
| EN-1873251 | + | yes | 5.0E-07 |
| EN-1873865 | - | no | 0.0E+00 |
| EN-1875395 | - | no | 1.8E-07 |
| EN-1875582 | + | yes | 9.4E-08 |
| EN-1876556 | - | no | 0.0E+00 |
| LE-1879738 | + | only | n/a |
| EN-1879761 | - | no | 0.0E+00 |
| EN-1879834 | - | yes | 1.7E-07 |
| EN-1879978 | + | no | 4.7E-08 |
| EN-1880061 | + | no | 7.9E-08 |
| EN-1881518 | - | no | 0.0E+00 |
| EN-1882549 | - | no | 2.0E-07 |
| EN-1882615 | + | no | 0.0E+00 |
| EN-1882888 | - | no | 1.5E-08 |
| EN-1883334-36 | - | no | 1.1E-07 |
| EN-1885077 | - | no | 1.9E-06 |
| EN-1885095 | + | no | 0.0E+00 |
| EN-1886200 | - | no | 3.1E-08 |
| EN-1886452 | + | no | 0.0E+00 |
| EN-1886603 | + | yes | 1.6E-08 |
| EN-1886962 | - | no | 9.2E-08 |
| EN-1888731 | - | no | 1.6E-06 |
| EN-1889555 | - | no | 0.0E+00 |
| EN-1889958 | - | no | 0.0E+00 |
| EN-1890428 | + | no | 2.4E-07 |
| EN-1890531 | + | no | 0.0E+00 |
| EN-1891208 | - | no | 6.2E-08 |
| EN-1891265 | + | no | 4.6E-07 |
| EN-1891847 | + | yes | 0.0E+00 |
| EN-1892037 | - | no | 2.8E-07 |
| EN-1892096 | - | yes | 9.7E-07 |
| EN-1892720 | + | yes | 0.0E+00 |
| EN-1893811 | + | no | 1.6E-07 |
| EN-1893824 | + | no | 0.0E+00 |
| EN-1894898 | - | yes | 3.9E-06 |
| EN-1895371 | - | no | 4.6E-08 |
| EN-1896694 | - | no | 1.5E-07 |
| EN-1896843 | - | no | 2.0E-07 |
| EN-1898255 | + | yes | 0.0E+00 |
| EN-1898279 | - | no | 9.2E-08 |
| EN-1898361 | - | no | 2.6E-06 |
| EN-1898472 | - | yes | 2.5E-07 |
| EN-1899175 | - | no | 4.2E-07 |
| EN-1899473 | - | no | 1.1E-07 |
| EN-1899485 | + | yes | 3.1E-08 |
| EN-1900308-11 | + | no | 7.0E-06 |
| EN-1902900 | + | yes | 0.0E+00 |
| EN-1903651-53 | - | yes | 2.2E-06 |
| EN-1904268 | + | yes | 0.0E+00 |
| EN-1904468 | + | no | 1.6E-08 |
| EN-1904776 | - | no | 0.0E+00 |
| EN-1905362 | - | no | 2.6E-07 |
| EN-1905811 | - | yes | 9.2E-08 |
| EN-1907128 | - | yes | 0.0E+00 |
| EN-1908014-20 | + | yes | 7.9E-08 |
| EN-1909756 | + | no | 0.0E+00 |
| EN-1910146 | - | no | 0.0E+00 |
| EN-1910183-85 | + | yes | 7.9E-08 |
| EN-1910819-21 | + | yes | 1.6E-06 |
| EN-1911287-89 | + | yes | 1.6E-05 |
| EN-1913792 | - | yes | 0.0E+00 |
| EN-1913925 | + | no | 1.6E-08 |
| EN-1914388 | - | no | 0.0E+00 |
| EN-1915392 | - | yes | 0.0E+00 |
| EN-1916915 | - | no | 3.1E-08 |
| EN-1916948 | - | no | 1.5E-07 |
| LE-1917707 | - | only | n/a |
| EN-1917782 | - | no | 1.9E-06 |
| EN-1918753 | + | no | 9.8E-07 |
| EN-1920243 | - | no | 0.0E+00 |
| EN-1920360 | + | yes | 3.6E-07 |
| EN-1920608 | - | yes | 5.4E-06 |
| EN-1920956 | - | no | 0.0E+00 |
| EN-1921100 | - | no | 0.0E+00 |
| EN-1921394 | - | no | 1.5E-08 |
| EN-1922013 | - | no | 1.2E-07 |
| EN-1923444 | - | no | 0.0E+00 |
| EN-1923529 | + | no | 1.6E-08 |
| EN-1923762 | - | no | 2.2E-07 |
| EN-1924864 | + | no | 7.9E-06 |
| LE-1924939 | + | only | n/a |
| EN-1925370 | + | no | 7.2E-06 |
| EN-1925636 | + | no | 0.0E+00 |
| EN-1927522 | - | yes | 1.5E-06 |
| EN-1927678 | + | no | 3.3E-07 |
| EN-1930288 | - | no | 2.5E-07 |
| EN-1930324 | - | no | 1.5E-08 |
| EN-1931277 | + | no | 0.0E+00 |
| EN-1931376 | - | yes | 0.0E+00 |
| EN-1932732 | - | no | 0.0E+00 |
| EN-1932984 | - | yes | 1.4E-06 |
| LE-1932984-87 | - | only | n/a |
| LE-1933084 | + | only | n/a |
| EN-1933799 | + | no | 0.0E+00 |
| EN-1934903 | + | yes | 0.0E+00 |
| EN-1935534 | + | no | 2.0E-07 |
| EN-1935889 | - | yes | 1.6E-05 |
| EN-1937510 | - | no | 2.0E-07 |
| LE-1938065-67 | - | only | n/a |
| EN-1939142 | - | no | 2.0E-07 |
| EN-1940062 | - | no | 4.6E-06 |
| EN-1941266 | - | yes | 1.5E-06 |
| EN-1941314-16 | + | yes | 0.0E+00 |
| EN-1942118 | - | no | 4.2E-07 |
| EN-1943110 | - | yes | 3.1E-08 |
| EN-1948074 | - | no | 7.7E-08 |
| EN-1948769-71 | - | yes | 7.4E-07 |
| EN-1948886 | - | no | 0.0E+00 |
| EN-1948896 | + | yes | 1.1E-07 |
| EN-1949326 | + | yes | 0.0E+00 |
| EN-1952065 | - | yes | 1.7E-07 |
| EN-1952238 | - | no | 2.0E-07 |
| EN-1952285 | - | no | 7.8E-07 |
| EN-1953294 | - | no | 0.0E+00 |
| EN-1954713 | - | no | 2.3E-07 |
| EN-1955301 | - | no | 0.0E+00 |
| EN-1955350 | - | no | 1.1E-06 |
| EN-1955392 | + | no | 3.1E-08 |
| EN-1955452 | - | no | 2.9E-06 |
| EN-1955577 | + | no | 0.0E+00 |
| LE-1955599 | - | only | n/a |
| EN-1955621 | + | no | 4.7E-08 |
| EN-1955790 | + | yes | 4.4E-07 |
| EN-1956084 | - | no | 2.3E-07 |
| EN-1958462 | - | yes | 1.0E-06 |
| EN-1958857 | - | no | 1.1E-07 |
| EN-1959205-12 | - | no | 0.0E+00 |
| EN-1959537 | - | no | 4.8E-07 |
| EN-1959743 | - | no | 0.0E+00 |
| EN-1959764 | + | no | 0.0E+00 |
| EN-1960526 | + | no | 3.3E-07 |
| EN-1960667-69 | - | no | 2.0E-07 |
| EN-1960826 | - | yes | 1.7E-06 |
| EN-1960972 | + | no | 0.0E+00 |
| EN-1961192 | - | no | 6.2E-07 |
| EN-1962696 | - | no | 0.0E+00 |
| EN-1963188 | - | no | 6.2E-08 |
| EN-1963268 | + | no | 9.4E-08 |
| EN-1963706 | - | yes | 0.0E+00 |
| EN-1963761 | - | no | 0.0E+00 |
| EN-1966432 | - | yes | 1.5E-08 |
| EN-1966776 | - | no | 0.0E+00 |
| EN-1967453 | - | no | 9.2E-08 |
| LE-1967831 | - | only | n/a |
| EN-1968581-83 | + | no | 3.1E-08 |
| EN-1969230 | - | no | 7.7E-08 |
| EN-1970777 | - | no | 1.5E-08 |
| EN-1972051-54 | + | no | 0.0E+00 |
| EN-1972647 | - | yes | 1.4E-07 |
| EN-1972782-85 | - | no | 7.8E-07 |
| EN-1972911 | + | yes | 3.1E-08 |
| EN-1974664 | - | no | 0.0E+00 |
| EN-1975202-04 | - | no | 0.0E+00 |
| EN-1975215-21 | + | yes | 1.1E-07 |
| EN-1975280 | + | no | 0.0E+00 |
| EN-1975517 | - | no | 2.0E-07 |
| EN-1975529 | - | yes | 2.5E-07 |
| EN-1977621 | - | no | 2.2E-07 |
| EN-1978836 | + | no | 6.7E-06 |
| LE-1978885 | + | only | n/a |
| EN-1979784-86 | - | yes | 1.5E-07 |
| EN-1981898 | + | no | 2.4E-07 |
| EN-1982262 | - | no | 0.0E+00 |
| EN-1982271 | - | no | 8.6E-07 |
| EN-1982462 | - | yes | 2.0E-07 |
| LE-1982468 | - | only | n/a |
| EN-1982995 | - | yes | 1.5E-08 |
| LE-1982995-98 | - | only | n/a |
| EN-1983095 | + | yes | 9.4E-08 |
| EN-1983686 | - | no | 0.0E+00 |
| EN-1983706 | + | no | 4.7E-08 |
| EN-1984856 | + | no | 0.0E+00 |
| EN-1985914 | - | no | 9.2E-08 |
| EN-1986782 | - | yes | 0.0E+00 |
| EN-1988231 | + | no | 7.9E-08 |
| EN-1988894 | - | no | 1.7E-07 |
| EN-1989311 | + | no | 0.0E+00 |
| EN-1990443 | + | no | 2.8E-07 |
| EN-1990644 | + | yes | 1.6E-08 |
| EN-1991895 | + | no | 0.0E+00 |
| EN-1992306 | - | no | 0.0E+00 |
| EN-1994024-26 | - | yes | 4.2E-07 |
| EN-1996134 | - | yes | 1.2E-06 |
| EN-1996221-24 | - | no | 7.0E-06 |
| EN-1997373 | + | no | 0.0E+00 |
| EN-2000389 | + | no | 2.2E-07 |
| EN-2000878 | + | no | 0.0E+00 |
| EN-2001096 | + | yes | 0.0E+00 |
| EN-2001178 | - | no | 9.2E-08 |
| EN-2001196-98 | + | no | 0.0E+00 |
| EN-2001463 | - | no | 0.0E+00 |
| EN-2001518 | + | yes | 1.6E-08 |
| EN-2001521 | - | yes | 1.2E-07 |
| EN-2001538-41 | + | no | 5.5E-07 |
| EN-2001712 | - | yes | 0.0E+00 |
| EN-2002195 | - | no | 0.0E+00 |
| EN-2002236 | - | no | 0.0E+00 |
| EN-2002237 | + | no | 4.7E-08 |
| EN-2002569 | - | no | 0.0E+00 |
| EN-2004267 | + | no | 0.0E+00 |
| EN-2004280 | - | no | 6.2E-08 |
| EN-2004440 | + | no | 4.7E-08 |
| EN-2004452 | - | yes | 0.0E+00 |
| EN-2004645 | + | no | 0.0E+00 |
| EN-2005989 | + | no | 0.0E+00 |
| EN-2006270 | - | no | 0.0E+00 |
| EN-2006329 | - | no | 0.0E+00 |
| EN-2007531 | + | yes | 0.0E+00 |
| EN-2009717 | + | no | 4.7E-08 |
| EN-2010444 | + | no | 2.8E-07 |
| EN-2011448 | + | no | 4.7E-08 |
| EN-2011787 | - | yes | 7.9E-06 |
| EN-2012025 | - | no | 0.0E+00 |
| EN-2012040 | - | no | 0.0E+00 |
| EN-2013058 | - | no | 0.0E+00 |
| EN-2013591 | - | no | 6.2E-08 |
| EN-2017344 | - | no | 2.0E-07 |
| EN-2018144 | - | no | 6.3E-06 |
| EN-2018252-55 | - | no | 6.2E-08 |
| EN-2019214 | - | no | 0.0E+00 |
| EN-2019673 | + | no | 1.6E-08 |
| EN-2019922 | + | no | 4.7E-08 |
| EN-2020828 | + | no | 0.0E+00 |
| EN-2020866 | + | no | 0.0E+00 |
| EN-2022623 | - | no | 7.8E-07 |
| EN-2023041 | - | no | 3.1E-08 |
| EN-2023378 | - | no | 2.3E-07 |
| EN-2023428 | + | no | 2.8E-07 |
| EN-2023539 | - | no | 1.7E-07 |
| EN-2023680 | - | no | 0.0E+00 |
| EN-2025671-73 | + | yes | 6.3E-08 |
| LE-2027269 | + | only | n/a |
| EN-2027299 | + | no | 0.0E+00 |
| EN-2027641-44 | + | no | 7.9E-08 |
| EN-2030478 | - | yes | 2.0E-07 |
| EN-2030495 | + | yes | 0.0E+00 |
| EN-2031262 | - | no | 2.0E-07 |
| EN-2032564 | - | no | 1.8E-07 |
| EN-2032666 | - | yes | 0.0E+00 |
| EN-2032886 | + | no | 3.1E-08 |
| EN-2034530 | + | no | 0.0E+00 |
| EN-2035085 | - | no | 1.7E-07 |
| EN-2035634 | + | no | 5.5E-07 |
| EN-2035805 | - | no | 2.3E-07 |
| EN-2036269 | + | yes | 0.0E+00 |
| LE-2037398-00 | - | only | n/a |
| EN-2037519 | + | no | 1.6E-08 |
| EN-2037624 | + | no | 2.0E-07 |
| EN-2038058 | - | no | 0.0E+00 |
| EN-2038078 | - | yes | 1.6E-06 |
| LE-2038081 | + | only | n/a |
| EN-2038206-09 | - | yes | 1.5E-08 |
| EN-2038493 | - | no | 2.9E-06 |
| EN-2038659 | + | no | 1.2E-06 |
| EN-2038845 | - | no | 6.2E-08 |
| EN-2038915 | - | no | 1.5E-07 |
| EN-2039370 | + | no | 1.6E-08 |
| EN-2039778 | - | no | 4.6E-08 |
| EN-2039918 | - | no | 7.8E-07 |
| EN-2041239-41 | - | no | 4.2E-07 |
| EN-2041323 | - | no | 1.1E-07 |
| EN-2041398 | - | no | 1.3E-06 |
| EN-2042310 | + | no | 0.0E+00 |
| EN-2042431 | - | no | 2.3E-06 |
| EN-2044430-32 | + | no | 1.1E-07 |
| EN-2046045-47 | + | no | 3.1E-07 |
| EN-2046435 | + | no | 9.4E-08 |
| EN-2048496 | + | no | 0.0E+00 |
| EN-2048788 | - | yes | 6.0E-07 |
| EN-2048806 | - | no | 2.0E-05 |
| EN-2048808 | + | yes | 1.7E-07 |
| EN-2048851 | - | no | 9.2E-08 |
| EN-2050197 | - | no | 2.3E-07 |
| EN-2050225 | - | no | 1.5E-08 |
| EN-2050281 | + | yes | 0.0E+00 |
| EN-2050512 | + | no | 0.0E+00 |
| EN-2052410 | - | no | 1.0E-06 |
| EN-2052430 | - | no | 1.5E-07 |
| EN-2052639 | - | no | 2.8E-07 |
| EN-2052959 | - | no | 0.0E+00 |
| EN-2053608 | - | yes | 9.2E-08 |
| EN-2053617 | + | no | 3.1E-08 |
| EN-2053827 | - | no | 3.4E-07 |
| EN-2055084 | - | no | 3.1E-08 |
| EN-2055823 | + | no | 1.7E-06 |
| EN-2056643 | + | no | 4.7E-08 |
| EN-2056689 | + | no | 9.4E-08 |
| EN-2057156 | - | yes | 6.2E-08 |
| EN-2057181 | - | yes | 1.2E-07 |
| EN-2057742 | - | yes | 1.1E-06 |
| EN-2058210 | - | yes | 1.4E-06 |
| EN-2059237 | - | no | 0.0E+00 |
| EN-2059927 | - | no | 4.0E-07 |
| EN-2060023 | - | yes | 2.6E-06 |
| EN-2061020 | - | no | 0.0E+00 |
| EN-2061490 | - | no | 9.2E-07 |
| EN-2062979-81 | - | yes | 2.5E-07 |
| EN-2062989 | - | yes | 0.0E+00 |
| EN-2063011-14 | + | yes | 1.1E-07 |
| EN-2063081 | + | yes | 3.1E-08 |
| EN-2064558 | - | yes | 0.0E+00 |
| EN-2064861 | - | no | 1.5E-08 |
| EN-2065065 | - | no | 1.5E-07 |
| EN-2065325 | - | yes | 0.0E+00 |
| EN-2065338 | - | yes | 4.6E-08 |
| EN-2065342 | + | no | 1.1E-07 |
| EN-2066299 | + | yes | 0.0E+00 |
| EN-2066686 | + | no | 2.8E-06 |
| EN-2067107-10 | - | yes | 3.1E-08 |
| EN-2067420 | + | yes | 0.0E+00 |
| EN-2068267 | + | no | 0.0E+00 |
| LE-2068763 | - | only | n/a |
| EN-2070668 | + | yes | 1.1E-07 |
| EN-2071007 | - | no | 0.0E+00 |
| EN-2072644 | - | no | 1.5E-08 |
| EN-2072755 | - | no | 2.0E-07 |
| EN-2073330-36 | - | no | 1.5E-07 |
| EN-2075184 | - | no | 0.0E+00 |
| EN-2075431 | - | yes | 1.5E-08 |
| EN-2075611 | + | yes | 0.0E+00 |
| EN-2075681 | - | no | 2.8E-06 |
| EN-2077429 | - | yes | 0.0E+00 |
| EN-2077567 | - | no | 0.0E+00 |
| EN-2077884 | - | no | 2.0E-07 |
| EN-2078393 | - | no | 0.0E+00 |
| EN-2078503-05 | - | no | 1.1E-07 |
| EN-2079187 | - | no | 0.0E+00 |
| EN-2079600 | - | no | 1.5E-07 |
| EN-2079945 | + | no | 2.8E-07 |
| LE-2080050 | - | only | n/a |
| EN-2080816 | + | no | 0.0E+00 |
| EN-2081877 | - | no | 1.5E-07 |
| EN-2082949-51 | - | no | 2.0E-07 |
| EN-2084965 | - | no | 0.0E+00 |
| EN-2086659 | + | no | 1.1E-07 |
| EN-2087004 | - | no | 1.5E-08 |
| EN-2087693 | + | no | 0.0E+00 |
| EN-2089837 | - | no | 0.0E+00 |
| EN-2091894 | - | no | 1.5E-08 |
| EN-2094109 | - | no | 9.2E-08 |
| EN-2094910 | - | no | 3.1E-08 |
| EN-2094950 | + | no | 0.0E+00 |
| EN-2096962 | + | no | 1.6E-08 |
| EN-2097748 | - | no | 2.5E-07 |
| EN-2097793-95 | - | yes | 3.5E-07 |
| EN-2097916 | - | no | 9.2E-08 |
| EN-2097925 | + | no | 3.1E-08 |
| EN-2097934 | + | no | 9.4E-08 |
| EN-2099158 | - | no | 1.5E-07 |
| EN-2099188 | - | no | 2.2E-07 |
| EN-2099322 | - | yes | 2.5E-06 |
| LE-2099325 | + | only | n/a |
| EN-2099838 | - | no | 6.0E-07 |
| EN-2099989 | + | no | 0.0E+00 |
| EN-2100757 | - | yes | 1.5E-07 |
| EN-2100859-62 | - | no | 6.2E-08 |
| EN-2101241 | + | no | 6.8E-07 |
| EN-2103674 | - | no | 0.0E+00 |
| EN-2103919 | - | no | 0.0E+00 |
| LE-2103935 | + | only | n/a |
| EN-2104020-22 | - | yes | 1.5E-08 |
| EN-2104751 | - | no | 0.0E+00 |
| EN-2105063 | - | no | 1.1E-07 |
| EN-2105835 | + | no | 0.0E+00 |
| EN-2105943 | - | yes | 0.0E+00 |
| EN-2107385 | + | no | 1.1E-07 |
| EN-2107559 | - | no | 6.2E-08 |
| EN-2107761 | - | no | 1.8E-07 |
| EN-2108923 | + | yes | 6.6E-06 |
| EN-2109011 | - | no | 0.0E+00 |
| EN-2109724 | - | yes | 0.0E+00 |
| EN-2109836-38 | - | yes | 0.0E+00 |
| EN-2109843 | + | no | 0.0E+00 |
| EN-2110693 | - | yes | 5.8E-07 |
| EN-2111582 | - | no | 3.0E-06 |
| LE-2112549 | - | only | n/a |
| LE-2113770 | + | only | n/a |
| EN-2113979 | + | no | 0.0E+00 |
| EN-2115422 | + | no | 0.0E+00 |
| EN-2116131 | - | yes | 1.1E-05 |
| EN-2116205-09 | + | no | 0.0E+00 |
| EN-2117368 | + | no | 1.6E-08 |
| EN-2118815 | - | no | 4.2E-07 |
| EN-2119436 | - | no | 2.5E-07 |
| EN-2121161 | - | no | 1.8E-07 |
| EN-2121338 | + | no | 0.0E+00 |
| EN-2121527 | + | no | 0.0E+00 |
| EN-2121717 | - | no | 0.0E+00 |
| EN-2121990 | - | no | 0.0E+00 |
| EN-2122352 | - | no | 2.0E-06 |
| EN-2122576 | - | yes | 0.0E+00 |
| EN-2123865 | - | no | 4.6E-08 |
| EN-2123901 | + | no | 0.0E+00 |
| EN-2124640 | - | yes | 1.7E-07 |
| LE-2124640-45 | - | only | n/a |
| EN-2124998 | + | yes | 6.6E-06 |
| EN-2125483 | - | no | 0.0E+00 |
| EN-2125922 | - | no | 9.2E-08 |
| EN-2125953 | - | no | 0.0E+00 |
| EN-2126022 | + | no | 1.1E-07 |
| LE-2126413 | + | only | n/a |
| EN-2126592 | - | yes | 0.0E+00 |
| EN-2126732 | - | no | 0.0E+00 |
| EN-2126817 | - | no | 6.2E-08 |
| EN-2126904 | + | yes | 5.5E-07 |
| EN-2127523 | - | no | 1.5E-07 |
| EN-2131100 | - | no | 4.6E-08 |
| EN-2131830 | - | yes | 9.2E-08 |
| EN-2131836 | + | yes | 1.1E-07 |
| EN-2133333 | + | no | 2.8E-07 |
| EN-2133744 | - | no | 2.6E-07 |
| EN-2134455 | - | yes | 4.6E-08 |
| EN-2135934 | - | no | 0.0E+00 |
| EN-2137001 | - | no | 7.5E-07 |
| EN-2137322 | - | no | 1.5E-07 |
| EN-2137393 | - | no | 2.6E-07 |
| EN-2137785 | - | yes | 0.0E+00 |
| EN-2138662 | - | no | 4.2E-07 |
| EN-2141337 | + | no | 2.2E-07 |
| EN-2141355 | - | yes | 2.0E-07 |
| EN-2141473 | + | no | 5.2E-07 |
| EN-2142144 | - | no | 3.1E-08 |
| EN-2142414 | + | no | 1.6E-08 |
| EN-2142941 | + | no | 0.0E+00 |
| EN-2143740 | - | no | 0.0E+00 |
| EN-2144189 | - | no | 0.0E+00 |
| EN-2144312 | + | yes | 0.0E+00 |
| EN-2145840 | - | yes | 1.5E-06 |
| EN-2145940 | + | no | 1.1E-07 |
| EN-2149165 | - | no | 0.0E+00 |
| EN-2149274 | + | yes | 2.4E-07 |
| EN-2149831 | - | no | 2.2E-06 |
| EN-2150456-59 | - | yes | 2.0E-06 |
| EN-2151019 | - | no | 3.8E-07 |
| EN-2151584 | - | no | 0.0E+00 |
| EN-2151622-24 | - | no | 2.0E-07 |
| EN-2152581 | - | no | 2.3E-07 |
| EN-2152766 | - | no | 0.0E+00 |
| EN-2153077 | + | no | 0.0E+00 |
| EN-2153840 | + | no | 1.4E-06 |
| EN-2153965 | + | yes | 0.0E+00 |
| EN-2154191 | + | no | 1.1E-07 |
| EN-2154352 | + | no | 1.1E-06 |
| EN-2154773-76 | - | yes | 0.0E+00 |
| EN-2155086 | + | yes | 0.0E+00 |
| EN-2155933 | + | no | 0.0E+00 |
| EN-2156555-58 | + | yes | 1.4E-06 |
| EN-2156744 | + | no | 0.0E+00 |
| EN-2156860 | + | no | 1.0E-06 |
| EN-2157368 | + | no | 2.4E-06 |
| EN-2157525 | - | no | 2.0E-07 |
| EN-2158624 | + | no | 4.4E-07 |
| EN-2159313 | - | no | 9.7E-07 |
| EN-2160342 | - | yes | 4.6E-08 |
| EN-2160448 | + | yes | 4.7E-08 |
| EN-2161517 | - | no | 5.8E-07 |
| EN-2162999 | - | no | 0.0E+00 |
| EN-2164543 | - | no | 3.1E-08 |
| EN-2164600 | - | no | 6.2E-08 |
| EN-2165550 | - | no | 3.1E-07 |
| EN-2166446 | - | no | 9.2E-08 |
| EN-2166465 | + | no | 0.0E+00 |
| EN-2166726 | + | no | 1.6E-08 |
| EN-2168901 | - | yes | 2.3E-07 |
| EN-2168990 | + | yes | 3.0E-06 |
| EN-2169757 | - | no | 1.5E-08 |
| EN-2170646 | + | no | 7.9E-08 |
| EN-2170830 | - | no | 1.7E-07 |
| EN-2172422 | - | yes | 6.9E-07 |
| EN-2172733 | - | no | 6.2E-08 |
| EN-2173139 | - | no | 2.3E-07 |
| EN-2173552 | + | no | 0.0E+00 |
| EN-2174714 | - | yes | 2.6E-07 |
| EN-2174792 | + | yes | 1.3E-07 |
| LE-2175035 | + | only | n/a |
| EN-2177121 | + | no | 0.0E+00 |
| EN-2179582 | - | no | 2.5E-07 |
| EN-2181571 | - | yes | 7.5E-07 |
| EN-2183669 | + | no | 1.1E-07 |
| EN-2183669 | - | no | 0.0E+00 |
| EN-2185789 | - | no | 8.8E-07 |
| EN-2186422 | - | no | 4.6E-08 |
| EN-2186503 | - | no | 4.6E-08 |
| EN-2186514 | - | no | 4.6E-08 |
| EN-2186526 | + | yes | 5.7E-07 |
| EN-2187418 | - | no | 0.0E+00 |
| EN-2187493 | + | no | 0.0E+00 |
| EN-2187838 | - | yes | 6.2E-08 |
| EN-2188857 | - | no | 6.2E-08 |
| EN-2189845 | - | yes | 4.6E-08 |
| EN-2189897 | + | no | 7.1E-07 |
| EN-2189899 | - | no | 1.5E-07 |
| EN-2191151 | - | no | 0.0E+00 |
| EN-2191999 | - | no | 4.6E-08 |
| EN-2192348 | - | no | 1.4E-07 |
| EN-2192503 | + | no | 0.0E+00 |
| EN-2194725 | - | yes | 6.9E-07 |
| EN-2194985 | + | no | 1.2E-06 |
| EN-2195879 | + | yes | 3.3E-07 |
| EN-2196429 | - | no | 1.1E-07 |
| EN-2196995 | + | no | 1.6E-08 |
| EN-2197097 | - | no | 1.1E-07 |
| EN-2197625 | - | yes | 5.5E-06 |
| EN-2198114 | - | yes | 3.1E-08 |
| EN-2198578 | - | no | 0.0E+00 |
| EN-2199203 | - | no | 6.0E-07 |
| EN-2200064 | + | yes | 2.7E-06 |
| LE-2200671 | - | only | n/a |
| EN-2201207 | + | no | 5.5E-07 |
| EN-2204446-48 | + | no | 0.0E+00 |
| EN-2205790 | + | no | 0.0E+00 |
| EN-2207733 | + | no | 2.4E-07 |
| EN-2208447 | - | no | 1.5E-07 |
| EN-2210193 | - | no | 1.1E-07 |
| EN-2210600 | - | no | 4.2E-07 |
| EN-2211144-52 | - | no | 2.3E-07 |
| EN-2211246 | - | no | 0.0E+00 |
| EN-2211424 | - | no | 0.0E+00 |
| EN-2211462 | - | no | 4.2E-07 |
| EN-2212299 | + | no | 2.4E-07 |
| EN-2212625 | - | no | 0.0E+00 |
| EN-2212905 | - | no | 0.0E+00 |
| EN-2215595 | - | no | 8.0E-06 |
| EN-2215665 | - | yes | 6.9E-07 |
| EN-2216448 | + | no | 0.0E+00 |
| EN-2216774 | - | no | 0.0E+00 |
| EN-2216795 | - | no | 0.0E+00 |
| EN-2216894 | - | no | 8.2E-07 |
| EN-2217337 | - | yes | 2.2E-07 |
| EN-2217429 | - | no | 1.5E-08 |
| EN-2217467 | - | no | 3.1E-08 |
| EN-2217533 | - | no | 1.5E-07 |
| EN-2217596 | + | no | 0.0E+00 |
| EN-2218457 | - | no | 7.8E-07 |
| EN-2218480 | + | no | 2.0E-07 |
| EN-2218665 | - | no | 0.0E+00 |
| EN-2218928 | + | yes | 1.7E-07 |
| EN-2219042 | + | no | 0.0E+00 |
| EN-2220517-23 | - | no | 1.5E-07 |
| EN-2220533 | - | yes | 1.2E-07 |
| EN-2220876 | - | no | 2.3E-07 |
| EN-2221842 | - | no | 4.9E-07 |
| EN-2221927 | - | no | 6.9E-07 |
| EN-2222822 | - | no | 7.8E-07 |
| EN-2223823 | - | no | 9.2E-08 |
| EN-2224186 | + | no | 1.6E-08 |
| EN-2224321 | - | no | 4.6E-08 |
| EN-2224743 | - | yes | 8.8E-07 |
| EN-2225352-55 | + | yes | 0.0E+00 |
| EN-2225862 | - | no | 3.1E-08 |
| EN-2225884 | - | no | 2.2E-07 |
| EN-2226793 | + | no | 3.1E-08 |
| EN-2227121 | + | no | 6.8E-07 |
| EN-2228023 | - | no | 4.6E-08 |
| EN-2228960 | + | yes | 1.3E-07 |
| EN-2229434 | + | no | 1.0E-06 |
| EN-2230073 | - | no | 0.0E+00 |
| EN-2234304 | - | no | 1.5E-08 |
| EN-2234638-40 | - | no | 6.2E-08 |
| EN-2235140 | - | no | 2.2E-07 |
| EN-2235211 | + | no | 5.5E-08 |
| EN-2237074 | - | no | 0.0E+00 |
| EN-2237711 | - | no | 3.1E-08 |
| EN-2239224 | + | no | 0.0E+00 |
| EN-2239553 | - | no | 6.3E-07 |
| EN-2241422-24 | - | no | 3.1E-08 |
| EN-2243780 | - | no | 1.5E-08 |
| EN-2245174 | - | no | 6.2E-08 |
| EN-2245245 | - | no | 0.0E+00 |
| EN-2245892 | + | no | 4.7E-08 |
| EN-2246668 | - | no | 0.0E+00 |
| EN-2247772 | - | no | 0.0E+00 |
| EN-2248011 | - | no | 4.6E-08 |
| EN-2250288 | - | no | 7.5E-07 |
| EN-2250333 | + | yes | 0.0E+00 |
| EN-2250485 | + | no | 0.0E+00 |
| EN-2251239 | + | no | 0.0E+00 |
| EN-2251917-19 | + | yes | 2.4E-07 |
| EN-2251939 | - | no | 0.0E+00 |
| EN-2252497 | - | no | 2.5E-07 |
| EN-2252921 | - | no | 0.0E+00 |
| EN-2253842 | - | no | 1.1E-07 |
| EN-2254968 | - | no | 0.0E+00 |
| EN-2254985-92 | + | yes | 3.1E-08 |
| EN-2255137 | - | yes | 3.1E-08 |
| EN-2255680 | - | no | 9.4E-07 |
| EN-2256043 | + | no | 7.9E-08 |
| EN-2257224 | + | no | 1.6E-08 |
| EN-2260551 | + | no | 4.7E-08 |
| EN-2261663 | + | no | 3.1E-08 |
| EN-2262383 | + | no | 0.0E+00 |
| EN-2262676 | - | no | 0.0E+00 |
| EN-2262749 | + | yes | 5.8E-06 |
| LE-2262758 | + | only | n/a |
| EN-2263621 | - | yes | 0.0E+00 |
| EN-2263927 | - | no | 1.2E-07 |
| EN-2264056 | - | no | 0.0E+00 |
| EN-2264608 | + | no | 0.0E+00 |
| EN-2265598-00 | + | no | 0.0E+00 |
| EN-2266430 | - | yes | 2.5E-06 |
| EN-2266805 | + | no | 1.1E-07 |
| EN-2268289 | - | no | 6.2E-08 |
| EN-2269071 | + | no | 1.6E-08 |
| EN-2269893 | + | yes | 4.7E-08 |
| EN-2270109 | - | no | 1.5E-08 |
| LE-2270192 | - | only | n/a |
| EN-2270276-79 | + | yes | 2.4E-07 |
| EN-2271432 | + | no | 0.0E+00 |
| EN-2271805 | - | no | 0.0E+00 |
| EN-2273159 | + | yes | 4.4E-05 |
| EN-2275501 | + | yes | 0.0E+00 |
| EN-2275841 | - | no | 1.7E-07 |
| EN-2276914 | + | no | 3.1E-08 |
| EN-2278458 | + | no | 0.0E+00 |
| EN-2279181 | - | no | 1.5E-08 |
| EN-2279633 | - | yes | 1.5E-08 |
| EN-2279687 | - | no | 1.1E-07 |
| EN-2279850 | + | no | 2.8E-07 |
| EN-2279866-68 | - | no | 1.5E-07 |
| EN-2279887-89 | - | yes | 1.1E-07 |
| EN-2280058-61 | + | yes | 1.1E-07 |
| EN-2280098 | - | no | 1.1E-07 |
| EN-2281766 | - | yes | 9.2E-08 |
| EN-2281775 | - | yes | 6.0E-07 |
| EN-2281784 | + | yes | 0.0E+00 |
| EN-2281797-99 | + | no | 4.4E-07 |
| EN-2283081 | - | no | 4.6E-08 |
| EN-2284464 | + | no | 1.3E-07 |
| EN-2285027 | + | yes | 9.4E-08 |
| EN-2286407 | - | yes | 1.5E-08 |
| EN-2288246 | + | no | 7.9E-08 |
| EN-2289234 | - | no | 4.6E-08 |
| EN-2289253 | + | yes | 0.0E+00 |
| EN-2289424 | - | no | 1.5E-07 |
| EN-2289899 | - | no | 2.2E-07 |
| EN-2289915-17 | - | yes | 2.4E-06 |
| EN-2291317 | + | no | 9.4E-08 |
| EN-2291788 | + | no | 6.8E-07 |
| EN-2294577 | + | yes | 0.0E+00 |
| EN-2295232 | + | no | 1.6E-08 |
| EN-2296008 | - | no | 3.1E-08 |
| EN-2296182 | + | no | 0.0E+00 |
| EN-2296499 | + | no | 0.0E+00 |
| EN-2297782 | - | no | 6.2E-08 |
| EN-2297980 | - | no | 2.0E-07 |
| EN-2298645 | - | no | 0.0E+00 |
| EN-2298959 | + | no | 4.4E-07 |
| EN-2299004 | - | no | 0.0E+00 |
| EN-2299505 | + | yes | 8.4E-06 |
| EN-2300471 | + | no | 2.4E-07 |
| EN-2300667 | + | yes | 0.0E+00 |
| EN-2301917 | - | no | 1.7E-07 |
| EN-2302239 | + | yes | 9.4E-08 |
| EN-2302279-83 | + | no | 2.5E-06 |
| EN-2303013 | - | no | 0.0E+00 |
| EN-2303172 | - | no | 6.2E-08 |
| EN-2304258 | + | no | 1.6E-08 |
| EN-2305442 | - | yes | 1.4E-06 |
| EN-2305541 | - | no | 4.6E-08 |
| EN-2305548 | + | yes | 2.4E-07 |
| EN-2306399 | - | no | 1.5E-08 |
| EN-2306582 | - | no | 6.2E-08 |
| EN-2307124 | - | no | 1.1E-07 |
| EN-2307288 | - | no | 0.0E+00 |
| EN-2307343 | - | no | 0.0E+00 |
| EN-2307511 | - | no | 1.2E-07 |
| EN-2309329 | - | no | 0.0E+00 |
| EN-2311086 | - | yes | 6.9E-07 |
| EN-2311206 | + | yes | 2.8E-07 |
| EN-2311256 | - | no | 2.0E-07 |
| EN-2311824-26 | - | no | 0.0E+00 |
| EN-2311904 | + | no | 5.2E-07 |
| EN-2312130 | - | no | 1.4E-07 |
| EN-2315801 | - | no | 8.8E-07 |
| EN-2316091 | + | no | 2.5E-07 |
| EN-2316817 | + | yes | 1.6E-08 |
| EN-2317705 | - | yes | 7.7E-08 |
| EN-2317832 | + | yes | 4.7E-08 |
| EN-2318033 | - | no | 1.5E-07 |
| EN-2318378 | + | no | 1.6E-08 |
| EN-2320318 | - | no | 4.2E-07 |
| LE-2320432 | + | only | n/a |
| EN-2320599 | + | no | 0.0E+00 |
| EN-2320755 | - | no | 0.0E+00 |
| LE-2320773 | + | only | n/a |
| EN-2321269 | - | no | 0.0E+00 |
| EN-2321409 | - | no | 4.6E-08 |
| EN-2321617-23 | - | yes | 9.2E-08 |
| EN-2321635 | + | yes | 1.1E-07 |
| EN-2322168-70 | - | no | 6.2E-08 |
| EN-2322805 | - | no | 6.2E-08 |
| EN-2322880 | + | no | 4.2E-07 |
| EN-2322990 | + | no | 1.1E-07 |
| EN-2323206 | - | no | 3.1E-08 |
| EN-2325432-35 | - | no | 6.9E-07 |
| EN-2326346 | - | no | 9.2E-08 |
| EN-2326399 | - | no | 2.3E-07 |
| EN-2326417 | + | yes | 4.7E-08 |
| EN-2327366 | - | no | 6.2E-08 |
| EN-2328729 | + | no | 9.4E-08 |
| EN-2330781 | + | yes | 0.0E+00 |
| EN-2331138-40 | - | no | 1.1E-07 |
| EN-2331239 | - | yes | 1.1E-07 |
| EN-2331334 | + | yes | 2.8E-07 |
| EN-2331484 | + | no | 0.0E+00 |
| EN-2332046 | - | no | 0.0E+00 |
| EN-2332613-15 | - | no | 1.8E-07 |
| EN-2332963 | - | no | 0.0E+00 |
| EN-2333711 | - | no | 0.0E+00 |
| EN-2334794 | - | yes | 0.0E+00 |
| EN-2334810-12 | + | yes | 4.3E-06 |
| EN-2335106 | + | yes | 2.9E-06 |
| EN-2335832 | - | no | 1.5E-08 |
| EN-2336583 | + | no | 3.1E-08 |
| EN-2337919 | - | no | 6.2E-08 |
| EN-2338912 | + | yes | 3.9E-07 |
| EN-2340787 | + | no | 1.6E-08 |
| EN-2341818 | - | no | 1.1E-07 |
| EN-2342821 | + | yes | 1.6E-08 |
| EN-2343229 | + | yes | 1.1E-07 |
| EN-2344171-73 | - | no | 1.8E-07 |
| EN-2344741 | - | no | 1.5E-07 |
| EN-2344945 | + | yes | 1.1E-07 |
| EN-2345548 | - | no | 1.5E-05 |
| EN-2345611 | + | yes | 0.0E+00 |
| LE-2345704 | - | only | n/a |
| EN-2346154 | + | no | 0.0E+00 |
| EN-2346390-92 | + | yes | 0.0E+00 |
| EN-2349097 | - | no | 7.1E-07 |
| EN-2350792 | + | no | 0.0E+00 |
| EN-2351156 | - | no | 6.9E-07 |
| EN-2351195 | - | no | 2.5E-07 |
| EN-2352588 | + | no | 0.0E+00 |
| EN-2353176 | + | no | 0.0E+00 |
| EN-2353406 | - | yes | 0.0E+00 |
| EN-2353647 | - | no | 2.4E-07 |
| EN-2354015 | + | yes | 4.7E-07 |
| EN-2354270 | - | no | 1.5E-08 |
| EN-2354525 | + | yes | 4.4E-07 |
| EN-2356880 | - | no | 2.8E-06 |
| LE-2357152 | + | only | n/a |
| EN-2357455 | - | no | 1.5E-07 |
| EN-2359182 | - | yes | 7.4E-07 |
| EN-2359188 | + | no | 1.1E-07 |
| EN-2359454 | + | yes | 1.3E-07 |
| EN-2360505 | - | no | 2.6E-07 |
| EN-2360736 | - | no | 6.2E-08 |
| EN-2360796 | - | no | 0.0E+00 |
| EN-2361843 | - | yes | 0.0E+00 |
| EN-2362484 | - | no | 0.0E+00 |
| EN-2362998 | - | no | 2.0E-07 |
| EN-2363042 | + | no | 1.1E-07 |
| EN-2365229 | - | no | 4.6E-08 |
| EN-2365391 | - | yes | 1.3E-05 |
| EN-2365532 | - | no | 0.0E+00 |
| EN-2365576 | + | yes | 7.9E-08 |
| EN-2365794 | - | no | 6.2E-08 |
| EN-2366480-82 | + | no | 3.3E-07 |
| EN-2366963 | + | no | 1.7E-07 |
| EN-2367516 | - | no | 1.8E-07 |
| LE-2367840 | + | only | n/a |
| EN-2368231 | - | no | 3.8E-07 |
| EN-2369475 | - | no | 1.7E-07 |
| EN-2369956 | + | no | 0.0E+00 |
| EN-2369976 | - | no | 9.2E-08 |
| EN-2370037 | - | yes | 1.5E-08 |
| EN-2371033 | - | no | 3.1E-07 |
| EN-2371166 | + | no | 0.0E+00 |
| EN-2371343-45 | - | yes | 5.4E-07 |
| EN-2371527-31 | + | yes | 0.0E+00 |
| EN-2371918 | + | no | 4.7E-08 |
| EN-2372273 | + | no | 0.0E+00 |
| EN-2375809 | - | no | 1.6E-06 |
| EN-2376947-50 | + | no | 1.6E-08 |
| EN-2377361 | - | no | 1.5E-07 |
| EN-2377455 | + | no | 6.6E-06 |
| EN-2378027 | - | no | 0.0E+00 |
| EN-2378688 | - | yes | 0.0E+00 |
| EN-2379417 | + | no | 0.0E+00 |
| EN-2380025 | - | no | 6.2E-08 |
| EN-2380556 | + | yes | 5.5E-07 |
| EN-2380942-44 | - | yes | 1.5E-08 |
| EN-2381698 | - | yes | 2.0E-07 |
| EN-2383368 | - | no | 0.0E+00 |
| EN-2383635 | + | yes | 1.3E-07 |
| EN-2383918 | - | yes | 4.8E-07 |
| EN-2386888 | + | yes | 1.6E-08 |
| EN-2387070 | - | no | 2.6E-06 |
| EN-2387163 | - | yes | 9.5E-07 |
| EN-2387346 | + | yes | 7.9E-08 |
| EN-2387414 | - | no | 2.6E-06 |
| EN-2387978 | - | no | 0.0E+00 |
| EN-2388012 | + | yes | 5.2E-06 |
| EN-2388291 | + | no | 0.0E+00 |
| EN-2389080 | - | no | 7.8E-07 |
| EN-2389445 | - | no | 1.1E-07 |
| EN-2390717 | - | no | 5.2E-07 |
| EN-2391179 | + | no | 5.0E-07 |
| EN-2391844 | - | no | 6.2E-08 |
| EN-2393235 | + | no | 0.0E+00 |
| EN-2393331 | - | yes | 6.2E-08 |
| EN-2393823-25 | - | yes | 1.5E-08 |
| EN-2394380 | - | no | 0.0E+00 |
| EN-2398124 | - | no | 1.5E-07 |
| EN-2398375 | - | no | 6.2E-08 |
| EN-2399192 | - | no | 2.3E-07 |
| EN-2400318 | - | no | 2.2E-07 |
| EN-2401053 | + | no | 4.7E-08 |
| EN-2402670 | - | no | 1.8E-07 |
| EN-2404339 | - | no | 0.0E+00 |
| EN-2404554 | - | no | 1.4E-06 |
| EN-2404616-19 | + | no | 1.1E-07 |
| EN-2405112 | + | no | 1.6E-08 |
| EN-2405386 | - | no | 1.7E-07 |
| EN-2406466 | + | no | 4.7E-08 |
| EN-2407396 | + | no | 1.6E-08 |
| EN-2407762 | - | no | 9.2E-08 |
| EN-2408177 | + | no | 0.0E+00 |
| EN-2408606 | - | no | 9.4E-06 |
| EN-2409056 | + | no | 0.0E+00 |
| EN-2410592 | - | yes | 4.6E-08 |
| EN-2411518-20 | - | no | 3.1E-08 |
| EN-2413146 | + | no | 1.1E-07 |
| EN-2413307 | + | no | 0.0E+00 |
| EN-2413310 | - | no | 9.5E-06 |
| EN-2413797 | - | yes | 0.0E+00 |
| LE-2413798 | - | only | n/a |
| EN-2414154 | + | no | 0.0E+00 |
| EN-2414591 | - | no | 1.8E-07 |
| EN-2415750 | - | no | 0.0E+00 |
| EN-2416184 | - | no | 0.0E+00 |
| EN-2417130 | - | no | 0.0E+00 |
| EN-2419084 | - | no | 1.5E-08 |
| EN-2419487 | - | no | 6.2E-08 |
| EN-2419928 | - | yes | 1.7E-07 |
| EN-2420039 | + | yes | 2.4E-07 |
| EN-2420712-15 | + | no | 4.4E-07 |
| EN-2422366 | - | no | 0.0E+00 |
| EN-2423541 | - | yes | 0.0E+00 |
| EN-2425642 | + | no | 1.6E-08 |
| EN-2425800 | + | no | 2.2E-07 |
| EN-2425941 | + | no | 9.4E-08 |
| EN-2427001 | - | yes | 9.2E-08 |
| EN-2427257 | - | no | 6.2E-08 |
| EN-2427290 | - | yes | 1.1E-07 |
| EN-2427332 | - | no | 5.8E-07 |
| EN-2427437-40 | + | yes | 6.0E-07 |
| EN-2428848 | + | no | 6.3E-06 |
| EN-2429422 | - | no | 0.0E+00 |
| EN-2429516 | - | yes | 7.7E-08 |
| EN-2430005-08 | + | yes | 3.5E-07 |
| LE-2431215 | - | only | n/a |
| EN-2431470 | + | no | 5.8E-07 |
| EN-2432327 | + | yes | 0.0E+00 |
| EN-2432651 | + | no | 0.0E+00 |
| EN-2433392 | + | no | 7.9E-08 |
| EN-2434696 | + | no | 1.1E-07 |
| EN-2435527 | - | no | 4.6E-08 |
| EN-2436649 | - | no | 9.2E-08 |
| EN-2436831 | - | yes | 6.2E-08 |
| EN-2436906 | + | no | 1.6E-08 |
| EN-2437568 | - | no | 6.2E-08 |
| EN-2438184 | - | no | 2.0E-07 |
| EN-2438837 | - | no | 0.0E+00 |
| EN-2439433 | - | no | 1.5E-07 |
| EN-2440135 | + | no | 0.0E+00 |
| EN-2440358 | - | no | 0.0E+00 |
| EN-2440569 | - | no | 1.5E-08 |
| EN-2440667 | + | yes | 1.3E-07 |
| EN-2440723 | + | no | 2.4E-06 |
| EN-2440733 | + | no | 0.0E+00 |
| EN-2441166 | - | no | 4.6E-08 |
| EN-2441421 | - | no | 4.6E-08 |
| EN-2441448 | - | yes | 0.0E+00 |
| EN-2441486 | + | yes | 3.3E-07 |
| EN-2442239 | - | no | 1.5E-07 |
| EN-2442623 | - | no | 2.9E-06 |
| EN-2443468-70 | - | no | 1.5E-08 |
| EN-2445373 | - | no | 0.0E+00 |
| EN-2446357 | - | no | 0.0E+00 |
| EN-2447275 | - | no | 6.0E-07 |
| EN-2447361 | + | yes | 0.0E+00 |
| EN-2447382 | - | no | 0.0E+00 |
| EN-2447437 | - | no | 0.0E+00 |
| EN-2447463 | + | no | 5.0E-07 |
| EN-2447466 | - | yes | 2.0E-07 |
| EN-2449202 | + | no | 3.3E-07 |
| EN-2449235 | - | no | 0.0E+00 |
| EN-2449519 | - | no | 3.1E-08 |
| EN-2450593 | - | yes | 1.8E-07 |
| EN-2450920-29 | + | no | 2.1E-06 |
| EN-2452549 | + | no | 1.7E-07 |
| EN-2453150 | + | yes | 2.8E-07 |
| EN-2455065 | - | no | 1.5E-08 |
| EN-2455634 | - | no | 8.3E-07 |
| EN-2455694-98 | - | no | 5.8E-06 |
| EN-2455824 | - | no | 1.3E-06 |
| EN-2457102 | + | no | 9.4E-08 |
| EN-2458265 | + | no | 0.0E+00 |
| EN-2459371 | + | no | 1.6E-08 |
| EN-2459497 | - | yes | 0.0E+00 |
| EN-2459621 | + | yes | 1.1E-07 |
| EN-2459675 | - | no | 1.5E-07 |
| EN-2460665 | - | no | 3.8E-07 |
| EN-2461107 | + | yes | 0.0E+00 |
| EN-2462816 | + | no | 4.7E-08 |
| EN-2464727 | + | no | 7.9E-08 |
| EN-2465677 | - | no | 3.0E-06 |
| EN-2465737-41 | - | no | 6.8E-06 |
| EN-2465867-75 | - | yes | 4.6E-08 |
| EN-2466696 | - | yes | 9.2E-08 |
| EN-2467202 | - | no | 0.0E+00 |
| EN-2467500 | + | no | 3.1E-08 |
| EN-2467611 | + | no | 2.4E-07 |
| EN-2470429 | - | no | 0.0E+00 |
| EN-2470485 | - | no | 2.5E-07 |
| EN-2475031 | - | yes | 2.3E-07 |
| EN-2475608-10 | + | yes | 2.7E-06 |
| EN-2476528 | + | no | 1.6E-08 |
| EN-2477978-82 | + | no | 9.4E-08 |
| EN-2479294 | + | no | 0.0E+00 |
| EN-2481479 | - | no | 1.4E-06 |
| EN-2481761 | - | no | 2.0E-07 |
| EN-2483147 | - | no | 1.5E-08 |
| EN-2484124 | - | no | 3.1E-08 |
| EN-2484690 | - | no | 1.5E-07 |
| EN-2484795 | - | yes | 4.6E-08 |
| LE-2484796 | - | only | n/a |
| EN-2484814 | + | yes | 0.0E+00 |
| EN-2484847 | - | no | 9.2E-07 |
| EN-2485931 | - | no | 0.0E+00 |
| EN-2486372 | - | no | 4.6E-08 |
| EN-2486477 | + | no | 0.0E+00 |
| EN-2486730 | + | no | 0.0E+00 |
| EN-2486987 | + | yes | 4.2E-07 |
| EN-2487944 | - | no | 0.0E+00 |
| EN-2489645 | + | yes | 5.5E-07 |
| EN-2489657 | - | no | 9.1E-07 |
| EN-2489738 | + | no | 1.1E-07 |
| EN-2492092-94 | - | yes | 4.2E-07 |
| EN-2493898 | - | yes | 1.1E-07 |
| EN-2493975 | + | no | 4.5E-06 |
| EN-2494395-98 | + | no | 3.0E-07 |
| EN-2494705 | - | no | 0.0E+00 |
| EN-2495260 | - | no | 2.0E-07 |
| EN-2495393 | + | no | 2.4E-07 |
| LE-2496196 | - | only | n/a |
| EN-2496291 | + | no | 3.1E-08 |
| EN-2496348 | + | yes | 2.4E-07 |
| EN-2496563 | + | no | 0.0E+00 |
| EN-2497350 | - | no | 2.2E-07 |
| EN-2498220 | + | no | 1.1E-07 |
| EN-2499213 | + | no | 1.6E-08 |
| EN-2500394 | + | yes | 1.7E-06 |
| EN-2501221 | - | no | 6.2E-08 |
| EN-2501695 | + | no | 0.0E+00 |
| EN-2502108 | + | no | 1.4E-07 |
| EN-2502432 | + | no | 4.7E-08 |
| EN-2503142-44 | - | yes | 0.0E+00 |
| EN-2504800-02 | + | no | 1.6E-08 |
| EN-2504880 | + | no | 0.0E+00 |
| EN-2505019 | + | yes | 0.0E+00 |
| EN-2505058 | - | no | 1.5E-08 |
| EN-2506283 | - | no | 0.0E+00 |
| EN-2507431 | + | no | 0.0E+00 |
| EN-2507565 | - | yes | 0.0E+00 |
| EN-2507574 | - | no | 0.0E+00 |
| EN-2507693 | - | no | 0.0E+00 |
| EN-2508074 | - | yes | 0.0E+00 |
| EN-2508087 | - | no | 1.5E-08 |
| EN-2508093 | + | no | 0.0E+00 |
| EN-2508177 | + | yes | 0.0E+00 |
| EN-2508932 | - | no | 0.0E+00 |
| EN-2509226 | + | no | 0.0E+00 |
| EN-2510998 | - | no | 1.5E-06 |
| EN-2511818 | + | no | 0.0E+00 |
| EN-2512430 | - | yes | 0.0E+00 |
| EN-2514339 | - | no | 2.6E-06 |
| EN-2514463 | - | yes | 9.2E-08 |
| EN-2514832 | - | no | 0.0E+00 |
| EN-2515432 | - | yes | 6.2E-08 |
| EN-2515981 | - | no | 0.0E+00 |
| EN-2517010 | + | no | 5.7E-07 |
| EN-2517261 | + | no | 7.6E-07 |
| EN-2517849 | - | no | 0.0E+00 |
| EN-2518466 | + | no | 0.0E+00 |
| EN-2518656 | - | no | 0.0E+00 |
| EN-2518692 | - | no | 7.8E-07 |
| EN-2518962 | - | no | 0.0E+00 |
| EN-2519663 | - | no | 1.1E-06 |
| EN-2519916 | - | no | 1.1E-07 |
| EN-2521542 | + | no | 9.4E-08 |
| EN-2522216 | + | no | 0.0E+00 |
| EN-2522888 | - | yes | 6.2E-08 |
| EN-2524829-32 | - | yes | 0.0E+00 |
| EN-2524851 | + | no | 2.4E-07 |
| EN-2526282 | - | yes | 7.8E-07 |
| EN-2527852 | - | yes | 1.1E-07 |
| EN-2530926 | - | no | 0.0E+00 |
| EN-2531823 | + | no | 0.0E+00 |
| EN-2532075 | + | no | 0.0E+00 |
| EN-2533246 | - | no | 3.9E-06 |
| EN-2533257 | - | no | 3.1E-08 |
| EN-2536459 | - | no | 4.6E-08 |
| EN-2536747 | - | no | 1.1E-07 |
| EN-2538394 | - | no | 1.1E-07 |
| EN-2538804-09 | + | yes | 4.7E-08 |
| EN-2539291 | - | no | 0.0E+00 |
| EN-2539297 | + | yes | 9.4E-08 |
| EN-2539817 | + | no | 1.6E-08 |
| EN-2540665 | - | no | 0.0E+00 |
| EN-2540833 | - | no | 3.8E-07 |
| EN-2541945 | + | no | 1.6E-08 |
| EN-2542451 | - | no | 0.0E+00 |
| EN-2543077 | - | yes | 0.0E+00 |
| LE-2544392 | + | only | n/a |
| EN-2545479 | + | no | 0.0E+00 |
| EN-2546077 | - | no | 1.4E-06 |
| EN-2546149 | + | no | 2.4E-07 |
| EN-2546803 | + | no | 9.4E-08 |
| EN-2546839 | - | no | 0.0E+00 |
| EN-2548078 | + | no | 0.0E+00 |
| EN-2549113-15 | - | no | 6.2E-08 |
| EN-2549330 | + | no | 1.6E-08 |
| EN-2549349 | - | yes | 6.9E-07 |
| EN-2549921 | - | no | 4.0E-07 |
| EN-2550787 | - | no | 0.0E+00 |
| EN-2550860 | - | no | 7.8E-07 |
| EN-2551083-86 | - | no | 0.0E+00 |
| EN-2551178 | - | no | 0.0E+00 |
| EN-2551220 | - | no | 8.0E-07 |
| EN-2551246 | - | yes | 1.1E-07 |
| EN-2551263-65 | + | yes | 1.7E-07 |
| EN-2551640 | - | no | 0.0E+00 |
| EN-2552091 | - | no | 2.5E-07 |
| EN-2552904-06 | - | no | 0.0E+00 |
| EN-2552952 | + | no | 5.0E-07 |
| EN-2552995 | - | no | 9.2E-08 |
| EN-2553082-85 | - | yes | 1.5E-08 |
| EN-2553177 | + | no | 0.0E+00 |
| EN-2553766 | - | no | 1.2E-07 |
| EN-2554471 | - | no | 1.1E-07 |
| EN-2554509 | - | no | 0.0E+00 |
| EN-2554915 | - | no | 1.2E-07 |
| EN-2555991 | - | no | 0.0E+00 |
| EN-2557138 | - | yes | 4.8E-07 |
| EN-2557206 | - | no | 1.1E-07 |
| EN-2559320 | + | no | 7.9E-08 |
| EN-2559810 | - | no | 7.8E-07 |
| EN-2560259 | - | yes | 2.2E-07 |
