# Supplementary material for: A combination of improved differential and global RNA-seq reveals pervasive transcription initiation and events in all stages of the life-cycle of functional RNAs in Propionibacterium acnes, a major contributor to wide-spread human disease
Source: BMC Genomics. 2013 Sep 14;14:620. doi: 10.1186/1471-2164-14-620 (PMC3848588; doi:10.1186/1471-2164-14-620)
Supplement: Additional file 4 — P. acnes homologues of major factors involved in RNA processing and degradation. Further details of factors involved in eubacterial RNA degradation and processing can be found in several recent reviews [9,10,16,104]. [file 1471-2164-14-620-S4.docx]

| **Factor** | **Gene** | **Description** |
| --- | --- | --- |
| RNase E/G | PPA0826 | Single strand-specific endoRNase involved in RNA degradation and the processing of stable RNAs. PPA0826 product has N-terminal extension (*cf*. *E. coli* enzymes). |
| RNase III | PPA1452 | Double strand-specific endoRNase involved in processing of rRNA and mRNA. May initiate the cleavage of some mRNAs. |
| RNase P (RNA component) | PPA0652 | EndoRNase that processes the 5’ end of tRNA. Also processes its own catalytic RNA and cuts some mRNAs. |
| RppH | PPA0342 | RNA pyrophosphohydrolase that initiates degradation of some mRNA by decapping the 5'-triphosphate end. |
| RNase J | PPA1467 | RNase with dual endo and 5′ to 3′ exo activity; has roles in the degradation of specific structural mRNAs. Does not appear to be critical for mRNA degradation in *B. subtilis*, not found in *E. coli.* |
| RNase Y | PPA1014 | Endonuclease involved in the degradation of mRNA in *B. subtilis*, not found in *E. coli.* |
| PNPase | PPA1471 | 3' to 5' exoRNase and 3'-terminal oligonucleotide polymerase. Functions in the degradation of various mRNAs and tRNA maturation. |
| oligoRNase | PPA1642 | Processive 3' to 5' exoRNase specific for short oligoribonucleotides. Final enzyme in degradation of RNAs to mononucleotides in *E. coli*. |
| tRNA nucleotidyl-transferase | PPA2301 | Responsible for adding CCA to 3' ends of tRNA for which this motif is not encoded. |
| RNase PH | PPA1674 | 3' to 5' exoRNase involved in 3' trimming of tRNAs. |
| RNase D | PPA1063 | 3' to 5' exoRNase involved in the 3' processing of various stable RNA molecules. |
|  | | |
| tRNase Z | none | EndoRNase that can generate mature 3' end of tRNA. |
| nanoRNase | none | Functionally equivalent to oligoRNase, found in *B. subtilis.* |
| RNase BN | none | 3' to 5' exoRNase involved in 3' trimming of tRNAs as well as various short unstructured RNAs. |
| RNase T | none | 3' to 5' exoRNase responsible for 3' trimming of many stable RNAs, including tRNAs and 5S rRNA. Can compensate for lack of other 3’-5’ exonucleases in tRNA maturation. |
| RNase II and RNase R | none | 3’-5’ exoRNase that cleaves RNA from the 3' end to produce ribonucleoside 5'-monophosphates. |
