# Supplementary material for: A combination of improved differential and global RNA-seq reveals pervasive transcription initiation and events in all stages of the life-cycle of functional RNAs in Propionibacterium acnes, a major contributor to wide-spread human disease
Source: BMC Genomics. 2013 Sep 14;14:620. doi: 10.1186/1471-2164-14-620 (PMC3848588; doi:10.1186/1471-2164-14-620)
Supplement: Additional file 5 — Genes with altered expression as a consequence of potassium downshift. The microarray data obtained for each of the two duplicate cultures was analysed using M-A (ratio-intensity) scatterplots (data not shown). The vast majority of the points in each comparison (with vs. without downshift) were contained within boundaries described by the equation μ ± 3σ, where μ and σ are the average and standard deviation, respectively, of M in a moving window of 5,000 data-points sorted in ascending order of A [22,23]. The genes listed in this table were outside the boundaries in both of the two comparisons. The microarray data was also analysed using an online version of Rank Product algorithm [26], which detects differentially regulated genes in replicated microarray experiments. Overall the analysis of the M-A scatterplots appears to have been more sensitive. In two cases, it identified all of the genes in a cluster with related function, while Rank Product did not (see PPA1287-90 and PPA1758-60). It should be noted that genes linked to iron homeostasis were detected, e.g. genes encoding the ferrous iron transport proteins A and B (PPA1676 and PPA1677, respectively), and the production of a peptide-based iron chelators (PPA1287-1291). The potassium used to culture P. acnes was contaminated with trace amounts of iron. Thus, removing the potassium also removed a source of iron. P-values are for change in expression. +Transcription starts within PPA0114. [file 1471-2164-14-620-S5.docx]

| **Gene** | **Function** | **Fold change** | **p-value** |
| --- | --- | --- | --- |
| PPA0114**^+^** | hypothetical protein | +201.4 | 0.00E+00 |
| PPA0115 | potassium-transporting ATPase subunit A | +242.3 | 0.00E+00 |
| PPA0116 | potassium-transporting ATPase subunit B | +224 | 0.00E+00 |
| PPA0117 | potassium-transporting ATPase subunit C | +69.1 | 3.27E-06 |
| PPA0118 | two-component sensor, KdpD | +19.5 | 1.74E-05 |
| PPA0119 | two-component response regulator, KdpE | +33.9 | 7.62E-06 |
| PPA0120 | hypothetical protein | +5.9 | 4.35E-05 |
| PPA0476 | glucosamine-6-phosphate isomerase | +4.6 | 1.28E-04 |
| PPA0667 | hypothetical protein | +2.5 | 7.12E-04 |
| PPA1091 | hypothetical membrane associated protein | +3.1 | 3.37E-04 |
| PPA1092 | protein with Sua5/YciO/YrdC domain | +2.8 | 7.18E-04 |
| PPA1093 | 5`-methylthioadenosine/S-adenosylhomocysteine nuclosidase | +2.4 | 1.56E-03 |
| PPA1105 | phosphoglucomutase | +2.1 | 4.80E-03 |
| PPA1224 | putative glycerophosphoryl diester phosphodiesterase | +2.9 | 3.40E-04 |
| PPA1287 | non-ribosomal peptide synthetase | +2.9 | 5.59E-04 |
| PPA1288 | surfactin synthetase subunit 1 | +3.4 | 3.03E-04 |
| PPA1289 | cysteine synthase/ornithine cyclodeaminase | +3.6 | 3.48E-04 |
| PPA1290 | cystathionine beta-synthase | +4.1 | 9.36E-05 |
| PPA1676 | ferrous iron transport protein A | +3.8 | 1.18E-04 |
| PPA1677 | ferrous iron transport protein B | +3.8 | 1.55E-04 |
| PPA1758 | outer membrane lipoprotein | +5.9 | 6.97E-05 |
| PPA1759 | ABC transporter ATP-binding protein | +5.0 | 3.37E-04 |
| PPA1760 | ABC transporter associated permease | +3.8 | 1.50E-03 |
| PPA2286 | phosphoglucomutase/phosphomannomutase | +2.9 | 6.12E-04 |
| PPA2268 | alanine dehydrogenase | +1.6 | 4.13E-02 |
| PPA0012 | L-lactate dehydrogenase | -2.3 | 3.45E-03 |
| PPA0557 | sodium- and chloride-dependent transporter | -3.8 | 1.09E-05 |
| PPA0964 | transcriptional regulator | -2.9 | 3.59E-04 |
| PPA1476 | glycine betaine transport system permease protein | -2.1 | 6.85E-03 |
| PPA1807 | hypothetical protein | -4.2 | 6.53E-06 |
| PPA2152 | putative peptide transport system secreted peptide-binding protein | -3.0 | 7.40E-04 |
| PPA2175 | rare lipoprotein A (RlpA) family protein | -4.1 | 8.71E-05 |
