# Supplementary material for: A combination of improved differential and global RNA-seq reveals pervasive transcription initiation and events in all stages of the life-cycle of functional RNAs in Propionibacterium acnes, a major contributor to wide-spread human disease
Source: BMC Genomics. 2013 Sep 14;14:620. doi: 10.1186/1471-2164-14-620 (PMC3848588; doi:10.1186/1471-2164-14-620)
Supplement: Additional file 6 — Processing of P. acnes tRNA. n.d. = not detected (scale of 0–1000 dRNA-seq reads). [file 1471-2164-14-620-S6.docx]

| **tRNA** | **Strand** | **Start** | **End** | **5' cut** | **3' cut** | **CCA** | **Position of 3' cut** |
| --- | --- | --- | --- | --- | --- | --- | --- |
| tRNA1-IleGAT | + | 22691 | 22765 | n.d. | yes | yes | CCAc↓ag |
| tRNA45-AlaTGC | - | 146178 | 146254 | weak | yes | no | C↓CCcac |
| tRNA44-SerGGA | - | 251725 | 251810 | weak | n.d. | no | n.d. |
| tRNA2-ThrCGT | + | 304047 | 304123 | yes | yes | yes | C↓CAcgt |
| tRNA3-ThrTGT | + | 488759 | 488835 | yes | yes | yes | C↓CAtac |
| tRNA43-ArgCCT | - | 539706 | 539779 | weak | yes | no | CCGc↓ag |
| tRNA4-AlaCGC | + | 547302 | 547375 | yes | weak | yes | CCAc↓tg |
| tRNA5-GlnTTG | + | 583334 | 583413 | yes | yes | no | GAGc↓tc |
| tRNA6-LeuTAA | + | 602570 | 602644 | weak | yes | no | GTAc↓ac |
| tRNA42-LeuCAA | - | 846331 | 846417 | yes | n.d. | no | n.d. |
| tRNA7-AlaGGC | + | 919756 | 919829 | yes | yes | yes | CCAc↓t↓c |
| tRNA8-AsnGTT | + | 1043182 | 1043255 | yes | yes | no | GAGctttttgaagtga↓c |
| tRNA9-MetCAT | + | 1055685 | 1055759 | weak | yes | yes | CCAc↓tt |
| tRNA41-ValGAC | - | 1167178 | 1167250 | n.d. | n.d. | yes | n.d. |
| tRNA40-CysGCA | - | 1167251 | 1167322 | yes | n.d. | no | n.d. |
| tRNA39-GlyGCC | - | 1167350 | 1167423 | yes | n.d. | no | n.d. |
| tRNA10-ValCAC | + | 1167801 | 1167876 | weak | weak | ? | ccaC↓CGaca |
| tRNA11-ProGGG | + | 1319139 | 1319213 | weak | n.d. | no | n.d. |
| tRNA12-ArgCCG | + | 1364611 | 1364683 | n.d. | yes | no | GCGc↓tc |
| tRNA13-MetCAT | + | 1385666 | 1385743 | yes | weak | yes | C↓CAgtt |
| tRNA14-ValTAC | + | 1478196 | 1478270 | yes | weak | yes | CCAc↓tt |
| tRNA38-GluCTC | - | 1487605 | 1487681 | n.d. | yes | yes | C↓CAatc |
| tRNA37-GlnCTG | - | 1487817 | 1487889 | weak | yes | no | CAGc↓gc |
| tRNA15-LeuGAG | + | 1544933 | 1545022 | weak | yes | no | C↓CCcgg |
| tRNA36-GlyTCC | - | 1704127 | 1704201 | yes | yes | yes | C↓CAttt |
| tRNA35-ProTGG | - | 1754933 | 1755009 | n.d. | yes | yes | C↓CAgct |
| tRNA34-ArgTCT | - | 1787559 | 1787635 | n.d. | weak | yes | C↓CAccg |
| tRNA16-HisGTG | + | 1789701 | 1789777 | n.d. | yes | yes | C↓CAttc |
| tRNA17-LysCTT | + | 1810145 | 1810221 | n.d. | yes | no | C↓CGaag |
| tRNA18-LeuTAG | + | 1815118 | 1815203 | n.d. | yes | no | C↓CGcag |
| tRNA33-TrpCCA | - | 2053458 | 2053534 | weak | yes | no | C↓GAgac |
| tRNA32-MetCAT | - | 2057525 | 2057600 | yes | yes | yes | C↓CAatt |
| tRNA31-ThrGGT | - | 2057665 | 2057738 | yes | yes | no | GCTct↓g |
| tRNA30-TyrGTA | - | 2057939 | 2058021 | n.d. | yes | yes | CCAc↓gc |
| tRNA29-LysTTT | - | 2124565 | 2124639 | n.d. | weak | no | GCAc↓ag |
| tRNA19-GluTTC | + | 2126450 | 2126526 | n.d. | weak | no | C↓CCtct |
| tRNA20-AspGTC | + | 2126936 | 2127010 | yes | yes | no | TCGc↓tg |
| tRNA21-PheGAA | + | 2127071 | 2127148 | n.d. | yes | no | ccacC↓CTtgc |
| tRNA22-GlyCCC | + | 2251930 | 2252004 | yes | yes | yes | C↓CAtag |
| tRNA23-LeuCAG | + | 2320456 | 2320542 | n.d. | yes | no | C↓AAcga |
| tRNA24-ProCGG | + | 2334842 | 2334919 | n.d. | yes | yes | C↓Catcc |
| tRNA28-SerCGA | - | 2362676 | 2362764 | n.d. | n.d. | yes | n.d. |
| tRNA27-ArgACG | - | 2370945 | 2371018 | n.d. | yes | no | GCGc↓ag |
| tRNA26-SerGCT | - | 2371248 | 2371338 | yes | n.d. | no | n.d. |
| tRNA25-SerTGA | - | 2378583 | 2378669 | weak | yes | yes | C↓CAcgg |
