# Supplementary material for: A combination of improved differential and global RNA-seq reveals pervasive transcription initiation and events in all stages of the life-cycle of functional RNAs in Propionibacterium acnes, a major contributor to wide-spread human disease
Source: BMC Genomics. 2013 Sep 14;14:620. doi: 10.1186/1471-2164-14-620 (PMC3848588; doi:10.1186/1471-2164-14-620)
Supplement: Additional file 7 — Sequence alignment of P. acnes promoters associated with the translational machinery. (A) shows ungapped sequences (+5 to −60) aligned to the ‘-10 box’ (consensus sequence of T.A.n.n.n.T), which was identified using MEME [69] and an initial search window of −1 to −15. (B) as (A), except gaps have been introduced 5 nt upstream of the −10 boxes to maximise alignment to the ‘-35 box’ (consensus sequence of G/A.n.T/G.T/G.n.G). Highlighting indicates nucleotides that match the consensus sequences (Figure 7). [file 1471-2164-14-620-S7.docx]

**A**

| PPA2411-ThrCGT(LE) | ------CCAGCAAAAAGCGTGTTGGTTTCGTGAAGAGGTCATCGTGCCGGTATAGTTTTCGGGGC**C**TGCCG-- |
| --- | --- |
| PPA2412-ThrTGT(LE) | ------CTCGCCTCGGCATTGAGGTGATTGACGCCGCATCGGCGATATGTGCCACAATGTTCCAC**G**TCGCC-- |
| PPA0495-rimJ(OV) | -----GCTACGCCCTAGCCGAGTCGCTGCCAGGCAGACGGCAGAACGCCCTCTAGGGTGATGCT**A**TGGCG--- |
| PPA2414-AlaCGC(OV) | -----GCCTACGCCGAATGATTCGTAGCATGGCGCGGGTTGCCCAGGTGTGGTAGCGTAGCCGC**G**TTGTT--- |
| PPA2415-GlnTTG(OV) | -----TTGAGCTTACTGAGCGTCGCTGCGTGGCCGAATTGCGATGCATGCTCTAAGGTTATGGG**G**ACTCG--- |
| PPA0535-ribso_L25P-famprot(OV) | ------GGCCACGACCTGAGCTTGCCAGGTTGTGGCCCCCGCATCGCACGAGTAGACTGCCTTGA**G**CTTGG-- |
| PPA2416-LeuTAA(OV1) | ------GACCGCTGAAGCGACGCCGTGCAGAAGTGTTGTGTGGAGGTGCCGTTATTATTGACGAG**G**TATGC-- |
| PPA2416-LeuTAA(OV2) | ----TGGAGGTGCCGTTATTATTGACGAGGTATGCCGTCGTGCGACGGCAGGTAAACTTGTCG**T**CTCGG---- |
| rRNA1-16S_p1(OV) | ------AAGATGCGGCTGTTTTGTGGGTTTGTGTTGGTGGTGGGGGTGTGTGTAGTGTCTGTTTC**T**GGCTT-- |
| rRNA1-16S_p2(OV) | ------GGTGGGGTTGCTGGGGGCATGATTTGACGTTTGTGTGATGAGTGTTTAGGCTTCTGCGG**G**TCCTG-- |
| rRNA1-16S_p3(OV) | ----GTGGTTTCGGCTGGTGTGGGCTGGGTTGTGTGATCTGGTTTGTGCTGGTATGGTTTTCC**G**GGCTG---- |
| rRNA1-16S(EN1) | ----GCTGGCTGGTTCTGGGATCGTGGTTTTGTGGTTTCGGGGTTGGTGTGGTAGGGTTGGTC**G**GGTCG---- |
| rRNA1-16S(EN2) | -----CAATAGTTTTTGATGCATCTGTTTGTTGTGGATGTGTCGGATTTGTTTATGATTCCTTT**G**TGATT--- |
| rRNA1-16S_int(EN) | ------TGACGTCAAGTCATCATGCCCCTTATGTCCAGGGCTTCACGCATGCTACAATGGCTGGT**A**CAGAG-- |
| rRNA1-23S(EN2) | -----GCGTGCGTGTGTGCGTGGTGTGGTGTTCGTGTGGTGGTTGAGAACTGTATAGTGGATGC**G**AGTAT--- |
| rRNA1-23S(EN1) | -------TGGGTTGTGGGGTATCACATGTGTGGTGGCCTGTGCGGTGCTGTGTTGCGTGCGTGTGT**G**CGTGG- |
| rRNA1-23S_int(EN1) | -------CTCCGAATGCTGGCAAGTGTAGCGTGGCAGTGAGACGGCGGGGGATAAGCTTCGTCGTC**G**AGAGG- |
| rRNA1-23S_int(EN2) | -------ATGCTCGTTACGCGCAGCAGGACGGAAAGACCCCGGGACCTTTACTATAGTTTGGTATT**G**GTGAT- |
| PPA0778-rpsA(EN) | ------TTCCCGAATGCGGACGGGGTCCGAGGGAACGGATCCAGATGTAGGCTACCTTCTTGTAT**G**ATAGA-- |
| PPA0778-rpsA(OV1) | -----GCGGACGGGGTCCGAGGGAACGGATCCAGATGTAGGCTACCTTCTTGTATGATAGACCT**C**CTATT--- |
| PPA0778-rpsA(OV2) | ------CCGTTAATGAAAAGAAGGTTGGTTGACCCTTCGTGGTTTCGGGTATTACGCTGGCTACT**G**CGTTG-- |
| PPA0827-29-rplU-rpmA(OV) | ------CAGGGTGTGGGTGGATGGTCGTTTTGACCGTCCACCGCACGAAATGTAAAATGAGCAGC**C**GGTCC-- |
| PPA2421-AlaGGC(OV) | ------TCCCGGTGCCCGTGGACGTCGATTTTGCAAAGGGGGCATGATCCGCTAAAGTTCTACGA**G**TCGCC-- |
| PPA2422-AsnGTT(OV) | -------AGGGCGTGCACATGGCGTGATTTGTTTTCGCACGAGCGATGCTGCTAATGTTTCATCTT**G**CGCTA- |
| PPA2423-MetCAT(OV) | -------ACGTCAGGGTCAGCAACGCAAGGTGCTCGCGGACGTTTTACCCGGTAAGGTAGCTACTT**G**TCTCG- |
| PPA2427-ValCAC(OV) | ---CCAGTAACGATGTTGTGGCCGGCTTGGACAAGCAGTGCTGGTTCTGGCATAGTTTCACC**A**GCAAC----- |
| PPA2428-ProGGG(OV-part of TU) | -----GGATACCAATGGTATCCGTCTCTAGGGTCCAGCCCAAATCGGTCGGCTAGGGTAGAAAA**C**CCAAG--- |
| PPA2429-ArgCCG(OV) | -----AGTGTCCATGCTCGCGATCTTGAATGGCGAGCAGCCCACCGGTGCGCTAAACTCCACAG**G**TGGTC--- |
| PPA2430-MetCAT(EN) | -----CTGTGCGCCCTACGCGAACCCCTCGAGATCAGTGTGGTAGGGCGCCCGAGAATGAGTAC**C**CACCA--- |
| PPA2430-MetCAT(OV) | ----ACCATTGGCTTTTCGGTCGGGCTTTCCCACATCTGATTTGTACTGCAACAGTGTGATCT**G**TATAG---- |
| PPA2431-ValTAC(OV) | -----GTGCGATGATTCTCCACCCGGGAAGTGTGCTTCACGTGATAAATCGGTAGAGTAGGTGC**A**CCTGG--- |
| PPA2437-LeuGAG(EN) | -----ACTTCGGCCTGCGGGCAATCTGGCCCGTCGACAAGGTTCTCGGCGGTCATACTGAACAG**A**TCTGA--- |
| PPA2437-LeuGAG(OV) | --------GGCGCTCAATGGACACTGATTTGCATTGCTGGCCGAAGGTCCATTACAGTTCTGTGACT**G**TCCGG |
| PPA2441-HisGTG(OV) | -----GCCGGCGGGCGAGAAAGTCAAAGTTGCATTTCTGTTTCAGAAACAGATACCCTTCACGG**G**TTGCC--- |
| PPA2445-LysCTT(OV) | ----TCGGTCATGAGCTTGTCACCTCGATTTTGCATCATGACCGGAGTCCGCTAGTGTTGCTT**G**TTGTC---- |
| PPA2446-LeuTAG(OV1) | -----GCAAAGAGGTTATGAAAGCCTACGGGGCCTACGGATCCAAGAACGTCTATGGTAAAGAG**A**TCGTC--- |
| PPA2446-LeuTAG(OV2) | -----TCGTCGACGTTGCTGAGGACGGTTCGTGCAAAGTCACCGAAGCCCAGTACAATGTTCGA**G**CCAAG--- |
| PPA2452-GluTTC(LE) | -----AGGCCAGCATCCGGTGGGGCAATTTCTCGCCAGTGATATGACCTGGCTATGATGGCCCA**G**TTCGA--- |
| PPA2453-AspGTC(OV) | -----TAACCCGGCATCCAGAGGTCGATTTCCCTTCAGGGTTACATCCTTGCTATATTTTTCGA**G**CTGCC--- |
| PPA2455-GlyCCC(OV) | -----CAACACTACTGAGGTGTGCGGGTTCGCACAGCTCTGACGTGGTGCGCTAAGCTATCTCA**A**GTCCT--- |
| PPA2456-LeuCAG(LE) | -----CGCAACAGAGAAGCTCCCTCAATTTCACTTCCATGCCATCTTCGTGCTACCATCTTTGG**G**TTGGG--- |
| PPA2457-ProGGG(OV) | -----TCCCCGACCCTGACAGGGCCAGCTTGGCGCTTTCGACCAAGCTGGGTTAAAGTATGCGT**C**GGCCA--- |
| PPA2409-AlaTGC(OV) | -----ACGGCATTGCGCGAGGCTTCGACTCGCCCAAACCACCGCGCATCCTCTACACTATGGAG**G**TCCAC--- |
| PPA2410-SerGGA(LE) | ----AACACACCACGACTCGCGGCCGAATTGGCGCCACGCGTCAAGTCCATGTACGCTACTTG**G**CGGAG---- |
| PPA2413-ArgCCT(LE) | ---TTTTACGACACTATGGGGTCTGAGGTTGGTCCAGGCGGCCCTGCCGGACTACTCTAGTA**A**GGCCC----- |
| PPA2420-LeuCAA(OV) | ---CCCAATCGACCCTCGTGAGCGGCGAATCGGCATCCGGGCGCAACTGTGGCACAATAGGC**A**AGCCG----- |
| PPA0898-30S-rps-S20(OV) | -----ATAACTCACAGAGGTCAACTGATTTGGGTCCAGCCGCATTGATTTGGTAGCGTGTCCTG**T**CGTGC--- |
| PPA2426-GlyGCC_PPA2425-CysGCA_PPA2424-ValGAC(OV) | -----CAAGGCGAGACGCCCACGTCGATTTGCATTGCCTGCGAAACATCCCCTAGAGTACTTTC**T**CGTCG--- |
| PPA1253-rpmE(EN) | ----CGAACCAGGATTGATGTCGTCAAGTGGACCGTGGCTGGCCGTCAGGGGTAAACTGACGC**G**CCGGT---- |
| PPA2432-GluCTC(EN) | ---AGCGCGGGAAACCAAATCCATGAAGTTGCACAGATAGCGGATGGATTGCTAGAGTTTTC**C**AGCCC----- |
| PPA2433-GlnCTG(OV) | -----GTGACAAGGAGTTTCGCCCTGATTTGCAAAGTTGGTGGATGGGCCATTAGAGTTTCTCG**T**CGTTG--- |
| rRNA2-5S(OV) | ----TACGGCTCCCAGCCCCTTCACGGGTTGCGCCGTTTAATGTATGTGTTCTACTGTATGGT**T**TTCAG---- |
| rRNA2-23S_int(EN) | -GACCCCGGGACCTTTACTATAGTTTGGTATTGGTGATTGGGACGGTTTGTGTAGGATAG**G**TGGGA------- |
| rRNA2-23S(EN) | ----GTGGTGTGGTGTTCGTGTGGTGGTTGAGAACTGTATAGTGGATGCGAGTATCTTTATTG**T**TGTAT---- |
| rRNA2-16S_p2(EN) | ----TGTTTGGGATGTTTTCTTGTCGGGTTTGACGGCGGGGGAGGGTTCCGGTATGGTTTTCC**G**GGCTG---- |
| rRNA2-16S_p1(OV) | ----AAGCGCCTCGGCTGTTTTGTGGGTTTGTGTTGGTGGTGGGGGTGTGTGTAGTGTGTGTT**G**GGCCG---- |
| PPA1413-rpmL_PPA1412-rplT(OV) | ----CCTCTCGCTACGTTTAGCCCCGATTTGTGGCGATGTCATCTCTTGCCGTACCATGAGCG**A**AGCGA---- |
| PPA1435-rplS(OV) | -----GGCGGGCACGAGTGATGGTGGATTTGATGCCAGAGCCATGGGTATGGAAAAATACCGCG**G**TGTTG--- |
| PPA1443-rpsP(OV) | ---AGTCATCAGGTGAAGTCCGTCCCGGCTCGCGTCATCTGCCTATCTGTGGCACAATGTCG**A**GAGCT----- |
| PPA1472-rpsO(LE) | ---GGCAATATCTCATCTCGCATCAGGATTCGCGCCTAATGGGCAGAGTTGATATTCTCTCC**C**AGTTG----- |
| PPA1521-rpsB(OV) | ---AAGTGGCCGGGGTCAAGGGGAAGGTTAGCTTGATTGCCTCTTGGTGTCGTAAACTAATC**G**CGCAG----- |
| PPA2438-GlyTCC(EN) | ---TCGTGGCACACCCTGTCACTGTGAATGTGACGCCGACGGTCAATCCATGTATGCTATCG**A**GGTCC----- |
| PPA2439-ProTGG(OV) | ---GAATCCCCGTCCTTGTGATGGCTCATTGGCCGAGCGGCGGGGTTTCCACTAAGCTATCG**G**TGTTC----- |
| PPA2440-ArgTCT(EN) | ----TGTTCGCTACTGGTTGGGGCCGGCTGGGCCATCAAGGCCGAACCCCGATAGTATGTCGA**A**TATGG---- |
| PPA2440-ArgTCT(OV) | ---CGGTGGGGTGGCGGGATCTGCGTGCGTCGAATCAGTCACGGGGACAGGCTAGGATGCCG**A**ACAAA----- |
| rRNA3-5S(OV) | ---ATACGGCTCCCAGCCCCTTCACGGGTTGCGCCGTTTAATGTATGTGTTCTACTGTATGG**T**TTTCA----- |
| rRNA3-23S_int2(EN) | -GACCCCGGGACCTTTACTATAGTTTGGTATTGGTGATTGGGACGGTTTGTGTAGGATAG**G**TGGGA------- |
| rRNA3-23S_int1(EN) | AAGGTTGAGGCATGATGGGGAGCCCATGGTTGTGGGTGAGTGAGTGATCCTGTACTGTC**G**AGAAA-------- |
| rRNA3-23S(EN) | ----GTGGTGTGGTGTTCGTGTGGTGGTTGAGAACTGTATAGTGGATGCGAGTATCTTTATTG**T**TGTAT---- |
| rRNA3-16S_int(EN) | ----GATGACGTCAAGTCATCATGCCCCTTATGTCCAGGGCTTCACGCATGCTACAATGGCTG**G**TACAG---- |
| rRNA3-16S_p3(EN) | ----TGTTTGGGATGTTTTCTTGTCGGGTTTGACGGCGGGGGAGGGTTCCGGTATGGTTTTCC**G**GGCTG---- |
| rRNA3-16S_p2(EN) | ----TGTTGCCGGTTGGTGGGTGTGGGTTTGTGTTGGTGGTGGGGGTGTGTGTAGTGTGTGTT**G**GGCCG---- |
| rRNA3-16S_p1(OV) | ---GACATTATCGACGTGTTGCCCGAACGTCGCGGGCTTTGGTGGTGCGAGGAATCCTCGAG**A**GAGGG----- |
| PPA1783-rimI(OV) | -----CCTGCCGGAGGACTTGGGACGGCGAGGCTGAGGTGAGGTTCGACGACTATCCTGGGTTC**T**CGTGG--- |
| PPA1783-rimI(EN) | ----ATCGTTACCTCCCACAGCTTTTGTTAGCCAGCGTGGCATCGCTGGTGGCATAGTCGCGG**A**CGCTA---- |
| PPA1802-rpsI_PPA1803-rplM(LE) | ---GCGGGAATCGACCGCCGCCCGGCGATTCGCTCTTCCCGCGGCACATGTGTAAAATACCC**A**TGTTG----- |
| PPA1852-rplN(OV) | ---CATCTTTGATGAGTCAACGCGACGACTGGCAATTTGTCGCGACGAGAGTTAAACTGCTG**A**AGTTG----- |
| PPA1852-rplN(EN2) | ----GGGGAGTAAGCGCCTTGGCTTGCGTTTCGTAGTTAGGCTAAGGCGGGCTAGCGTTGGAG**G**AGCCA---- |
| PPA1852-rplN(EN1) | ----TGTGTGTGGGGACAGGGGGAGTAAGCGCCTTGGCTTGCGTTTCGTAGTTAGGCTAAGGC**G**GGCTA---- |
| PPA1865-rpsJ(En) | ----GGCTCTCATGCGCACACTCCACCAGGCCACAGTTGTGGGGTTTTGCGATAACATTGGCG**A**CTCCT---- |
| PPA1865-rpsJ(OV) | AGTCGTTGTTGGGGTCTGACGGTCCTGATTTGCCTTGCTGGTTTGTCGGTGACACACTG**T**AGAAG-------- |
| PPA1876-rpsG_PPA1878-rpsL(LE) | -----TGCTTTGTCGTAGCGTTGTCAGGTTTGACCCTCGCAATGCAGGGGGATACCCTGTTAAG**G**CATGT--- |
| PPA2447-TrpCCA(OV) | ---TTGAAATGACTGCGGCCACCACCCGATGGATGGTTGGACGGAAGTTCTGTAGGCTTCTA**T**CGTGC----- |
| PPA2448-MetCAT_PPA2449-ThrGGT(OV) | -----GGTTGAGGAGACTCGGGTTCTGATTTGCCAACGGTCGAAAGCTCGTGTAGTTTTCTCAT**C**CGTTG--- |
| PPA2450-TyrGTA(OV) | ----CACGAGCCTCCATCCACTCGGAGAATCCCCGCTTCAGGGCAGCTCGGATACCGTTGATG**T**TGAAG---- |
| PPA2451-LysTTT(OV) | ---ATCGGCCGCCGAATACCCGGGTCGCTTCGCATGATGAAGGAAGCATCGCTACACTAGGG**G**AGCCC----- |
| PPA2458-SerCGA(EN) | ----GGCGGTACCGCTGACATACCTGAACCTTAGTCACCTGTCGATCGTCGGTACGATCTATC**A**GATGT---- |
| PPA2459-ArgACG(EN) | ---TTCCGGGGACTGAATTACGCGAATTTGCATTACGGAATTGA--AGGCTGTAATGTTTAGCA**A**CGTGC--- |
| PPA2460-SerGCT(OV) | -TGGGGCGCGTTGTCGCGGCCCGGAATTGGAGGGTTTGGGGTTG--AGAGAGTAGGCTGACA**C**GGCAC----- |
| PPA2461-SerTGA(OV) | -ATGAGCGGGTCTAGGTCCGGGACGATGAATATGCCCGATGAAT--TATGGCTAAGATGGGG**C**CACCG----- |
| PPA2227-rplI_PPA2230-rpsF(OV) | ---AATAGCCGTAGCAATGTGTTGAATTCGGAGAACGGCGGGTC--GCCCGGTATCCTTCTTCA**C**TAGCT--- |
| PPA2353-rpmH(OV) | -GGTCTGGCGGCGCAGGGTCATCAGTTTGCCGATAGCGACTCCG--ACAACGTAGAGTTGTT**A**AGTCG----- |

**B**

| PPA2411-ThrCGT(LE) | ----------CCAGCAAAAAGCGTGTTGGTTTCGTGAAGAGGTCATCGTGCCGGTATAGTTTTCGGGGC**C**TGCCG-- |
| --- | --- |
| PPA2412-ThrTGT(LE) | ---------CTCGCCTCGGCATTGAGGTGATTGACGCCGCATCGGCGAT-ATGTGCCACAATGTTCCAC**G**TCGCC-- |
| PPA0495-rimJ(OV) | ---GCTACGCCCTAGCCGAGTCGCTGCCAGGCAGACGGCAGAAC------GCCCTCTAGGGTGATGCT**A**TGGCG--- |
| PPA2414-AlaCGC(OV) | -----GCCTACGCCGAATGATTCGTAGCATGGCGCGGGTTGCCCAG----GTGTGGTAGCGTAGCCGC**G**TTGTT--- |
| PPA2415-GlnTTG(OV) | -------TTGAGCTTACTGAGCGTCGCTGCGTGGCCGAATTGCGATGC--ATGCTCTAAGGTTATGGG**G**ACTCG--- |
| PPA0535-ribso_L25P-famprot(OV) | -------GGCCACGACCTGAGCTTGCCAGGTTGTGGCCCCCGCATCG---CACGAGTAGACTGCCTTGA**G**CTTGG-- |
| PPA2416-LeuTAA(OV1) | --GACCGCTGAAGCGACGCCGTGCAGAAGTGTGTGTGGAGG--------TGCCGTTATTATTGACGAG**G**TATGC-- |
| PPA2416-LeuTAA(OV2) | ----TGGAGGTGCCGTTATTATTGACGAGGTATGCCGTCGTGCGAC----GGCAGGTAAACTTGTCG**T**CTCGG---- |
| rRNA1-16S_p1(OV) | ---AAGATGCGGCTGTTTTGTGGGTTTGTGTTGGTGGTGGGGG-------TGTGTGTAGTGTCTGTTTC**T**GGCTT-- |
| rRNA1-16S_p2(OV) | --------GGTGGGGTTGCTGGGGGCATGATTTGACGTTTGTGTGATG--AGTGTTTAGGCTTCTGCGG**G**TCCTG-- |
| rRNA1-16S_p3(OV) | -----GTGGTTTCGGCTGGTGTGGGCTGGGTTGTGTGATCTGGTTTG---TGCTGGTATGGTTTTCC**G**GGCTG---- |
| rRNA1-16S(EN1) | ------GCTGGCTGGTTCTGGGATCGTGGTTTTGTGGTTTCGGGGTTG--GTGTGGTAGGGTTGGTC**G**GGTCG---- |
| rRNA1-16S(EN2) | -----CAATAGTTTTTGATGCATCTGTTTGTTGTGGATGTGTCGGA----TTTGTTTATGATTCCTTT**G**TGATT--- |
| rRNA1-16S_int(EN) | --------TGACGTCAAGTCATCATGCCCCTTATGTCCAGGGCTTCAC--GCATGCTACAATGGCTGGT**A**CAGAG-- |
| rRNA1-23S(EN2) | -----GCGTGCGTGTGTGCGTGGTGTGGTGTTCGTGTGGTGGTTGA----GAACTGTATAGTGGATGC**G**AGTAT--- |
| rRNA1-23S(EN1) | ------TGGGTTGTGGGGTATCACATGTGTGGTGGCCTGTGCGGT-----GCTGTGTTGCGTGCGTGTGT**G**CGTGG- |
| rRNA1-23S_int(EN1) | -------CTCCGAATGCTGGCAAGTGTAGCGTGGCAGTGAGACGGC----GGGGGATAAGCTTCGTCGTC**G**AGAGG- |
| rRNA1-23S_int(EN2) | -------ATGCTCGTTACGCGCAGCAGGACGGAAAGACCCCGGGAC----CTTTACTATAGTTTGGTATT**G**GTGAT- |
| PPA0778-rpsA(EN) | ------TTCCCGAATGCGGACGGGGTCCGAGGGAACGGATCCAGAT----GTAGGCTACCTTCTTGTAT**G**ATAGA-- |
| PPA0778-rpsA(OV1) | ------GCGGACGGGGTCCGAGGGAACGGATCCAGATGTAGGCTACC---TTCTTGTATGATAGACCT**C**CTATT--- |
| PPA0778-rpsA(OV2) | --------CCGTTAATGAAAAGAAGGTTGGTTGACCCTTCGTGGTTTC--GGGTATTACGCTGGCTACT**G**CGTTG-- |
| PPA0827-29-rplU-rpmA(OV) | --------CAGGGTGTGGGTGGATGGTCGTTTTGACCGTCCACCGCAC--GAAATGTAAAATGAGCAGC**C**GGTCC-- |
| PPA2421-AlaGGC(OV) | -------TCCCGGTGCCCGTGGACGTCGATTTTGCAAAGGGGGCATG---ATCCGCTAAAGTTCTACGA**G**TCGCC-- |
| PPA2422-AsnGTT(OV) | -----AGGGCGTGCACATGGCGTGATTTGTTTTCGCACGAGCGA------TGCTGCTAATGTTTCATCTT**G**CGCTA- |
| PPA2423-MetCAT(OV) | -------ACGTCAGGGTCAGCAACGCAAGGTGCTCGCGGACGTTTT----ACCCGGTAAGGTAGCTACTT**G**TCTCG- |
| PPA2427-ValCAC(OV) | -------CCAGTAACGATGTTGTGGCCGGCTTGGACAAGCAGTGCTGGTTCTGGCATAGTTTCACC**A**GCAAC----- |
| PPA2428-ProGGG(OV-part of TU) | ----GGATACCAATGGTATCCGTCTCTAGGGTCCAGCCCAAATCG-----GTCGGCTAGGGTAGAAAA**C**CCAAG--- |
| PPA2429-ArgCCG(OV) | -------AGTGTCCATGCTCGCGATCTTGAATGGCGAGCAGCCCACCG--GTGCGCTAAACTCCACAG**G**TGGTC--- |
| PPA2430-MetCAT(EN) | --CTGTGCGCCCTACGCGAACCCCTCGAGATCAGTGTGGTAGG-------GCGCCCGAGAATGAGTAC**C**CACCA--- |
| PPA2430-MetCAT(OV) | -------ACCATTGGCTTTTCGGTCGGGCTTTCCCACATCTGATTTGTA-CTGCAACAGTGTGATCT**G**TATAG---- |
| PPA2431-ValTAC(OV) | -----GTGCGATGATTCTCCACCCGGGAAGTGTGCTTCACGTGATA----AATCGGTAGAGTAGGTGC**A**CCTGG--- |
| PPA2437-LeuGAG(EN) | ----ACTTCGGCCTGCGGGCAATCTGGCCCGTCGACAAGGTTCTC-----GGCGGTCATACTGAACAG**A**TCTGA--- |
| PPA2437-LeuGAG(OV) | -----------GGCGCTCAATGGACACTGATTTGCATTGCTGGCCGAAG-GTCCATTACAGTTCTGTGACT**G**TCCGG |
| PPA2441-HisGTG(OV) | -------GCCGGCGGGCGAGAAAGTCAAAGTTGCATTTCTGTTTCAGA--AACAGATACCCTTCACGG**G**TTGCC--- |
| PPA2445-LysCTT(OV) | -----TCGGTCATGAGCTTGTCACCTCGATTTTGCATCATGACCGGA---GTCCGCTAGTGTTGCTT**G**TTGTC---- |
| PPA2446-LeuTAG(OV1) | -------GCAAAGAGGTTATGAAAGCCTACGGGGCCTACGGATCCAAG--AACGTCTATGGTAAAGAG**A**TCGTC--- |
| PPA2446-LeuTAG(OV2) | --------TCGTCGACGTTGCTGAGGACGGTTCGTGCAAAGTCACCGAA-GCCCAGTACAATGTTCGA**G**CCAAG--- |
| PPA2452-GluTTC(LE) | -------AGGCCAGCATCCGGTGGGGCAATTTCTCGCCAGTGATATGA--CCTGGCTATGATGGCCCA**G**TTCGA--- |
| PPA2453-AspGTC(OV) | --------TAACCCGGCATCCAGAGGTCGATTTCCCTTCAGGGTTACAT-CCTTGCTATATTTTTCGA**G**CTGCC--- |
| PPA2455-GlyCCC(OV) | --------CAACACTACTGAGGTGTGCGGGTTCGCACAGCTCTGACGTG-GTGCGCTAAGCTATCTCA**A**GTCCT--- |
| PPA2456-LeuCAG(LE) | --------CGCAACAGAGAAGCTCCCTCAATTTCACTTCCATGCCATCT-TCGTGCTACCATCTTTGG**G**TTGGG--- |
| PPA2457-ProGGG(OV) | -------TCCCCGACCCTGACAGGGCCAGCTTGGCGCTTTCGACCAAG--CTGGGTTAAAGTATGCGT**C**GGCCA--- |
| PPA2409-AlaTGC(OV) | --------ACGGCATTGCGCGAGGCTTCGACTCGCCCAAACCACCGCGC-ATCCTCTACACTATGGAG**G**TCCAC--- |
| PPA2410-SerGGA(LE) | ------AACACACCACGACTCGCGGCCGAATTGGCGCCACGCGTCAAG--TCCATGTACGCTACTTG**G**CGGAG---- |
| PPA2413-ArgCCT(LE) | -----TTTTACGACACTATGGGGTCTGAGGTTGGTCCAGGCGGCCCTG--CCGGACTACTCTAGTA**A**GGCCC----- |
| PPA2420-LeuCAA(OV) | -----CCCAATCGACCCTCGTGAGCGGCGAATCGGCATCCGGGCGCAA--CTGTGGCACAATAGGC**A**AGCCG----- |
| PPA0898-30S-rps-S20(OV) | --------ATAACTCACAGAGGTCAACTGATTTGGGTCCAGCCGCATTG-ATTTGGTAGCGTGTCCTG**T**CGTGC--- |
| PPA2426-GlyGCC_PPA2425-CysGCA_PPA2424-ValGAC(OV) | --------CAAGGCGAGACGCCCACGTCGATTTGCATTGCCTGCGAAAC-ATCCCCTAGAGTACTTTC**T**CGTCG--- |
| PPA1253-rpmE(EN) | -------CGAACCAGGATTGATGTCGTCAAGTGGACCGTGGCTGGCCGT-CAGGGGTAAACTGACGC**G**CCGGT---- |
| PPA2432-GluCTC(EN) | ------AGCGCGGGAAACCAAATCCATGAAGTTGCACAGATAGCGGATG-GATTGCTAGAGTTTTC**C**AGCCC----- |
| PPA2433-GlnCTG(OV) | --------GTGACAAGGAGTTTCGCCCTGATTTGCAAAGTTGGTGGATG-GGCCATTAGAGTTTCTCG**T**CGTTG--- |
| rRNA2-5S(OV) | ------TACGGCTCCCAGCCCCTTCACGGGTTGCGCCGTTTAATGTAT--GTGTTCTACTGTATGGT**T**TTCAG---- |
| rRNA2-23S_int(EN) | -GACCCCGGGACCTTTACTATAGTTTGGTATTGGTGATTGGGACGG----TTTGTGTAGGATAG**G**TGGGA------- |
| rRNA2-23S(EN) | -------GTGGTGTGGTGTTCGTGTGGTGGTTGAGAACTGTATAGTGGA-TGCGAGTATCTTTATTG**T**TGTAT---- |
| rRNA2-16S_p2(EN) | ------TGTTTGGGATGTTTTCTTGTCGGGTTTGACGGCGGGGGAGGG--TTCCGGTATGGTTTTCC**G**GGCTG---- |
| rRNA2-16S_p1(OV) | -------AAGCGCCTCGGCTGTTTTGTGGGTTTGTGTTGGTGGTGGGGG-TGTGTGTAGTGTGTGTT**G**GGCCG---- |
| PPA1413-rpmL_PPA1412-rplT(OV) | -------CCTCTCGCTACGTTTAGCCCCGATTTGTGGCGATGTCATCTC-TTGCCGTACCATGAGCG**A**AGCGA---- |
| PPA1435-rplS(OV) | --------GGCGGGCACGAGTGATGGTGGATTTGATGCCAGAGCCATGG-GTATGGAAAAATACCGCG**G**TGTTG--- |
| PPA1443-rpsP(OV) | -----AGTCATCAGGTGAAGTCCGTCCCGGCTCGCGTCATCTGCCTAT--CTGTGGCACAATGTCG**A**GAGCT----- |
| PPA1472-rpsO(LE) | -----GGCAATATCTCATCTCGCATCAGGATTCGCGCCTAATGGGCAG--AGTTGATATTCTCTCC**C**AGTTG----- |
| PPA1521-rpsB(OV) | -AAGTGGCCGGGGTCAAGGGGAAGGTTAGCTTGATTGCCTCTTG------GTGTCGTAAACTAATC**G**CGCAG----- |
| PPA2438-GlyTCC(EN) | -----TCGTGGCACACCCTGTCACTGTGAATGTGACGCCGACGGTCAA--TCCATGTATGCTATCG**A**GGTCC----- |
| PPA2439-ProTGG(OV) | -----GAATCCCCGTCCTTGTGATGGCTCATTGGCCGAGCGGCGGGGT--TTCCACTAAGCTATCG**G**TGTTC----- |
| PPA2440-ArgTCT(EN) | ------TGTTCGCTACTGGTTGGGGCCGGCTGGGCCATCAAGGCCGAA--CCCCGATAGTATGTCGA**A**TATGG---- |
| PPA2440-ArgTCT(OV) | -----CGGTGGGGTGGCGGGATCTGCGTGCGTCGAATCAGTCACGGGG--ACAGGCTAGGATGCCG**A**ACAAA----- |
| rRNA3-5S(OV) | ------ATACGGCTCCCAGCCCCTTCACGGGTTGCGCCGTTTAATGTAT-GTGTTCTACTGTATGG**T**TTTCA----- |
| rRNA3-23S_int2(EN) | -GACCCCGGGACCTTTACTATAGTTTGGTATTGGTGATTGGGACGG----TTTGTGTAGGATAG**G**TGGGA------- |
| rRNA3-23S_int1(EN) | AAGGTTGAGGCATGATGGGGAGCCCATGGTTGTGGGTGAGTGAGTG----ATCCTGTACTGTC**G**AGAAA-------- |
| rRNA3-23S(EN) | ------GTGGTGTGGTGTTCGTGTGGTGGTTGAGAACTGTATAGTGGA--TGCGAGTATCTTTATTG**T**TGTAT---- |
| rRNA3-16S_int(EN) | ------GATGACGTCAAGTCATCATGCCCCTTATGTCCAGGGCTTCAC--GCATGCTACAATGGCTG**G**TACAG---- |
| rRNA3-16S_p3(EN) | ------TGTTTGGGATGTTTTCTTGTCGGGTTTGACGGCGGGGGAGGG--TTCCGGTATGGTTTTCC**G**GGCTG---- |
| rRNA3-16S_p2(EN) | -------TGTTGCCGGTTGGTGGGTGTGGGTTTGTGTTGGTGGTGGGGG-TGTGTGTAGTGTGTGTT**G**GGCCG---- |
| rRNA3-16S_p1(OV) | -----GACATTATCGACGTGTTGCCCGAACGTCGCGGGCTTTGGTGGT--GCGAGGAATCCTCGAG**A**GAGGG----- |
| PPA1783-rimI(OV) | -----CCTGCCGGAGGACTTGGGACGGCGAGGCTGAGGTGAGGTTC----GACGACTATCCTGGGTTC**T**CGTGG--- |
| PPA1783-rimI(EN) | -------ATCGTTACCTCCCACAGCTTTTGTTAGCCAGCGTGGCATCGC-TGGTGGCATAGTCGCGG**A**CGCTA---- |
| PPA1802-rpsI_PPA1803-rplM(LE) | -----GCGGGAATCGACCGCCGCCCGGCGATTCGCTCTTCCCGCGGCA--CATGTGTAAAATACCC**A**TGTTG----- |
| PPA1852-rplN(OV) | -----CATCTTTGATGAGTCAACGCGACGACTGGCAATTTGTCGCGAC--GAGAGTTAAACTGCTG**A**AGTTG----- |
| PPA1852-rplN(EN2) | -----GGGGAGTAAGCGCCTTGGCTTGCGTTTCGTAGTTAGGCTAAG---GCGGGCTAGCGTTGGAG**G**AGCCA---- |
| PPA1852-rplN(EN1) | -TGTGTGTGGGGACAGGGGGAGTAAGCGCCTTGGCTTGCGTTT-------CGTAGTTAGGCTAAGGC**G**GGCTA---- |
| PPA1865-rpsJ(En) | --------GGCTCTCATGCGCACACTCCACCAGGCCACAGTTGTGGGGTTTTGCGATAACATTGGCG**A**CTCCT---- |
| PPA1865-rpsJ(OV) | --AGTCGTTGTTGGGGTCTGACGGTCCTGATTTGCCTTGCTGGTTTGT--CGGTGACACACTG**T**AGAAG-------- |
| PPA1876-rpsG_PPA1878-rpsL(LE) | -------TGCTTTGTCGTAGCGTTGTCAGGTTTGACCCTCGCAATGCA--GGGGGATACCCTGTTAAG**G**CATGT--- |
| PPA2447-TrpCCA(OV) | -TTGAAATGACTGCGGCCACCACCCGATGGATGGTTGGACGGAA------GTTCTGTAGGCTTCTA**T**CGTGC----- |
| PPA2448-MetCAT_PPA2449-ThrGGT(OV) | -------GGTTGAGGAGACTCGGGTTCTGATTTGCCAACGGTCGAAAG--CTCGTGTAGTTTTCTCAT**C**CGTTG--- |
| PPA2450-TyrGTA(OV) | ------CACGAGCCTCCATCCACTCGGAGAATCCCCGCTTCAGGGCAG--CTCGGATACCGTTGATG**T**TGAAG---- |
| PPA2451-LysTTT(OV) | -----ATCGGCCGCCGAATACCCGGGTCGCTTCGCATGATGAAGGAAG--CATCGCTACACTAGGG**G**AGCCC----- |
| PPA2458-SerCGA(EN) | ----GGCGGTACCGCTGACATACCTGAACCTTAGTCACCTGTCGAT----CGTCGGTACGATCTATC**A**GATGT---- |
| PPA2459-ArgACG(EN) | -------TTCCGGGGACTGAATTACGCGAATTTGCATTACGGAATTGA--AGGCTGTAATGTTTAGCA**A**CGTGC--- |
| PPA2460-SerGCT(OV) | -----TGGGGCGCGTTGTCGCGGCCCGGAATTGGAGGGTTTGGGGTTG--AGAGAGTAGGCTGACA**C**GGCAC----- |
| PPA2461-SerTGA(OV) | -----ATGAGCGGGTCTAGGTCCGGGACGATGAATATGCCCGATGAAT--TATGGCTAAGATGGGG**C**CACCG----- |
| PPA2227-rplI_PPA2230-rpsF(OV) | -------AATAGCCGTAGCAATGTGTTGAATTCGGAGAACGGCGGGTC--GCCCGGTATCCTTCTTCA**C**TAGCT--- |
| PPA2353-rpmH(OV) | -----GGTCTGGCGGCGCAGGGTCATCAGTTTGCCGATAGCGACTCCG--ACAACGTAGAGTTGTT**A**AGTCG----- |
