# Supplementary material for: A combination of improved differential and global RNA-seq reveals pervasive transcription initiation and events in all stages of the life-cycle of functional RNAs in Propionibacterium acnes, a major contributor to wide-spread human disease
Source: BMC Genomics. 2013 Sep 14;14:620. doi: 10.1186/1471-2164-14-620 (PMC3848588; doi:10.1186/1471-2164-14-620)
Supplement: Additional file 8 — List of annotated and possible sRNAs in P. acnes. [file 1471-2164-14-620-S8.docx]

| **Position** | **Left** | **Right** | **Size (nt)** | **Strand** |  |
| --- | --- | --- | --- | --- | --- |
| ***Abundant 5'UTRs / riboswitches*** | | | | | ***Corresponding gene*** |
| 16650 | 16650 | 16821 | 171 | + | PPA0014 |
| 95813 | 95813 | 95860 | 47 | + | PPA0085 |
| 133014 | 133014 | 133102 | 88 | + | PPA0110 (TPP)* |
| 186522 | 186522 | 186663 | 141 | + | PPA0156 (TPP)* |
| 199776 | 199776 | 199912 | 136 | + | PPA0166 |
| 326577 | 326577 | 326684 | 107 | + | PPA0278 |
| 470748 | 470748 | 470959 | 211 | + | PPA0425 (Cobalamin) |
| 476077 | 476077 | 476239 | 162 | + | PPA0430 |
| 656880 | 656880 | 657103 | 223 | + | PPA0595 (Cobalamin) |
| 720186 | 720186 | 720338 | 152 | + | PPA0656 (Cobalamin) |
| 962714 | 962714 | 962863 | 149 | + | PPA0885 (TPP) |
| 1129093 | 1129093 | 1129305 | 212 | + | PPA1037 |
| 1933084 | 1933084 | 1933156 | 72 | + | PPA1770 |
| 2294577 | 2294577 | 2294822 | 245 | + | PPA2121 (Cobalamin) |
| 387710 | 387465 | 387710 | 245 | - | PPA0336 (Cobalamin) |
| 574371 | 574260 | 574371 | 111 | - | PPA0520 (TPP)* |
| 1058658 | 1058593 | 1058658 | 65 | - | PPA2378 |
| 1719380 | 1719299 | 1719382 | 83 | - | PPA1592 |
| 1913792 | 1913638 | 1913792 | 154 | - | PPA1752 (FMN) |
| 1996134 | 1996040 | 1996134 | 94 | - | PPA1826 |
| 2317705 | 2317484 | 2317705 | 221 | - | PPA2137 (Cobalamin) |
|  | | | | | |
| ***Possible asRNA regulators of translation initiation*** | | | | | ***Possible target*** |
| 523981 | 523981 | 524271 | 290 | + | PPA0473 (RBS) |
| 690031 | 690031 | 690381 | 350 | + | PPA0624 (RBS) |
| 885274 | 885274 | 885413 | 139 | + | PPA0805 (RBS) |
| 1731448 | 1731448 | 1731599 | 151 | + | PPA1606 (RBS) |
| 2386888 | 2386888 | 2387160 | 272 | + | PPA2203 (RBS) |
| 662829 | 662711 | 662829 | 118 | - | PPA0598 (RBS) |
| 981518 | 981410 | 981518 | 108 | - | PPA0901 (RBS) |
| 1163170 | 1162920 | 1163170 | 250 | - | PPA1068 (RBS) |
|  | | | | | |
| 419298 | 419298 | 419571 | 273 | + | PPA0371 (int RBS) |
| 601083 | 600986 | 601083 | 97 | - | PPA0547 (int RBS) |
| 1062051 | 1061768 | 1062051 | 283 | - | PPA0977 (int RBS) |
|  | | | | | |
| ***Possible asRNA regulators*** | | | | | ***Possible target*** |
| 54282 | 54282 | 54374 | 92 | + | PPA0054 (ORF) |
| 107414 | 107414 | 107526 | 112 | + | PPA0093 (ORF) |
| 226602 | 226602 | 226725 | 123 | + | PPA0185 (ORF) |
| 262666 | 262666 | 262838 | 172 | + | PPA0210 (ORF) |
| 350628 | 350628 | 350805 | 177 | + | PPA0300 (ORF) |
| 525729 | 525729 | 525842 | 113 | + | PPA0474 (ORF) |
| 776546 | 776546 | 776703 | 157 | + | PPA0704 (ORF) |
| 1220499 | 1220499 | 1220700 | 201 | + | PPA1121 (ORF) |
| 1543535 | 1543535 | 1543741 | 206 | + | PPA1424 (ORF) |
| 1573376 | 1573376 | 1573528 | 152 | + | PPA1457 (ORF) |
| 1902900 | 1902900 | 1903033 | 133 | + | PPA1743 (ORF) |
| 48200 | 47996 | 48200 | 204 | - | PPA0049 (ORF) |
| 429137 | 428647 | 429137 | 490 | - | PPA0381 (ORF) |
| 515180 | 515006 | 515180 | 174 | - | PPA0467 (ORF) |
| 578506 | 578420 | 578506 | 86 | - | PPA0524 (ORF) |
| 586175 | 585963 | 586175 | 212 | - | PPA0532 (ORF) |
| 1202004 | 1201732 | 1202004 | 272 | - | PPA1108 (ORF) |
| 1251316 | 1250985 | 1251316 | 331 | - | PPA1150 (ORF) |
| 1625671 | 1625525 | 1625671 | 146 | - | PPA1502 (ORF) |
| 1747014 | 1746890 | 1747014 | 124 | - | PPA1615 (ORF) |
| 2067110 | 2066844 | 2067110 | 266 | - | PPA1906 (ORF) |
| 2154776 | 2154511 | 2154776 | 265 | - | PPA1983 (ORF) |
| 2286407 | 2286210 | 2286407 | 197 | - | PPA2112 (ORF) |
|  | | | | | |
| 234105 | 234105 | 234245 | 140 | + | PPA0192 (3' UTR) |
| 647993 | 647847 | 647993 | 146 | - | PPA0585 (3' UTR) |
| 788348 | 788348 | 788480 | 132 | + | PPA0717 (3' UTR) |
| 947536 | 947536 | 947640 | 104 | + | PPA0869 (3' UTR) |
| 1642082 | 1642082 | 1642227 | 145 | + | PPA1520 (3' UTR) |
| 2048808 | 2048808 | 2048966 | 158 | + | PPA1885 (3' UTR) |
| 2354014 | 2354014 | 2354331 | 317 | + | PPA2172 (3' UTR) |
|  | | | | | |
| ***Other types of sRNA*** | | | | | ***Comments*** |
| 148251 | 148251 | 148400 | 149 | + |  |
| 235787 | 235787 | 235992 | 205 | + |  |
| 252385 | 252385 | 252476 | 91 | + | SRP RNA |
| 718285 | 718285 | 718670 | 385 | + | RNase P |
| 719025 | 719025 | 719086 | 61 | + |  |
| 764909 | 764909 | 765035 | 126 | + |  |
| 931846 | 931846 | 932107 | 261 | + |  |
| 945288 | 945288 | 945746 | 458 | + |  |
| 1248645 | 1248645 | 1248749 | 104 | + |  |
| 1372793 | 1372793 | 1373002 | 209 | + |  |
| 2050281 | 2050281 | 2050431 | 150 | + |  |
| 447936 | 447859 | 447936 | 77 | - |  |
| 1377440 | 1377320 | 1377440 | 120 | - |  |
| 1467934 | 1467548 | 1467934 | 386 | - | tmRNA |
| 1815486 | 1815367 | 1815486 | 119 | - |  |
| 2038209 | 2038209 | 2038218 | 9 | - |  |
| 2126592 | 2126514 | 2126592 | 78 | - |  |
| 2321632 | 2321460 | 2321632 | 172 | - |  |
| 2338912 | 2338912 | 2339128 | 216 | + | within coding region |
| 2551421 | 2551396 | 2551421 | 25 | + | within coding region |
