# Supplementary material for: A combination of improved differential and global RNA-seq reveals pervasive transcription initiation and events in all stages of the life-cycle of functional RNAs in Propionibacterium acnes, a major contributor to wide-spread human disease
Source: BMC Genomics. 2013 Sep 14;14:620. doi: 10.1186/1471-2164-14-620 (PMC3848588; doi:10.1186/1471-2164-14-620)
Supplement: Additional file 9 — List of possible leaderless mRNAs. [file 1471-2164-14-620-S9.docx]

| **TSS** | **Gene** | **Leader (nt)** | **Codon** |
| --- | --- | --- | --- |
| 85492 | PPA0079 | 1 | ATG |
| 219005-7 | PPA0180 | 0 | ATG |
| 264693 | PPA0213 | 8 | ATG |
| 275563 | PPA0220 | 0 | GTG |
| 301857 | PPA0248 | 18 | ATG |
| 310295 | PPA0257 | 20 | GTG |
| 312708 | PPA0259 | 0 | GTG |
| 332153 | PPA0282 | 0 | ATG |
| 357872 | PPA0307 | 15 | TTG |
| 383235 | PPA0333 | 0 | ATG |
| 396972 | PPA0345 | 9 | ATG |
| 453574 | PPA0408 | 0 | GTG |
| 544192 | PPA0494 | 0 | ATG |
| 565188 | PPA0513 | 0 | GTG |
| 581080 | PPA0527 | 0 | ATG |
| 619022 | PPA0559 | 6 | ATG |
| 620292 | PPA0560 | 0 | ATG |
| 632907 | PPA0572 | 0 | ATG |
| 636616 | PPA0575 | 0 | GTG |
| 649446 | PPA0588 | 0 | ATG |
| 651011 | PPA0590 | 0 | ATG |
| 701664 | PPA0636 | 0 | ATG |
| 707657 | PPA0643 | 0 | ATG |
| 712934 | PPA0648 | 20 | ATG |
| 719343 | PPA0653 | 0 | ATG |
| 720186 | PPA0656 | 12 | GTG |
| 744036 | PPA0676 | 0 | ATG |
| 795728-30 | PPA0724 | 13 | ATG |
| 802023-5 | PPA0730 | 1 | ATG |
| 843812 | PPA0769 | 0 | GTG |
| 977946-8 | PPA0897 | 2 | GTG |
| 1007244 | PPA0924 | 0 | ATG |
| 1025484 | PPA0944 | 0 | ATG |
| 1042580 | PPA0959 | 0 | ATG |
| 1094775 | PPA1011 | 15 | TTG |
| 1101306 | PPA1017 | 3 | ATG |
| 1131717 | PPA1040 | 0 | ATG |
| 1152133-5 | PPA1060 | 2 | ATG |
| 1278152 | PPA1173 | 0 | ATG |
| 1332639-41 | PPA1225 | 2 | GTG |
| 1335132 | PPA1227 | 0 | ATG |
| 1439333 | PPA1323 | 0 | ATG |
| 1486763 | PPA1364 | 0 | ATG |
| 1580884 | PPA1465 | 0 | ATG |
| 1614866 | PPA1496 | 0 | ATG |
| 1677186 | PPA1557 | 0 | GTG |
| 1687656 | PPA1568 | 0 | ATG |
| 1789002 | PPA1642 | 10 | ATG |
| 1851527 | PPA1699 | 0 | ATG |
| 1875582 | PPA1722 | 0 | ATG |
| 1924939 | PPA1762 | 0 | ATG |
| 2070668 | PPA1909 | 0 | ATG |
| 2103935 | PPA1943 | 1 | GTG |
| 2113770 | PPA1953 | 0 | ATG |
| 2124998 | PPA1962 | 20 | TTG |
| 2200062-4 | PPA2027 | 2 | GTG |
| 2262758 | PPA2088 | 13 | GTG |
| 2270276 | PPA2095 | 12 | GTG |
| 2285027 | PPA2111 | 0 | ATG |
| 2343229 | PPA2163 | 0 | ATG |
| 2359454 | PPA2177 | 0 | GTG |
| 2365576 | PPA2184 | 0 | GTG |
| 2367840 | PPA2187 | 1 | GTG |
| 2380556 | PPA2200 | 0 | ATG |
| 2383635 | PPA2202 | 0 | GTG |
| 2388012 | PPA2205 | 0 | ATG |
| 2420039 | PPA2236 | 0 | ATG |
| 2430005 | PPA2246 | 0 | ATG |
| 2441486 | PPA2257 | 0 | ATG |
| 2453150 | PPA2267 | 18 | ATG |
| 2484814 | PPA2289 | 0 | ATG |
| 2486987 | PPA2292 | 0 | ATG |
| 157289 | PPA0130 | 1 | ATG |
| 160753 | PPA0133 | 1 | GTG |
| 213202 | PPA0176 | 1 | ATG |
| 310324 | PPA0256 | 1 | GTG |
| 366974 | PPA0314 | 1 | ATG |
| 380907 | PPA0330 | 1 | ATG |
| 398318 | PPA0346 | 1 | ATG |
| 412518 | PPA0364 | 1 | ATG |
| 480388 | PPA0436 | 1 | ATG |
| 527105 | PPA0475 | 1 | ATG |
| 578338 | PPA0523 | 1 | GTG |
| 583253 | PPA0529 | 1 | ATG |
| 621885-7 | PPA0561 | 1 | ATG |
| 625488 | PPA0564 | 1 | ATG |
| 629901 | PPA0569 | 1 | ATG |
| 635103 | PPA0573 | 1 | ATG |
| 689518 | PPA0623 | 1 | ATG |
| 696913 | PPA0630 | 1 | ATG |
| 706656 | PPA0641 | 1 | GTG |
| 712849 | PPA0647 | 1 | GTG |
| 727624-6 | PPA0661 | 1 | GTG |
| 728897 | PPA0663 | 1 | ATG |
| 735523 | PPA0667 | 3 | GTG |
| 759849 | PPA0689 | 1 | GTG |
| 781355 | PPA0708 | 1 | GTG |
| 817762 | PPA0744 | 1 | ATG |
| 924594 | PPA0845 | 1 | ATG |
| 1069245 | PPA0986 | 1 | ATG |
| 1141827 | PPA1046 | 1 | ATG |
| 1146258 | PPA1052 | 1 | ATG |
| 1196203 | PPA1104 | 1 | ATG |
| 1198468 | PPA1105 | 1 | ATG |
| 1239376 | PPA1139 | 1 | ATG |
| 1352430 | PPA1246 | 4 | ATG |
| 1364491 | PPA1259 | 1 | ATG |
| 1468550 | PPA1344 | 1 | ATG |
| 1478097 | PPA1354 | 1 | GTG |
| 1489728 | PPA1366 | 1 | ATG |
| 1492227-9 | PPA1368 | 1 | ATG |
| 1497174 | PPA1376 | 7 | GTG |
| 1507765 | PPA1387 | 8 | TTG |
| 1511081-3 | PPA1390 | 1 | GTG |
| 1540932 | PPA1421 | 1 | ATG |
| 1564809 | PPA1447 | 1 | GTG |
| 1633757 | PPA1510 | 1 | GTG |
| 1652327 | PPA1530 | 1 | ATG |
| 1654714 | PPA1533 | 1 | ATG |
| 1669229 | PPA1550 | 1 | GTG |
| 1762569 | PPA1624 | 1 | ATG |
| 1766231 | PPA1626 | 1 | ATG |
| 1787483 | PPA1640 | 1 | ATG |
| 1833078 | PPA1680 | 1 | ATG |
| 1849598 | PPA1696 | 1 | ATG |
| 1851422 | PPA1698 | 1 | ATG |
| 1860031 | PPA1705 | 1 | GTG |
| 1864065 | PPA1710 | 1 | ATG |
| 1941266 | PPA1776 | 1 | GTG |
| 1948769 | PPA1785 | 1 | GTG |
| 1966431 | PPA1801 | 1 | ATG |
| 2057156 | PPA1896 | 1 | ATG |
| 2060023 | PPA1899 | 10 | GTG |
| 2062981 | PPA1903 | 1 | GTG |
| 2062989 | PPA1903 | 8 | GTG |
| 2112549 | PPA1951 | 0 | GTG |
| 2134455 | PPA1970 | 1 | GTG |
| 2145840 | PPA1977 | 1 | GTG |
| 2160342 | PPA1988 | 1 | ATG |
| 2174714 | PPA2002 | 1 | ATG |
| 2181571 | PPA2008 | 1 | ATG |
| 2187838 | PPA2015 | 1 | ATG |
| 2289917 | PPA2115 | 1 | ATG |
| 2305442 | PPA2128 | 1 | ATG |
| 2311086 | PPA2133 | 1 | ATG |
| 2410592 | PPA2225 | 1 | ATG |
| 2419928 | PPA2235 | 1 | ATG |
| 2436830 | PPA2251 | 0 | ATG |
| 2493898 | PPA2297 | 1 | ATG |
