# Supplementary material for: A combination of improved differential and global RNA-seq reveals pervasive transcription initiation and events in all stages of the life-cycle of functional RNAs in Propionibacterium acnes, a major contributor to wide-spread human disease
Source: BMC Genomics. 2013 Sep 14;14:620. doi: 10.1186/1471-2164-14-620 (PMC3848588; doi:10.1186/1471-2164-14-620)
Supplement: Additional file 10 — List of genes requiring reannotation. [file 1471-2164-14-620-S10.docx]

| **TSS** | **Gene** | **Strand** | **Annotated start** | **Suggested start** | **Stop position** |
| --- | --- | --- | --- | --- | --- |
| 167222 | PPA0139 | + | 167045 | 167264 | 168424 |
| 407190 | PPA0358 | + | 407115 | 407190 | 407720 |
| 544192 | PPA0494 | + | 544192 | 544279 | 545499 |
| 847733 | PPA0774 | + | 847616 | 847742 | 850411 |
| 859207 | PPA0781 | + | 858938 | 859207 | 859996 |
| 962714 | PPA0885 | + | 962597 | 962816 | 963625 |
| 1029267 | PPA0949 | + | 1029249 | 1029396 | 1030769 |
| 1109579 | PPA1024 | + | 1109551 | 1109580 | 1110024 |
| 1193501 | PPA1102 | + | 1193412 | 1193502 | 1194209 |
| 1198600 | PPA1106 | + | 1198591 | 1198600 | 1199532 |
| 1374116 | PPA1267 | + | 1373997 | 1374191 | 1374818 |
| 1536907 | PPA1416 | + | 1536889 | 1536907 | 1537506 |
| 1941314 | PPA1777 | + | 1941294 | 1941314 | 1941941 |
| 1972911 | PPA1808 | + | 1972797 | 1972911 | 1973741 |
| 2108923 | PPA1947 | + | 2108896 | 2108923 | 2109924 |
| 2113770 | PPA1953 | + | 2113770 | 2113881 | 2115272 |
| 2156557 | PPA1984 | + | 2156438 | 2156555 | 2156995 |
| 2168990 | PPA1996 | + | 2168978 | 2169050 | 2169712 |
| 2335106 | PPA2151 | + | 2335002 | 2335131 | 2335670 |
| 282252 | PPA0228 | - | 282452 | 282251 | 281493 |
| 396911 | PPA0344 | - | 396922 | 396853 | 396209 |
| 432713-5 | PPA0384 | - | 432754 | 432715 | 431282 |
| 460965 | PPA0417 | - | 461102 | 460967 | 460395 |
| 969725-30 | PPA0892 | - | 969946 | 969725 | 968888 |
| 1016471 | PPA0935 | - | 1016485 | 1016469 | 1016060 |
| 1273602 | PPA1171 | - | 1273628 | 1273601 | 1273038 |
| 1335012 | PPA1226 | - | 1335102 | 1334982 | 1333456 |
| 1417207 | PPA1310 | - | 1417245 | 1417215 | 1416067 |
| 1422393 | PPA1314 | - | 1422428 | 1422392 | 1420308 |
| 1593674 | PPA1474 | - | 1593919 | 1593674 | 1592777 |
| 1865903 | PPA1712 | - | 1866004 | 1865903 | 1865099 |
| 1927522 | PPA1764 | - | 1927560 | 1927521 | 1925968 |
| 1960826 | PPA1794 | - | 1960891 | 1960826 | 1958978 |
| 2075431 | PPA1913 | - | 2075493 | 2075431 | 2074564 |
| 2116131 | PPA1954 | - | 2116169 | 2116130 | 2115351 |
| 2365391 | PPA2183 | - | 2365465 | 2365391 | 2364734 |
| 2496196 | PPA2299 | - | 2496258 | 2496195 | 2495050 |
